# Activation of Inorganic Ligands by Interfacial Charge Transfer from a Complexation-Generated Valence Band at the Surface of a Metal Oxide Nanocrystal

**DOI:** 10.1021/jacs.6c10243

**Published:** 2026-08-04

**Authors:** Arti Joshi, Nitai Leffler, Shubasis Roy, Sourav Pramanik, Nisarani Bishoyi, Mark Baranov, Ira A. Weinstock

**Affiliations:** † Department of Chemistry, 26732 Ben-Gurion University of the Negev, Beer Sheva 84105, Israel; ‡ Ilse Katz Institute for Nanoscale Science & Technology, Ben-Gurion University of the Negev, Beer Sheva 84105, Israel

## Abstract

Incorporation of colloidal metal oxides into multicomponent solid-state materials gives rise to heterojunctions that, in photocatalysis, can obviate the need for sacrificial reagents. In principle, however, metal-oxide nanocrystals (NCs) could be functionalized by ligands that, while stabilizing the particles in solution, interact with the metal-oxide surface to provide self-contained analogs of multicomponent solid-state photocatalysts. We now report that simulated solar-light irradiation of 3.2 nm rutile-SnO_2_ NCs complexed by monolayers of approximately 50 hexaniobate anions, [Nb_6_O_19_]^8–^ (**1**) leads to the selective reduction of CO_2_ to CO by water alone. Mott–Schottky analysis of **1** gives band energies typical of rutile SnO_2,_ which, in relation to the HOMO and LUMO energies of hexaniobate, rule out Z-scheme or type-II heterojunction mechanisms. Ultraviolet photoelectron spectroscopy (UPS), however, revealed a new valence band (VB) nearly 2 eV higher in energy than the canonical VB energy of rutile SnO_2_. Topotactic mapping of Nb–O→Sn linkages from hexaniobate ligands to nearly all surface-accessible Sn atoms supports the generation of a network of electron-rich Sn-linked O atoms. Excitation of this surface-localized VB results in charge transfer (CT) conversion of the inorganic ligands into catalytic active sites. More generally, the findings introduce a new approach to interfacial engineering of metal-oxide NCs.

## Graphical abstract

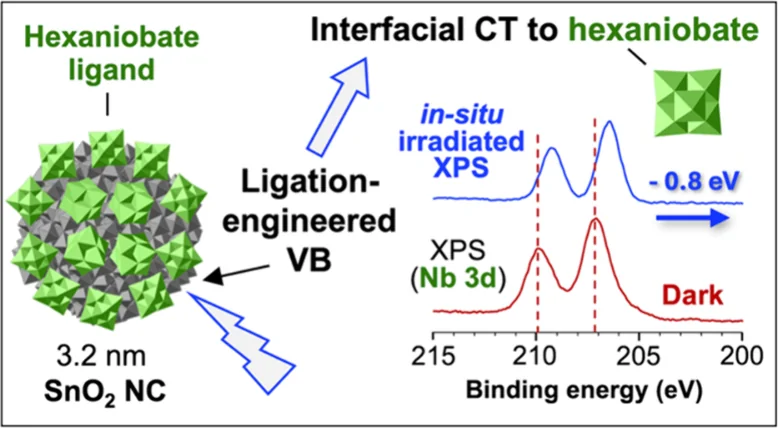

## Introduction

Unlike solid-state semiconductors, metal chalcogenide quantum dots (QDs) and metal-oxide nanocrystals (NCs) have large surface-area-to-volume ratios, such that modifications at the particle interface^1^ profoundly influence their electronic structure and related properties. For example, surface-band bending and valence and conduction band energies can be rationally tuned through ligation by dipolar ligands^2–5^ and solvation;^6^ control over exciton confinement in QDs can be induced by ligands with energetically aligned interfacial orbitals;^7^ free-carrier densities can be modulated via ligand-bonding;^8^ photocatalytic activity can be enhanced by ligand-hybridized interfaces,^9,10^ and depletion effects in metal-oxide NCs^11^ can be minimized by the deposition of insulating shells.^12^

In photocatalysis, this control over electronic structure and properties^13,14^ has enabled the use of QDs as visible-light sensitizers for electron transfer^15,16^ to proximally assembled metallo-organic complexes, which in the presence of electron donors, catalyze reductive transformations^17,18^ such as the selective, high-turnover reduction of CO_2_ to CO.^19^ And, although colloidal metal-oxides are photocatalysts for numerous oxidative and reductive transformations, they are unstable unless surrounded by organic ligands that unfortunately block access of substrates to reactive particle surfaces.^20^ Colloidal metal oxides are remarkably effective, however, when incorporated into multicomponent solid-state materials^21^ whose heterojunctions^22,23^ can obviate the need for sacrificial electron donors or acceptors.^24^

In principle, however, the surfaces of metal-oxide NCs could be functionalized by redox-active inorganic ligands^25^ that, while stabilizing the particles, interact with the metal-oxide core to provide self-contained analogs of multicomponent solid-state photocatalysts. Based on findings from solid-state and molecular science, we envisioned that this might be achieved by using hexaniobate anions, [Nb_6_O_19_]^8–^, to complex suitable metal-oxide NCs. This is because, in solid-state photocatalysis, irradiation of layered-polyniobate nanosheets with intercalated metal-oxide NCs^26^ catalyze redox transformations not observed for either the NCs or the polyniobates alone.^27–32^ And in molecular catalysis, solar-light excitation of cubic-phase NiO clusters coordinated by hexaniobate anions results in charge-transfer activation of the inorganic ligands, which reduce CO_2_ to CO, with holes in the NiO core simultaneously oxidizing water.^33^

We now report that 305 nm (cutoff) irradiation of 3.2 nm rutile-SnO_2_ NCs, each complexed by 50 hexaniobate anions (**1**),^33–36^ leads to the selective reduction of CO_2_ to CO using only water. This does not occur for rutile SnO_2_ alone, whose conduction-band (CB) energy^37^ is insufficiently negative, nor for pure hexaniobate clusters, wherein oxo-ligand to Nb^V^ charge transfer requires irradiation at 232 nm.^38,39^ Rather, as will be shown, topotactic alignment of electron-dense hexaniobate oxo-anions to ligand-accessible Sn atoms of **1** generates a surface-localized VB that serves as the source of electrons for charge-transfer activation of the redox-active inorganic ligands.

## Results

### Hexaniobate-Complexed SnO_2_ NCs

Complex **1** was synthesized by reaction of SnCl_4_ with K_8_Nb_6_O_19_ at 120 °C for 20 h at pH 12 (Figure S1). After purification (Figure S2), the yield of **1**, present as individual particles when imaged by cryo-TEM (Figure 1A), was 54%, based on Sn. Dynamic light scattering (DLS) measurements (upper inset to Figure 1A) indicated an average diameter of 3.2 ± 0.6 nm, while a histogram based on analysis of 50 particles observed by cryo-TEM gave a size of 3.2 ± 0.2 nm (Figure S3 and S4).

**Figure 1:**
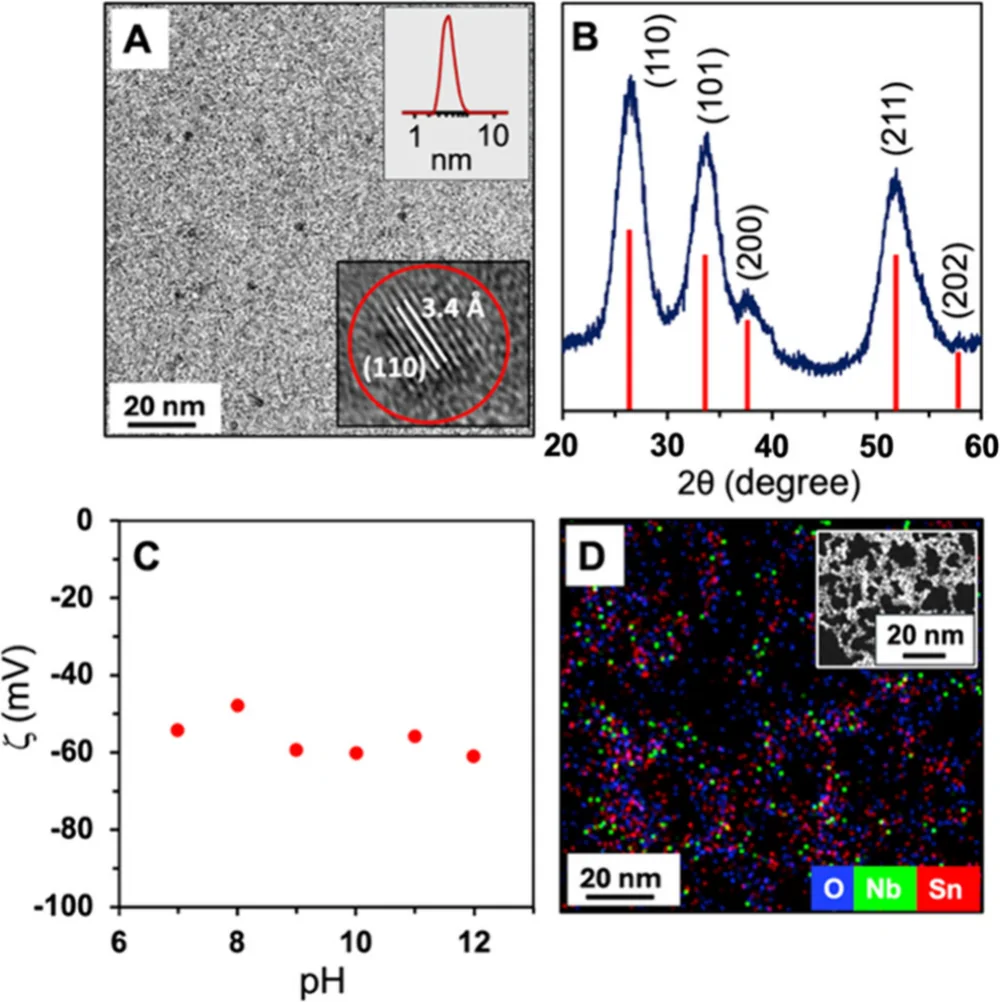
(A) Cryo-TEM image of **1** in water; upper inset: number-weighted DLS; lower inset: HRTEM with lattice fringes indexed to (110) *d*-spacing of rutile SnO_2_. (B) Powder XRD pattern of **1** (blue curve) and simulated pattern for tetragonal SnO_2_ (red lines). (C) Zeta potentials of **1** are effectively constant over a wide pH range. (D) EDX elemental mapping of dried **1** (inset), confirming a homogeneous distribution of O (blue), Nb (green), and Sn (red).

The rutile phase of the SnO_2_ core of **1** was determined by high-resolution TEM (lower inset to Figure 1A) and Figure S5) and by powder X-ray diffraction (XRD) (Figure 1B and Figure S6). High-resolution X-ray photoelectron spectroscopy (XPS) revealed only Sn^4+^ with no indication of Sn^2+^ or Sn^0^ (Figure S7). Solutions of **1** are optically transparent with no precipitation even at 1 mM particle concentrations. This solubility, combined with the negative and nearly pH-independent zeta potential of ζ = −55 ± 5 mV (Figure 1C), is consistent with previously reported hexaniobate-^34^ and heteropolytungstate-complexed metal-oxide,^25,40^ oxyhydroxide,^41^ and hydroxide^42^ NCs.

Energy-dispersive X-ray spectroscopy (EDS) with elemental mapping confirmed a homogeneous distribution of Sn, O, and Nb (Figure 1D and Figure S8). The FTIR spectrum of **1** is consistent with ligation by Nb_6_O_19_^8–^ (Figure S9 and S10), whose terminal Nb=O and bridging Nb–O–Nb moieties give rise to bands at 860 and 400–750 cm^–1^, respectively (see Figure S11 for Raman spectra). Additional support for intact Nb_6_O_19_^8–^ ligands was provided by FTIR analysis of unreacted hexaniobate in the synthetic reaction mixture after precipitation of **1**, and after heating K_8_Nb_6_O_19_ at 120 °C for 20 h at pH 12, identical conditions to those used to prepare **1** (Figure S10).

The average number of hexaniobate ligands on each complex, **1**, was calculated using the size of the SnO_2_ core to fit data from elemental analysis, which gave an atom-percent ratio of Sn:Nb = 61:39. Based on 3.2 ± 0.2 nm SnO_2_ cores (480 ± 90 Sn atoms), the Sn:Nb ratio corresponds to an average of 50 ± 0.7 hexaniobate ligands on each SnO_2_ NC (Table S1). On average, each ligand is allocated an area of 0.64 nm^2^ on the SnO_2_ NC surface, 28% larger than the 0.5 nm^2^ crystallographic “footprint” of the 0.8 nm diameter hexaniobate anion.^43,44^ This leaves ca. 0.5 nm between neighboring hexaniobate ligands, sufficient to accommodate numerous K^+^ counterions. This results in a near-monolayer at the particle surface.

In solid-state polyniobates, Mallouk found that intercalated metal-oxide NCs,^29^ exemplified by Rh_2_O_3_, are stabilized by the formation of Nb−μ-O–Rh linkages,^28^ and by charge transfer from the metal oxide to the polyniobate.^27^ We used X-ray photoelectron spectroscopy (XPS) to investigate whether related oxo linkages might be present at the surface of **1**. For this, XPS spectra of Nb 3d orbitals were obtained for pure hexaniobate (K_8_Nb_6_O_19_) and for **1**, illustrated at the far left of Figure 2, with the ligands intentionally separated from one another for clarity.

**Figure 2:**
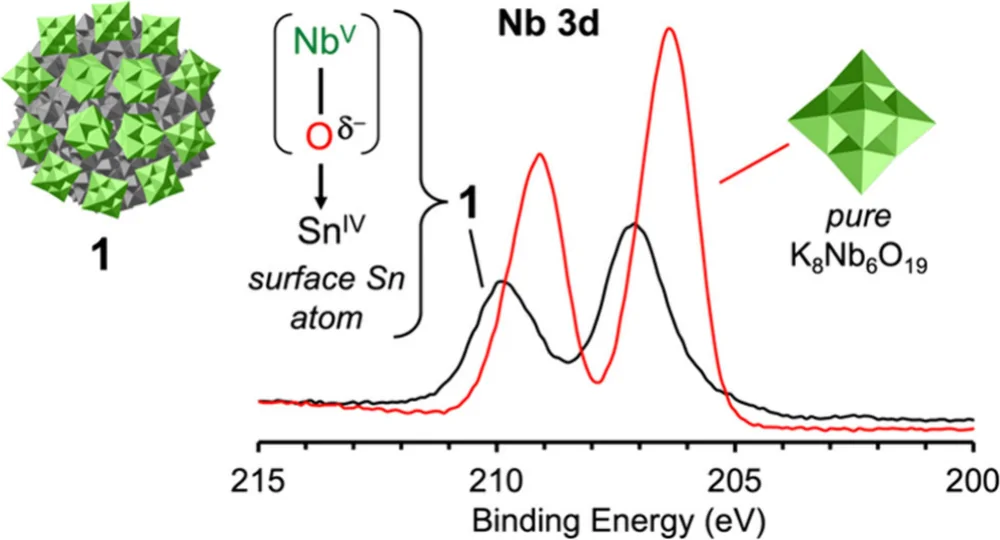
XPS spectra of the Nb 3d orbitals of hexaniobate (red curve), and their shift to 0.8 eV in the positive direction, i.e., to larger binding energies, when the anion is ligated to 3.2 nm SnO_2_ NCs in **1** (black curve). Far-left inset: Illustration of **1**, with the ligands intentionally separated from one another for clarity. Electron donation from oxo ligands of hexaniobate to Sn^IV^ at the surface of the SnO_2_ core of **1**.

For pure hexaniobate (red curve in Figure 2), binding energies for Nb 3d_5/2_ and Nb 3d_3/2_ spin–orbit states were 206.3 and 209.1 eV, respectively. After complexation to SnO_2_ in **1**, however, these binding energies were 207.1 and 209.9 eV, respectively, both shifted to more positive values by 0.8 eV. This increase in binding energy, indicative of electron withdrawal from Nb^V^, implies the formation of Nb−μ-O–Sn linkages (left inset of Figure 2), albeit opposite in polarity to those documented by Mallouk.^27^ As discussed below, these oxo-donor linkages fundamentally change the nature of the SnO_*x*_ network at the NC surface (see Figure S7 for additional comments).

In situ XPS^45^ was then used to investigate whether charge transfer to Nb^V^ could be detected upon irradiation at 280 nm, a wavelength less energetic than the 230 nm needed to promote oxo-ligand to Nb^V^ charge transfer in pure hexaniobate. The in situ XPS data (Figure 3A) revealed an unusually large^46^ shift of 0.8 eV toward lower binding energies, indicative of charge transfer to Nb^V^. Importantly, no shift in binding energy was observed upon 280 nm irradiation of pure hexaniobate (Figure S12). Due to the absence of detectable photoluminescence, it was not possible to measure the lifetime of the photogenerated excited state.

**Figure 3:**
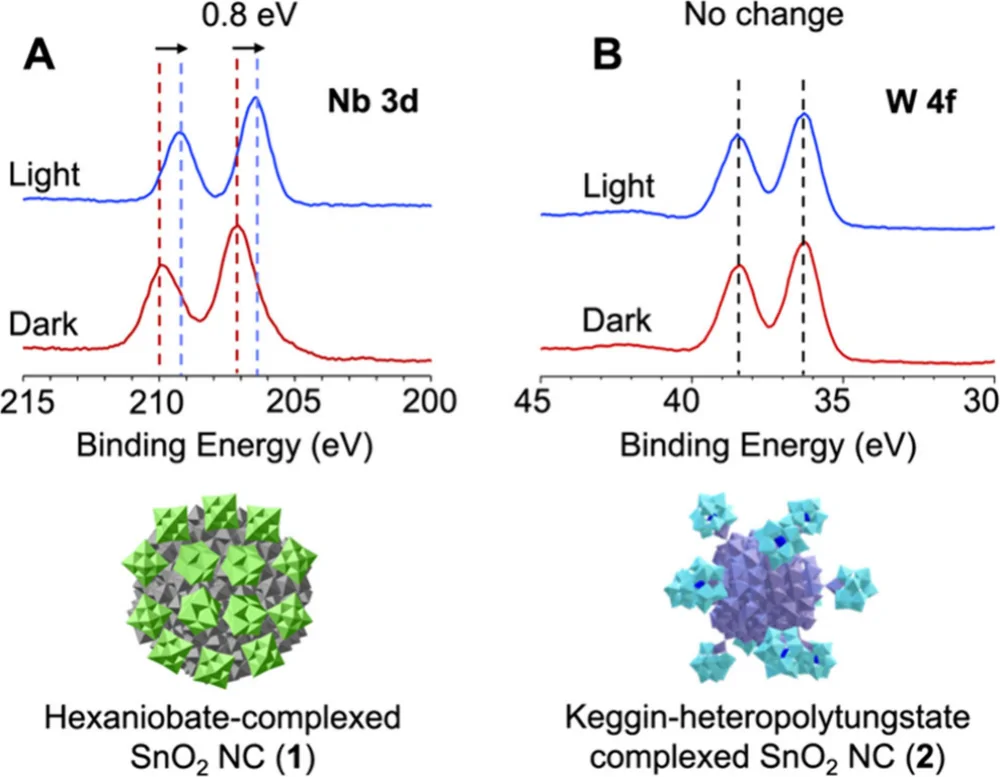
(A) XPS spectra of **1** in the dark (red curve) and under irradiation at 280 nm (blue curve), which shifted the binding energies for Nb 3d_5/2_ and Nb 3d_3/2_ states, respectively, from 207.3 to 206.5 and from 210.0 to 209.2 eV. (B) Identical conditions failed to promote charge transfer to the tungsten-based ligands of **2**. For comparison, structures of **1** and **2** are shown directly below the corresponding spectra.

The same in situ XPS experiment was carried out using a Keggin-heteropolytungstate analog of **1**, wherein 3.6 nm SnO_2_ NCs are each complexed by an average of 10 oxo-donor ligands, [PW_11_O_39_Sn^IV^]-O^–^ (**2**, Figure 3B).^47^ Although W­(VI) atoms in heteropolytungstates are reduced at potentials at least 1 V^48^ more positive than that of Nb^V^, no change in binding energies of W 4f orbitals was observed. Importantly, complexation to the SnO_2_ surface differs substantially between the ligand types. Only 10 Keggin-ion ligands are bound to the SnO_2_ cores of **2**, each via a single μ-oxo linkage. By contrast, single-crystal X-ray diffraction analysis of molecular hexaniobate-complexed metal-oxide clusters^33,35^ shows that multiple oxo ligands of each hexaniobate anion can bind to different metal atoms. And for **1**, ca. 50 hexaniobate ligands are bound to the SnO_2_ core. Because interfacial charge transfer was not observed by XPS for **2**, the latter complex was used below as a control to clarify the reactivity of the rutile-SnO_2_ core of **1**.

### Catalysis by Light-Activated Hexaniobate Ligands

We then explored whether charge transfer to the hexaniobate ligands could support photocatalytic transformations of electron-accepting substrates. Because hexaniobate anions readily complex metal cations,^43,44,49^ we hypothesized that the photochemical reduction of complexed Cu^2+^ ions might provide a sensitive test for initial screening. This is because precoordination should significantly enhance the rate at which electrons in photoreduced hexaniobate ligands are productively trapped by Cu^2+^.

To establish this precoordination, **1** (1.76 μM) was reacted with Cu^2+^ (0.5 mM, five equiv per hexaniobate ligand). This led to the assembly of soluble aggregates, as indicated by an increase in DLS scattering intensity (Figure S13). Evidence for direct complexation of Cu^2+^ was obtained by isothermal titration calorimetry (ITC, Figure 4A, top and Figure S14–S15), which revealed the reaction to be endothermic, i.e., entropically driven.

**Figure 4:**
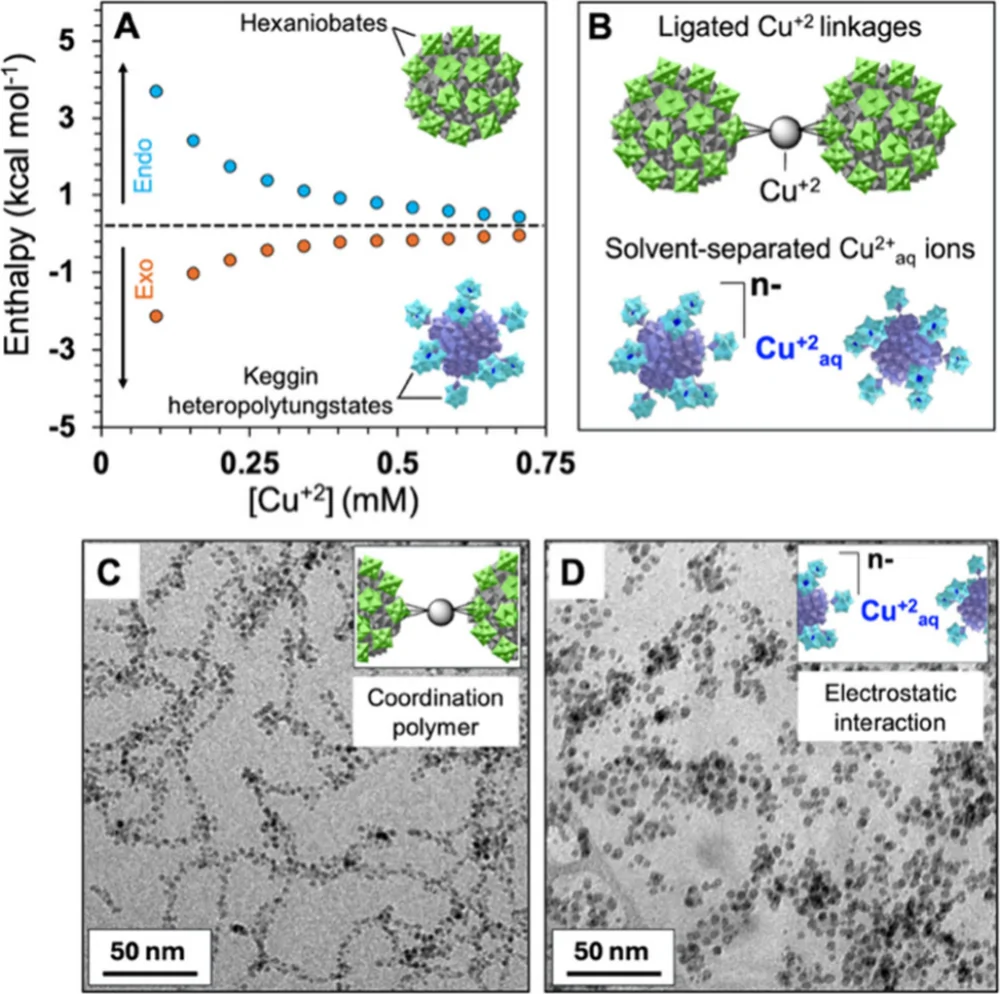
(A) ITC data for additions of Cu^2+^ to **1** (top) and to **2** (bottom). (B) Models for endothermic, entropically driven complexation of dehydrated Cu^2+^ by **1** (top), and for exothermic solvent-separated ion pairing with **2** (bottom). (C) and (D), respectively, are cryo-TEM images of Cu^2+^-linked coordination polymers of **1**, and random aggregates of **2** formed via electrostatic interaction with hydrated Cu^2+^ ions.

Quantitative analysis of the ITC data using an independent-site model^50^ revealed a change in enthalpy of ΔH = +23.6 kcal mol^–1^, offset by a larger increase in entropy for which *T*Δ*S* = −27.9 kcal mol^–1^ (298 K), summing to a negative change in Gibbs free energy of ΔG = −4.3 kcal mol^–1^ (Table S2). The favorable entropy change indicates complexation-driven dehydration of the metal cation, yielding Cu^2+^-linked NCs, as illustrated at the top of Figure 4B. Additional confirmation was provided by cryo-TEM images, which revealed branched, linear coordination polymers of linked NCs (Figure 4C and Figures S17–S18).

Additional confirmation of precoordination by **1** was provided by comparing ITC data for the reaction of Cu^2+^ with **2** (Figure 4A, bottom). While this also led to soluble aggregates (see DLS data in Figure S19), ITC data revealed an energetic signature opposite to that obtained for **1** (Figure S16 and Table S2). Namely, a negative enthalpy change of Δ*H* = −13.4 kcal mol^–1^ was attenuated by a *decrease* in entropy of −*T*Δ*S* = +9.3 kcal mol^–1^ (298 K), summing to Δ*G* = −4.1 kcal mol^–1^. These values are typical of electrostatic interactions between hydrated Cu^2+^ ions and the heteropolytungstate ligands of **2** (bottom of Figure 4B). These interactions gave disordered aggregates observed by cryo-TEM (Figure 4D and Figures S20–S21).

Having confirmed precoordination of Cu^2+^ by **1**, the resultant polymers in 5% aqueous 2-propanol (Figure 5A), were irradiated under Ar using a 150 W Xe lamp fitted with a 305 nm cutoff filter. After 2 h, all of the Cu^2+^ had been reduced to colloidal Cu(0) NPs (Figure 5B).

**Figure 5:**
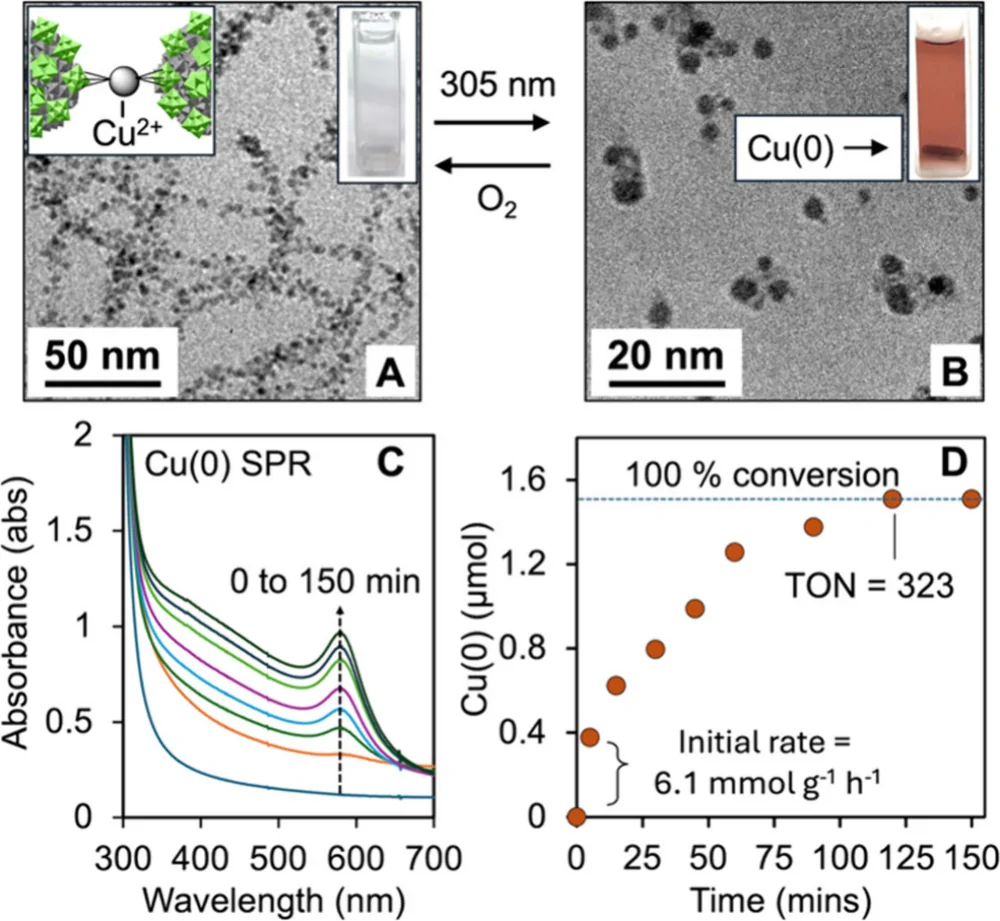
Xe-lamp irradiation (305 nm cut off) of (A) Cu^2+^-linked coordination polymers of **1** in 5% *i*PrOH. After 150 min, all the Cu^2+^ present had been reduced to Cu(0) NPs (B). Images in both panels are from cryo-TEM, and the upper-right insets are photos of the two solutions. (C) UV–vis spectra obtained during Cu^2+^ reduction were used to obtain a plot of Cu(0) formation per time (D). Under these conditions, no Cu^2+^ reduction was observed for **2**.

Reduction to Cu(0) was confirmed by UV–vis spectroscopy (Figure 5C and Figure S22) and XPS (Figures S23–S24). Possible stabilization of Cu(0) NPs by **1** is discussed in relation to Figures S25–S26. Complete conversion of Cu^2+^ to Cu(0) was determined by the final SPR absorbance at 580 nm, giving a TON of 323 per equiv **1**, with an initial rate of 6.1 mmol Cu(0) g^–1^ h^–1^ (see Figure S22 and related discussion). The apparent quantum yield (AQY), based on the initial rate, is 3.04% (see Methods in the SI). The catalytic role of **1** was highlighted by the spontaneous reassembly of the original coordination polymers upon air oxidation of Cu(0) to Cu^2+^ (see cryo-TEM images in Figure S27–S28). No Cu^2+^ reduction occurred when **2** or pure hexaniobate were used (Figures S29–S30).

Complex **1** was then irradiated using the same 305 nm cutoff filter, but now in pure water under Ar. This generated two equiv of H_2_ per mol of O_2_ (Figure 6A). Repeat of the reaction using the same sample gave identical results. No changes in powder XRD patterns were observed before and after reaction (Figure S33). No products were observed in control experiments using aqueous solutions of either hexaniobate anions or **2**. To our knowledge, solar-light-driven water oxidation by SnO_2_ NCs has not been reported; it is facilitated here by charge transfer to hexaniobate.

**Figure 6:**
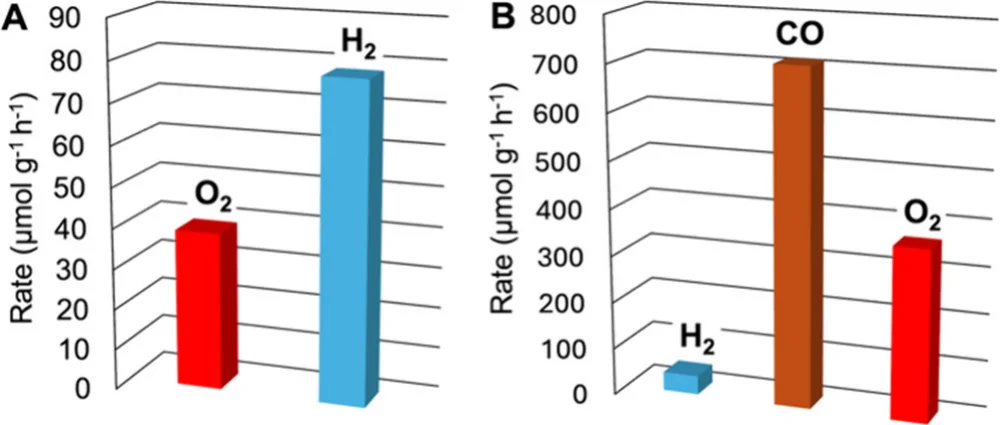
Batch reactions of **1** in pure water, under Xe-lamp irradiation (305 nm cut off) with no added sensitizers or sacrificial agents. (A) Under Ar, water was split into H_2_ and O_2_ in a 2:1 molar ratio (8 h of irradiation) The AQY is 1.71% (see Methods in the SI). (B) Under 1 atm of CO_2_, 4 h irradiation gave 2.14, 0.12, and 1.1 molar equiv, respectively, of CO, H_2,_ and O_2_ (Figure S31); the AQY is 2.38% (see Methods in the SI). For **2**, no reaction was observed in pure water or in the presence of CO_2_.

Because the hexaniobate ligands of **1** are polybasic,^44^ they bind protons as countercations, kinetically facilitating H_2_ evolution upon interfacial charge transfer. A more challenging test involved the binding and catalytic reduction of CO_2_ in pure water. A 4-h batch reaction (305 nm cut off) gave CO at 94% selectivity relative to H_2_ (Figure 6B). Notably, the moles of CO and H_2_ combined are 2.05 times the moles of O_2_, representing a 1:1.03 stoichiometry based on electron equivalents. No changes in powder XRD patterns were observed before and after reaction (Figure S34). Repeating the reaction three times with the same sample yielded effectively identical results in each case (Figure S31). No reaction was observed for **2** (Figure S32), and to our knowledge has not been reported for SnO_2_.

By analogy to simulated solar-light reduction of CO_2_ by molecular hexaniobate complexes of cubic-NiO clusters, for which a detailed DFT study was performed,^33^ charge transfer activation of the hexaniobate ligands increases electron density at the anion’s terminal and bridging O atoms, which then serve as nucleophillic sites for CO_2_ binding. This is followed by proton-coupled electron transfer by adjacent Nb^IV^ sites to give CO and H_2_O.

## Discussion

### Hexaniobate Activation

Neither of the reactions in Figure 6 has been reported for SnO_2_ and are not observed for **2**. This establishes hexaniobate ligation as key to the photocatalytic activity of **1**. The question addressed in this section is how that is best understood.

Based on the ability of ligands to modify VB, CB, and band gap energies of QDs and metal-oxide NCs, we first considered whether a similar phenomenon might explain the reactivity of **1**. As will be shown, this was ruled out by analysis of the VB and CB energies of the SnO_2_ cores of **1**, and by comparison with the HOMO and LUMO energies of pure hexaniobate. First, Mott–Schottky analysis based on impedance spectroscopy of **1** indicated n-type rutile SnO_2,_ with the bands shown in Figure 7A (Figure S35). These match the valence and conduction bands typical of n-type rutile SnO_2_.^37^ The valence-band maximum (VBM) is mainly composed of O 2p states, while the conduction-band maximum (CBM) involves Sn 5s and O 2s states. Due to dipole-forbidden transitions, the optical band gap is between 3.6 and 3.7 eV, somewhat larger than the fundamental band gap of 3.0 eV.^37^ The CB energy of SnO_2_, which lies at −0.1 V vs NHE (Figure 7A, entry I), is not sufficiently negative in practice to reduce H^+^ nor to reduce CO_2_, and explains why those reactions have not been reported for SnO_2_. This lack of reactivity is reflected in control experiments using **2**, included here as a control for the reactivity of the SnO_2_ core of **1** (see Figure 3).

**Figure 7:**
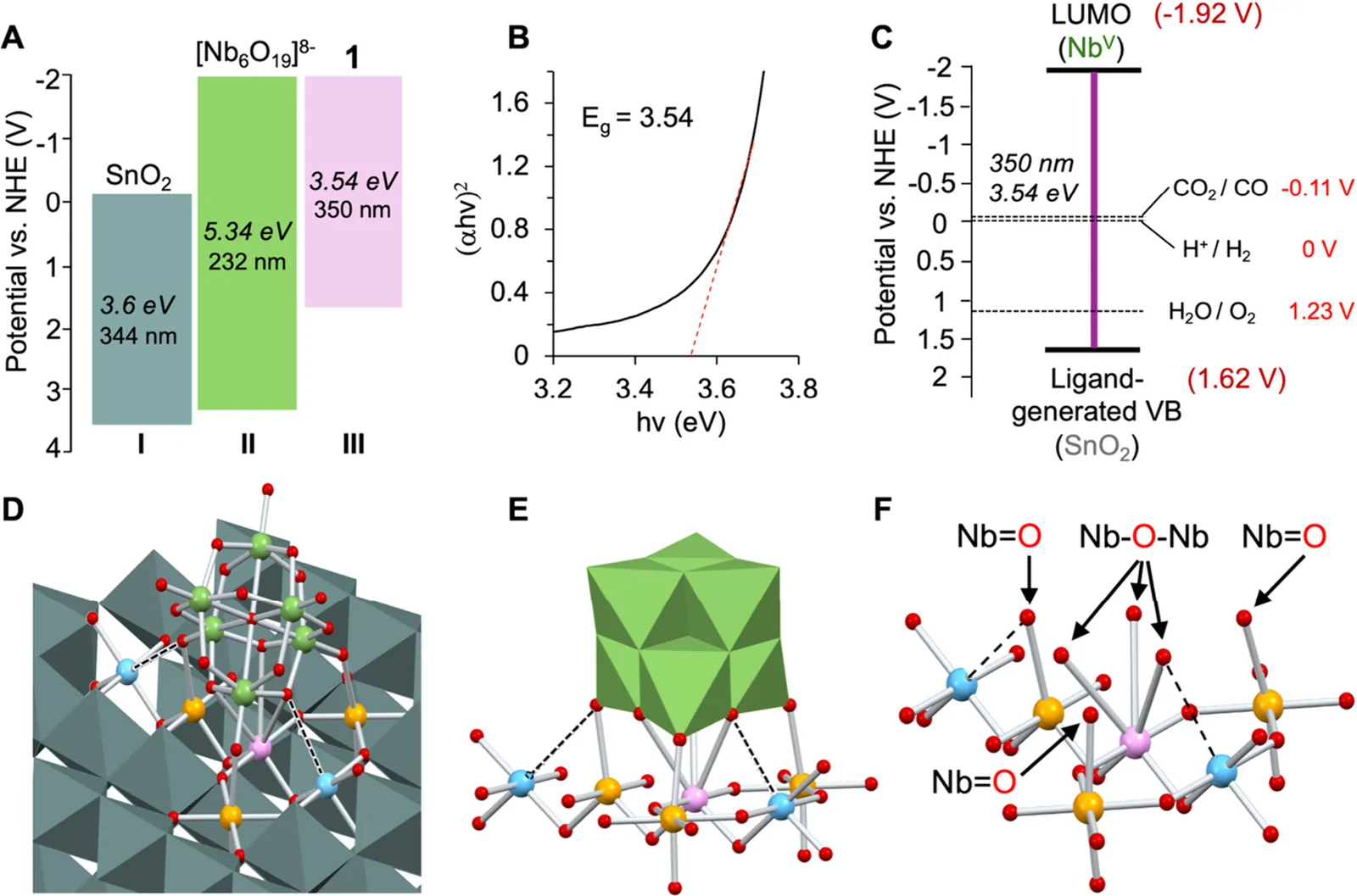
(A) Comparison of the electronic energy levels of the rutile SnO_2_ core of **1**, based on impedance spectroscopy and optical measurements (**I**), of hexaniobate ligand LMCT excitation (**II**), and of the ligand-engineered interfacial band structure of **1 (III)**, the latter constructed based on UPS and optical measurements. See text for details. (B) A Tauc plot used to determine an optical band gap of 3.54 eV for **1**. (C) Band alignment of **1** relative to the redox potentials for CO_2_ reduction and water splitting. (D–F) Structural models drawn to scale to illustrate topotactic mapping between hexaniobate oxo-donor ligands and accessible Sn atoms at the SnO_2_ NC surface. (D) A ball and stick representation of hexaniobate, with Nb in green and O in red, on a 110 facet of rutile-SnO_2_, shown in polyhedral notation. Specific Sn atoms spatially aligned with oxo-donor ligands of hexaniobate are color-coded as follows: pink for a Sn atom coordinated in a tridentate fashion by three bridging O atoms in a triangular face of the anion, and gold for three Sn atoms coordinated by terminal Nb=O ligands. The graphically defined bond lengths are reasonable, ranging from 2.2 to 2.4 Å. The blue-colored Sn atoms may form weaker interactions with terminal Nb=O atoms, but these might not be present in practice when the high curvature of the 3.2 nm SnO_2_ is considered. The three types of interactions are highlighted in (E) by removing most of the rutile-SnO_2_ structure and rendering the hexaniobate anion in polyhedral notation. (F) Illustrates the spatial organization of bridging and terminal oxo-donor ligands of the hexaniobate anion in relation to ligand accessible Sn atoms.

The HOMO and LUMO energies associated with ligand-to-metal charge transfer (LMCT) excitation of electrons from oxo ligands^39^ of the hexaniobate anion to the 4d orbitals of Nb^V^ are shown in Figure 7A, entry II. At pH 10, typical for solutions of **1**, the excitation maximum^38^ lies near 230 nm (5.34 eV). The LUMO energy was set equal to the reduction potential of the hexaniobate anion, estimated from published data^51,52^ to be near −1.9 V vs NHE. The same value was obtained by combining Tauc and Mott–Schottky analyses of impedance spectroscopy data for a molecular hexaniobate complex of a cubic (NiO)_30_ cluster.^33^ The LUMO and 5.34 eV excitation energies place the HOMO at ca. +3.4 V as shown in Figure 7A.

Considering the relative energies of the CB in SnO_2_ and of the LUMO of hexaniobate (entries I and II in Figure 7A), excitation of the rutile SnO_2_ core of **1** does not result in charge transfer to Nb^V^. And, as noted above, Mott–Schottky analysis of **1** gave the canonical valence and conduction band energies of rutile SnO_2_ (Figure 7A, entry I), thus ruling out significant ligation-induced changes to those states in the complex’s SnO_2_ core.

Finally, the 305 nm cutoff filter used in catalysis (Figures 5 and 6) is insufficient to promote LMCT within the hexaniobate ligands. This not only explains why the hexaniobate anions alone are inactive but also precludes their electron acceptance from the CB of SnO_2,_ as is typical of Z-Scheme heterojunctions,^35,53^ while the relative band positions rule out Type-II heterojunction behavior.^35^

### A Ligand-Engineered Valence Band

The above analysis shows that excitation of the SnO_2_ core of **1** cannot account for charge-transfer activation of the hexaniobate ligands. We thus considered whether electron donation from the hexaniobate ligands to surface Sn^IV^ atoms via Nb−μ-O–Sn linkages (inset to Figure 2) might sufficiently increase charge densities at surface-Sn coordinated O atoms to generate a new VB at an energy higher than that of the VB of the rutile-SnO_2_ core. If this were the case, the 350 nm activation of hexaniobate would be assigned to charge transfer to Nb^V^ from ligand-perturbed surface O atoms, along with holes localized mainly on the SnO_2_ surface. Importantly, charge transfer from the electron-donating oxo ligands of the hexaniobate can be ruled out because, due to electron donation to Sn^IV^, it would require larger energies than the 5.34 eV indicated in Figure 7, entry II.

To evaluate the possible presence of a higher-energy VB, ultraviolet photoelectron spectroscopy (UPS) was used to determine the work function for light-induced electron ejection.^54^ The data (see Figure S36 and Supplementary calculation S1) indicated the presence of a VB at −1.62 V. The combination of this with the Tauc-derived band gap of 350 nm (Figure 7B) gave the purple-colored band in Figure 7A, entry III. Notably, the excited-state maximum at −1.9 eV is effectively identical to the energy of the LUMO of hexaniobate (entry II in Figure 7A). This implies that the band gap in entry III is the energy required to excite electrons from the ligand-engineered VB, identified by UPS, to Nb^V^. In Figure 7C, this band is shown in relation to energies required for the catalytic transformations shown in Figure 6.

We then sought a structural basis for the ligand-engineered VB at the particle surface. The first task was to assess the degree of alignment possible between terminal and bridging hexaniobate oxo groups and ligand-accessible Sn atoms at the NC surface. This was done by setting crystal structures of rutile SnO_2_ and hexaniobate anions to a common size scale. The hexaniobate anion was then placed triangular-face down on a rutile-SnO_2_ surface. Given that 50 ± 0.7 hexaniobate ligands are bound to each 3.2 ± 0.2 nm SnO_2_ core of **1**, a flat {110} facet, the most dominant family of faces in rutile SnO_2_, was used to approximate the rutile-SnO_2_ surface, represented in Figure 7D by gray polyhedra. The hexaniobate ligand is shown using a ball-and-stick representation, with Nb atoms in green and oxygen atoms, including those of SnO_2_, in red. Surface Sn atoms close enough to interact with the hexaniobate oxo ligands are shown as colored spheres at the SnO_2_ surface.

Figure 7E shows interactions between a triad of three bridging oxo ligands within hexaniobate, now in polyhedral notation, and a tricoordinated Sn atom, in pink, at the NC surface. This coordination motif is common to hexaniobate complexes of metal cations.^49^ The gold-colored Sn atoms are sites of ligation by terminal, Nb = O oxo ligands. For clarity, the oxo-donor ligands and their spatial mapping to Sn atoms at the NC surface are labeled in Figure 7F, and presented without the remainder of the hexaniobate cluster.

From the spatial mapping in Figure 7E-F, it emerges that a single hexaniobate ligand can interact strongly with four oxo-linked Sn atoms. In order to generate an ordered VB at the NC surface, most of the ligand-accessible Sn atoms should ideally be coordinated by hexaniobate oxo-donor ligands. The number of accessible Sn atoms was calculated to be 200 ± 20 (Supplementary calculation S2). Because each NC is ligated by a near-monolayer of 50 hexaniobate ligands, most or all of the surface Sn atoms can serve as Lewis-acid acceptors of electron density from oxo-donor ligands of the inorganic anions. Moreover, considering that each of the 200 Sn atoms is coordinated by four chemically and symmetry-equivalent O atoms, oxo-donor ligation by 50 hexaniobate anions could generate a surface-localized VB network of ca. 400 O atoms with electron densities larger than those of O atoms within the rutile-SnO_2_ core (Supplementary calculation S3).

Thus, rather than modifying the canonical VB and CB energies of the rutile-SnO_2_ core of **1**, the hexaniobate ligands generate a new surface-localized VB band from which electrons are promoted to Nb^V^, and which serves as the initial site of simultaneously generated holes.

Finally, excitation from the surface-localized VB of **1** (at −1.61 eV) to the canonical CB of rutile SnO_2_ (−0.1 eV) would require 1.72 eV and give rise to a band at 720 nm in the UV–visible spectrum. No band appears in the visible region, thus ruling out direct excitation from the surface-localized VB band to the rutile SnO_2_ CB.

An interesting question is why this transition does not occur. A possible explanation is that spatial proximity of the surface-localized VB to the hexaniobate ligands allows for mixing between Sn–O donor states and Nb­(V), leading to strong donor–acceptor coupling. As such, charge-transfer excitation would remain entirely within the hybrid interface, thus bypassing the SnO_2_ CB, comprised of Sn 5s and O 2s states^37^ in the rutile core. After charge-transfer reduction of Nb­(V), indirect population of the SnO_2_ CB should be kinetically inhibited by the reorganization energy^48^ associated with oxidation of Nb­(IV) to Nb­(V).

## Conclusions

We show here that complexation by inorganic acceptor ligands can convert metal-oxide NCs into self-contained analogs of multicomponent solid-state photocatalysts. This was achieved using water-soluble complexes **1**, comprising 3.2 nm rutile-SnO_2_ cores each ligated by an average of 50 hexaniobate anions. Light-induced charge-transfer to the hexaniobate ligands was documented by in situ irradiated XPS (Figure 3), which revealed an unusually large 0.8 eV decrease in Nb 3d binding energies. Resultant photoactivation of the inorganic ligands of **1** was initially confirmed by the catalytic reduction of **1**-complexed Cu^2+^ ions in the presence of *i*PrOH. In pure water, **1** produced H_2_ and O_2_ at a 2:1 ratio, and selectively reduced CO_2_ to CO (94%) and H_2_ (6%), along with 1 ± 0.03 electron equivalents of O_2_. No reactions were observed in control experiments using **2**, for which ca. 10 Keggin heteropolytungstate anions are each coordinated by single μ-oxo linkages to 3.6 nm SnO_2_ NCs.

Mott–Schottky analysis of **1** using impedance spectroscopy gave valence and conduction band energies typical of rutile SnO_2_ (Figure 7A, entry I). Comparison of this band structure to the 5.34 eV needed for LMCT within hexaniobate, and the −1.9 V energy of its Nb^V^-based LUMO (Figure 7A, entry II), ruled out Z-scheme or type-II heterojunction mechanisms. However, ultraviolet photoelectron spectroscopy (UPS) identified a new VB (Figure 7A, entry III) nearly 2 V higher in energy than that of the rutile-SnO_2_ core of **1**. The origin of this band is understood by topotactic mapping of the highly electron-dense oxo ligands of hexaniobate with the NC surface (Figure 7, D-F), which showed that most or all of the 200 ligand-accessible Sn atoms can serve as Lewis-acid sites for the formation of Nb–O→Sn linkages. This, in turn, generates a surface-localized VB network of ca. 400 O atoms whose electron densities are larger than those of O atoms within the rutile-SnO_2_ core. This surface-localized VB band is the source of electrons promoted to the Nb^V^-based LUMO of hexaniobate and the concommitent formation of holes at +1.6 V (Figure 7C) that serve as sites for water oxidation.

In summary, photoactivation of the hexaniobate ligands of **1** is not due to modification of the canonical band structure of its rutile-SnO_2_ core, nor to interfacial phenomena typical of type-II or Z-scheme heterojunctions. Rather, coordination of electron-dense oxo-donor ligands to accessible Sn atoms generates a surface-localized VB capable of interfacial charge transfer that converts the reversibly reducible inorganic ligands into catalytic active sites. More generally, the findings introduce a new approach to interfacial engineering of metal-oxide NCs.

## Supplementary Material



## Notes

Detailed experimental procedures, characterization methods, and additional figures and tables (PDF)
