# Supplementary material for: Activation of Inorganic Ligands by Interfacial Charge Transfer from a Complexation-Generated Valence Band at the Surface of a Metal Oxide Nanocrystal
Source: J Am Chem Soc. 2026 Aug 4;148(32):34771–9. doi: 10.1021/jacs.6c10243 (PMC13495745; doi:10.1021/jacs.6c10243)
Supplement: Supplementary file 1 [file ja6c10243_si_001.pdf]

Supporting Information for:

Activation of Inorganic Ligands by Interfacial Charge Transfer from a Complexation-Generated Valence Band at the Surface of a Metal Oxide Nanocrystal

Arti Joshi,[1] Nitai Leffler,[1] Shubasis Roy,[1] Sourav Pramanik,[1] Nisarani Bishoyi,[1] Mark Baranov,[2] and Ira A. Weinstock*[1,2]

[1]Department of Chemistry, Ben-Gurion University of the Negev, Beer Sheva 84105, Israel.

[2]Ilse Katz Institute for Nanoscale Science & Technology, Ben-Gurion University of the Negev, Beer Sheva 84105, Israel.

Table of Contents

Materials and methods
Fig. S1. Synthetic scheme for preparing **1**
Fig. S2. Purification of **1**
Fig. S3. Cryo-TEM images of **1**
Fig. S4. Histogram obtained from cryo-TEM images of **1**
Fig. S5. HR-TEM of **1**
Fig. S6. PXRD comparison of **1** vs bare SnO_2
Fig. S7. XPS of **1**
Fig. S8. STEM-HAADF/EDX mapping of **1**
Fig. S9. FTIR spectra of **1**, hexaniobate, and recovered from **1** supernatant
Fig. S10. FTIR spectra comparison of hexaniobate: as-made and heat-treated
Fig. S11. Raman spectra comparing free hexaniobate, bulk SnO_2, and **1**
Table S1. Calc.of the number of Sn atoms and hexaniobate ligands in **1**
Fig. S12. *In situ* Nb 3d XPS of hexaniobate only before and after 280 nm illumination
Fig. S13. DLS measurements after addition of Cu^{2+} to **1**
Fig. S14. ITC thermograms of Cu^{2+} titration with **1** and **2**
Fig. S15. Integrated ITC enthalpy-change values for Cu^{2+} interaction with **1**
Fig. S16. Integratsed ITC enthalpy-change values for Cu^{2+} interaction with **2**
Table S2. Thermodynamic Parameters from ITC values for Cu^{2+} addition
Fig. S17. Cryo-TEM images of assemblies of **1** with Cu^{2+}
Fig. S18. Cryo-TEM images after heating the assemblies of **1** with Cu^{2+}
Fig. S19. DLS measurements after addition of Cu^{2+} to **2**
Fig. S20. Cryo-TEM images of **2** aggregated by the addition of Cu^{2+}
Fig. S21. Cryo-TEM images comparing Cu^{2+}-induced assemblies
Fig. S22. UV-Vis spectra during catalytic reduction of Cu^{2+} by **1**
Fig. S23. High-resolution Cu 2p XPS of Cu-containing assemblies of **1**
Fig. S24. Cu 2p XPS of reduced sample
Fig. S25. Cryo-TEM images after photoreduction showing Cu^0 particles
Fig. S26. Cryo-TEM images of hexaniobate-complexed CuO NCs on Au NPs
Fig. S27. Cryo-TEM images after air exposure to a solution of **1** and Cu^0 NPs
Fig. S28. Cryo-TEM image series showing reversible reduction of Cu^{2+}
Fig. S29. Control UV-Vis for Cu^{2+} reduction in the presence of **2**
Fig. S30. Control UV-Vis for Cu^{2+} reduction in the presence of only hexaniobate
Fig. S31. Recycling performance of **1**
Fig. S32. Control experiment with FTO alone and **2**
Fig. S33. PXRD pattern of **1** before and after water splitting
Fig. S34. PXRD pattern of **1** before and after CO_2 reduction
Fig. S35. Mott-Schottky analysis of **1**
Fig. S36. Ultraviolet photoelectron spectroscopy (UPS) analysis of **1**
Supplementary calculation S1. Calculation of VB and CB positions of **1** from UPS
Supplementary calculation S2. Number of ligand-accessible Sn atoms
Supplementary calculation S3. Influence of hexaniobate on surface-SnO_2 O atoms
References

Materials and methods

Materials

All materials were purchased as reagent grade and used without further purification. Tin chloride ($SnCl_4.5H_2O$, Thermo Scientific - ACROS), Niobium pentoxide (Nb_2O_5, CBMM niobium, USA), Potassium hydroxide (KOH, Strem Chemicals, Inc., USA), KCl (analytical grade, Frutarom, Israel), Potassium bromide (KBr for IR spectroscopy, Fisher Chemical, UK), Copper nitrate trihydrate (Sigma), Isopropyl alcohol (99.8%, Alfa-Aesar). Regenerated-cellulose dialysis membranes (45-mm flat-width tubes; 12-14000 Da MWCO) were purchased from VWR Scientific, treated before use to remove glycerin and traces of sulfur compounds, and stored in water at 5 °C. All water used for cleaning, synthesis, and reactions was of high purity (18.2 MΩ resistivity) from a Millipore® Direct-Q water-purification system. The polyoxometalate $K_8[Nb_6O_{19}].14H_2O$ was prepared according to the literature method.[1] The purity of POM salt was confirmed by FTIR and Raman.

Methods

UV-Vis spectra were recorded using an HP 8452A spectrophotometer equipped with a diode-array detector (190−1100 nm range).

pH values were measured using a Thermo SCIENTIFIC, ORION STAR A211 pH meter, or an EUTECH INSTRUMENTS, Cyberscan pH 11 pH/ mv/ °C Meter. Before use, the pH meter was calibrated using standard reference solutions (pH 4.01, 7.00, and 10.01).

Dynamic light scattering (DLS) data were collected at 25 °C using two instruments: 1) an ALV-CGS-8F instrument (ALV-GmbH, Germany). The regular angle of measurements was 90 degrees, the geometry of the detector with respect to the incident beam (otherwise noted), and the CONTIN method was used to obtain hydrodynamic radii (Rh). Before each measurement, solutions were filtered through 0.22 µm polyvinylidene fluoride (PVDF) Millipore filters and diluted accordingly (to minimize the multiple scattering, which might distort the Rh results). 2) Malvern Zetasizer Nano S90, with size measurement from 0.3nm (diameter) to 5 microns using 90-degree scattering optics. Zetasizer software was used to obtain particle diameter. The DLS data were obtained to ensure that colloidal solutions of POM-protected metal-oxide NPs contained significant concentrations of particles in the size range of interest.

Zeta potential measurements were carried out by putting 1.0 mL of solution into the measuring cell occupied with two electrodes. Zeta potential data were obtained using a ZEM 3600, Zetasizer, Malvern Instruments Ltd.

X-ray diffraction (XRD) data were obtained using a Rigaku XtaLAB Synergy-S diffractometer using Cu Kα radiation (λ = 1.5418 Å), operated at 50 kV and 1 mA, equipped with a HyPix-Arc 100º detector.

Fourier-transform infra-red (FTIR) spectra were acquired using a Nicolet Impact 410 spectrophotometer (KBr pellets), analyzed with Omnic 7 software in transmission mode.

X-ray photoelectron spectroscopy (XPS) measurements were performed using an X-ray photoelectron spectrometer ESCALAB 250 ultrahigh vacuum ($1 \cdot 10^{-9}$ bar) apparatus with an Al *Kα* XS4 ray source and a monochromator. The X-ray beam size was 500 μm, and survey spectra

were recorded with a pass energy (PE) of 150 eV, and high-energy resolution spectra were recorded with a pass energy (PE) of 20 eV. Processing of the XPS results was carried out using the XPSPEAK program.

Ultraviolet photoelectron spectroscopy (UPS) measurements were performed on the same ESCALAB 250 system using a He I discharge lamp ($h\nu = 21.22$ eV) as the excitation source. The secondary electron cutoff and valence band regions were recorded to determine the work function and valence band maximum of the samples. During secondary electron cutoff measurements, a negative sample bias (typically -5 to -10 V) was applied to improve the resolution of the low kinetic energy cutoff region. The work function (Φ) was calculated using:

$$\Phi = h\nu - E_{\text{cutoff}}$$

where $h\nu = 21.22$eV and E_{cutoff} is the secondary electron cutoff energy obtained from linear extrapolation of the cutoff edge. The valence band maximum was determined from the extrapolation of the leading edge of the valence band spectrum. The conduction band position was subsequently estimated using the optical band gap obtained from UV-vis absorption measurements.

Samples for high-resolution TEM (HR-TEM), and scanning transmission electron microscopy (STEM) were prepared by pipetting 5-10 µL of the aqueous sample solution onto Au grids covered with thin carbon-support films and dried in air. High-resolution transmission electron microscopy images and analytical data were recorded in a double aberration-corrected Themis Z microscope (Thermo Fisher Scientific Electron Microscopy Solutions, Hillsboro, USA) equipped with a high-brightness FEG at an accelerating voltage of 200 kV. High-angle-annular dark-field (HAADF) scanning transmission electron microscopy (STEM) images were recorded with a Fischione Model 3000 detector with a semi-convergence angle of 21 mrad, a probe current of typically 50 pA, and an inner collection angle of 60.0 mrad.

Energy dispersive X-ray spectroscopy (EDX) analysis. EDX hyperspectral data were obtained with a Super-X G2 four-segment SDD detector with a probe semi-convergence angle of 21 mrad, a beam current of approximately 200 pA. The EDS hyperspectral data were quantified with the Velox software (Thermo Fisher Scientific Electron Microscopy Solutions, Hillsboro, USA), through background subtraction and spectrum deconvolution.

Samples for cryogenic TEM (cryo-TEM) were prepared using a fully automated vitrification device ("Leica"). First, 3 µL of the sample solution was placed by pipette onto a glow-discharged 300 Mesh Cu grid covered with a lacey-carbon film, held inside a 100% humidity chamber. The grid was then mechanically "blotted" and immediately plunged into liquid ethane (b.p. 185K) cooled by liquid nitrogen (b.p. 77K). Data were collected on the FEI Tecnai 12 G2 instrument (120 kV) and the Gatan slow-scan camera, 3 using a low-dose regime (to slow down the crystallization of vitrified water and to delay the formation of other artifacts due to beam damage). All images from both dry- and cryo-TEM (including electron diffraction patterns) were analyzed using Digital Micrograph Gatan Inc. software.

Inductively coupled plasma optical emission spectroscopy (ICP-OES) was performed using a SPECTRO ACROS ICP-OES analyzer calibrated using standard solutions of the respective elements.

Isothermal titration calorimetry (ITC) was performed on a NANO ITC System (TA Instruments). Titrations were performed in pure water as a reference at pH 7. Injections of 5 µl of 4.4 mM $Cu(NO_3)_2$ were added from a computer-controlled microsyringe at an interval of 300s into a 1.47 µM complex solution of **1** and **2**, with stirring at 300 r.p.m. at 25 °C. The experimental data were analyzed with NanoAnalyze software (TA Instruments) and were fitted to an independent model concurrently against a blank (addition of $Cu(NO_3)_2$ to water) constant model to adjust for the heat of dilution.

Mott-Schottky analysis of the **1** was performed using a CHI 760C potentiostat in a three-electrode cell at 25 ± 2 °C. A 2-mm diameter glassy carbon electrode served as the working electrode, while a Pt-wire and an Ag/AgCl (3M KCl) electrode were used as the counter and reference electrodes, respectively.

Analysis of gaseous product. Gas chromatography (GC) data were obtained using an Agilent 8860 GC equipped with a thermal conductivity detector (TCD) under the condition of Ar used as the carrier gas, oven temperature of 80 °C, and flow rate of 12 mL/min.

Synthesis of Hexaniobate-complexed NC 1. The hexaniobate-complexed NCs were synthesized by a simple hydrothermal method. In a typical synthesis, $SnCl_4.5H_2O$ (0.195 g, 8 mM) was dissolved in 48.7 mL of deionized water and stirred for 10 minutes, giving a colorless solution (pH ~1). The pH of the solution was raised to ~12 using freshly prepared 1 M KOH. After that, a hexaniobate ligand, 14 mL solution of 20 mM $K_8Nb_6O_{19}.16H_2O$ (0.4 g), was added to the solution, yielding a colorless solution. The solution was stirred (900 rpm) at room temperature for 1 hour to ensure complete hydrolysis of tin (IV) ions. The NC **1** was obtained as a clear, colorless solution after 20 hours of hydrothermal reaction in an 85 mL Teflon-lined 316 stainless-steel reaction vessel at 120 °C.

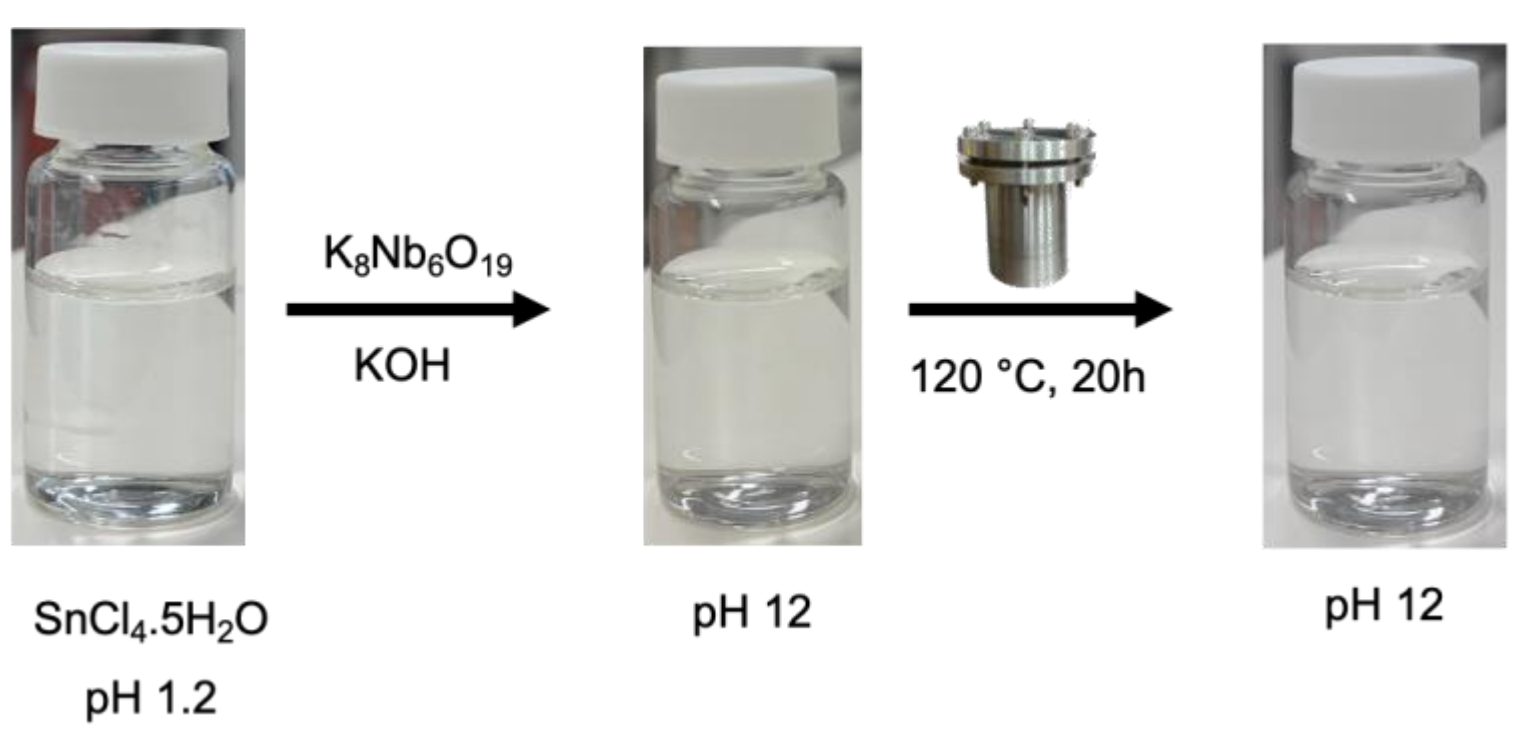

Fig. S1. Synthetic scheme for preparation of 3.2 nm hexaniobate-complexed SnO_2 NCs (**1**).

Synthesis of bulk SnO_2 nanoparticles. The bulk SnO_2 nanoparticle was synthesized under similar conditions to NC **1**, except that hexaniobate was not used as the protecting ligand.

Synthesis of hexaniobate-complexed CuO NCs protected Au NPs. Firstly, the hexaniobate-complexed CuO NC was prepared by following the published method.[2] After that, a 1.5 mL

solution of synthesized CuO NC (4 μM in water, pH = 10.0 ± 0.1, adjusted with CsOH from an initial pH of 7.5) was added, under stirring to a vial containing 1.5 mL of Cs-citrate-protected Au NPs (0.5 mM Au, 6.2 × 10^{-9} M Au NPs with an average diameter of 14 nm, at pH 9.0 ± 0.1). The resulting mixture was left undisturbed for 20 hours, during which the pH stabilized at 9.7 ± 0.2. The solution-state structure of the assembly of hexaniobate-complexed CuO NC on 14 nm Au NP was confirmed via cryogenic transmission electron microscopy (cryo-TEM), which visually validated the monolayer structure (Fig. S26).

Isolation and purification of 1. The nano-sized hexaniobate-complexed SnO_2 NC **1** was first precipitated by the addition of 1 M KCl. Under these conditions, *reversible* aggregation of **1** decreases their solubility in water, so that they can be separated from the supernatant solution by centrifugation (30 min at 6000 rpm). Notably, millimolar concentrations of the primary hexaniobate byproducts and unreacted $SnCl_4$ are fully soluble in 1 M KCl and remain in the supernatant solution. After decanting the supernatant solution by pipette, **1**, a hydrated white solid, was collected and dissolved in 20 mL of pure water, to give a clear, colorless solution. Two additional purification cycles of precipitation by the addition of KCl, followed by centrifugation and re-dissolution in pure water, were carried out to be certain that trace amounts of byproducts were no longer present. Any remaining traces of byproducts in the solution, along with small amounts of KCl precipitated with the nanocrystals, were removed by dialysis for 24 h against deionized water (1 L). This involved placing the solution in a cellulose membrane in a 1 L beaker, during which time, the water outside the dialysis membrane was replaced every 8 hours.

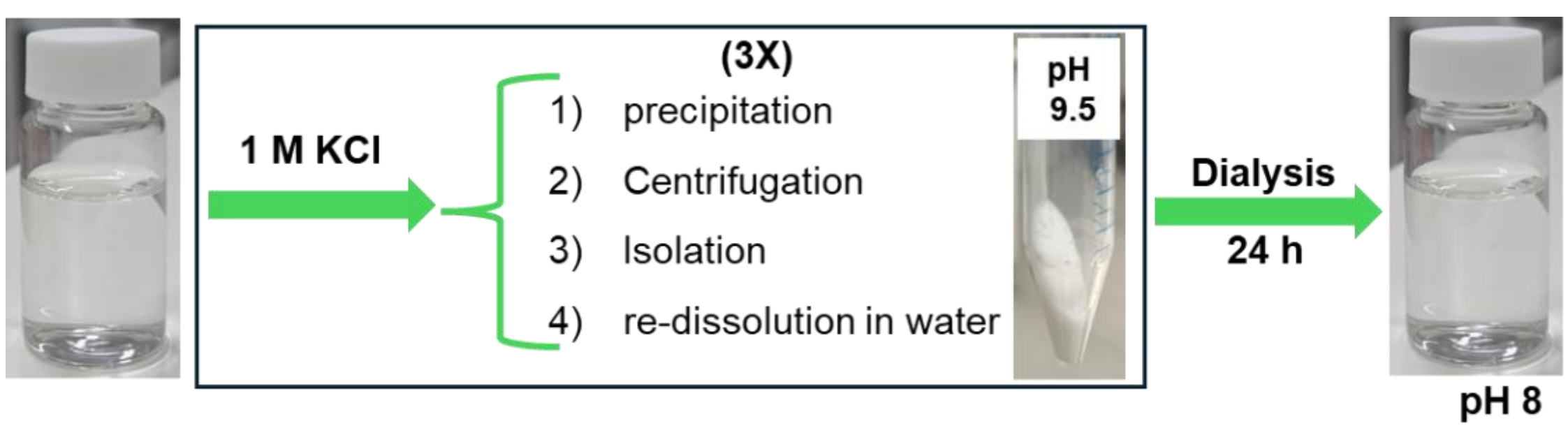

Fig. S2. Purification of **1** by precipitation, centrifugation, isolation, and redissolution in water followed by dialysis. **1** is precipitated by the addition of 1 M KCl (LiCl and NaCl can also do the same work) and centrifuged to get a pellet of hydrated salt of **1** (shown in the Falcon tube), which can be redissolved in pure water to get a clear, transparent solution of **1**. This process was repeated three times and then dialyzed for 24 hours, during which the water in the beaker was changed twice.

Characterization of NC (1). SnO_2 core was characterized by routine microscopic tools in the solution and solid-state, while the hexaniobate ligands on the surface of the nanocrystals were characterized by a variety of solution and solid-state microscopic and spectroscopic techniques and analytical tools. The characterization of the hexaniobate ligand on the surfaces of colloidal SnO_2 nanocrystals in solution is extremely challenging. Recently, covalently attached POMs on MnO_2,[3] and CuO cores[2] were fully characterized using a variety of solid and solution-state

microscopic, diffraction, and spectroscopic methods, and a similar approach has been used to characterize the hexaniobate ligand on the surface of SnO_2 also. The ICP-OES of a purified solution of **1** revealed a Sn: Nb ratio of 61:39.

Preparation of 1 and 2 FTO films. Functionalization of FTO films with **1** was carried out by dropping 100 µL of a 53 µM (0.0053 µmols, 0.75 mg) NC**1** solution onto the FTO surface and drying at room temperature for 6 h. A similar method was used to obtain **2** on FTO. The irradiated area was 1 cm^2.

Photocatalytic Cu^{2+} reduction. Photocatalytic Cu^{2+} reduction experiments were performed in an air-tight quartz cuvette (3.5 mL total volume) containing 3.0 mL of a 1.76 µM solution of NC **1** (0.0053 µmols, 0.75 mg) with 5 vol% isopropanol. The cuvette headspace was evacuated and refilled with high-purity Ar(g) four times to remove atmospheric oxygen, followed by bubbling Ar(g) through the solution for 20 min to eliminate dissolved O_2. The sealed cuvette was then irradiated using a 150 W Xe lamp (USHIO Inc., Japan) with a $\lambda > 305$ nm cut-off filter. The cuvette was positioned 12 cm from the light source. UV-vis absorption spectra were collected at 5 min intervals to monitor the photoreduction of Cu^{2+} to Cu^0.

Photocatalytic water splitting. Photocatalytic water-splitting experiments were conducted in an untreated Pyrex borosilicate glass reaction vessel[4] with a total volume of 17.5 mL, using a 150 W Xe lamp equipped with a 305 nm cut-off filter. A precisely measured aliquot of NC (**1**) was diluted in 8.0 mL of Milli-Q water to obtain a final NC (**1**) concentration of 0.66 µM (0.053 µmols, 0.75 mg). The headspace of the vessel was evacuated and refilled with high-purity Ar(g) four times to remove atmospheric oxygen, followed by bubbling Ar(g) through the solution for 30 min to remove dissolved O_2. The sealed vessel was then irradiated with a 150 W Xe lamp (USHIO Inc., Japan) with a $\lambda > 305$ nm cut-off filter, with the reactor positioned 12 cm from the light source. Gas samples (0.5 mL) from the headspace were withdrawn at 2 h intervals and analyzed using an Agilent gas chromatograph (GC) equipped with a thermal conductivity detector (TCD). Argon was used as the carrier gas, and peak areas were calibrated using air as the external standard (20.9% O_2 in air).

Photocatalytic reduction of CO_2 measurements. Photocatalytic reduction of CO_2 was carried out in an untreated Pyrex borosilicate-glass vessel with a volume of 17.5 mL. 8 mL H_2O was added, and transparent windows of FTO (control) and catalyst-dropcasted FTO were centered in the tube, which was then sealed with a gas-tight rubber septum. For each experiment, the solution was purged for 1 hour with CO_2 (99.999%), regulated by a mass-flow controller, and passed at room temperature through an MeCN bubbler, and the Pyrex reactor was then irradiated with a 150 W Xe lamp (USHIO Inc., Japan) with a $\lambda > 305$ nm cut-off filter. After 4 h, 500 µL of the headspace gases were analyzed by gas chromatography (see above for instrumental details).

Calculation of the apparent quantum yield (AQY). The apparent quantum yields (AQYs) for photochemical Cu(0) formation, photocatalytic water splitting, and photocatalytic CO formation were determined under identical irradiation conditions. A 150 W Xe lamp (USHIO Inc., Japan) equipped with a 280 nm band-pass filter was used to irradiate the samples, providing monochromatic light at $\lambda = 280$ nm. The incident light intensity was measured using a 1928-C

Optical Power Meter (Newport Corp.) equipped with a 919P-250-35 thermopile sensor. The measured incident light intensity at sample was 1.8 mW cm^{-2}, with an illuminated area of 1 cm^2.The energy of each photon of 280 nm light is 7.1 x 10^{-19} J/photon (E of photon = hc/λ; where h is Planck's constant, c is the speed of light, and λ is the wavelength of light).

Because each Cu (0) formation from Cu^{2+} requires two electrons, and therefore two photons, the apparent quantum yield for Cu (0) formation was determined by the following equation:

AQY = 2· (rate of Cu (0) formation, mol. s^{-1}) / incident photon per unit time (Einstein.s^{-1}).

Intensity of incident light (W cm^{-2})	0.0018
Illumination area (cm^2)	1
Energy of a photon (J)	7.1 * 10^{-19}
Energy of the photons (Einstein s^{-1})	4.2 * 10^{-9}
Cu (0) formation (mol s^{-1})	6.38 * 10^{-11}
$AQY_{Cu(0)}$	0.0304

For overall photocatalytic water splitting, each oxygen molecule production requires four electrons, and therefore four photons, AQY for oxygen production was determined by the following equation:

AQY = 4*(rate of O_2 production, mol·s^{-1}) / incident photon per unit time (Einstein·s^{-1})

Intensity of incident light (W cm^{-2})	0.0018
Illumination area (cm^2)	1
Energy of a photon (J)	7.1 * 10^{-19}
Energy of the photons (Einstein s^{-1})	4.2 * 10^{-9}
O_2 production (mol s^{-1})	1.8 * 10^{-11}
$AQY_{water\ splitting}$	0.0171

For photocatalytic CO formation, each CO formation from CO_2 requires two electrons, and therefore two photons, the AQY for CO formation was determined by the following equation:

AQY = 2· (rate of CO formation, mol. s^{-1}) / incident photon per unit time (Einstein.s^{-1}).

Intensity of incident light (W cm^{-2})	0.0018
Illumination area (cm^2)	1
Energy of a photon (J)	7.1 * 10^{-19}
Energy of the photons (Einstein s^{-1})	4.2 * 10^{-9}
CO formation (mol s^{-1})	5.01 * 10^{-11}
AQY_{CO}	0.0238

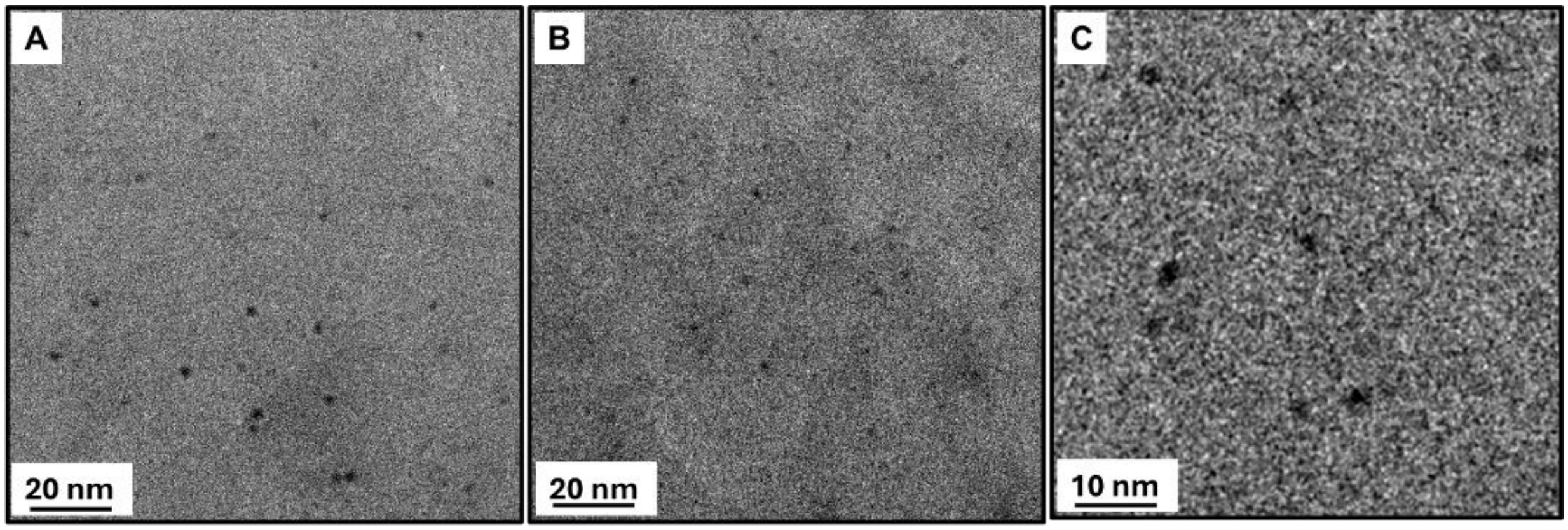

Fig. S3. (A-C) Cryo-TEM images of **1.**

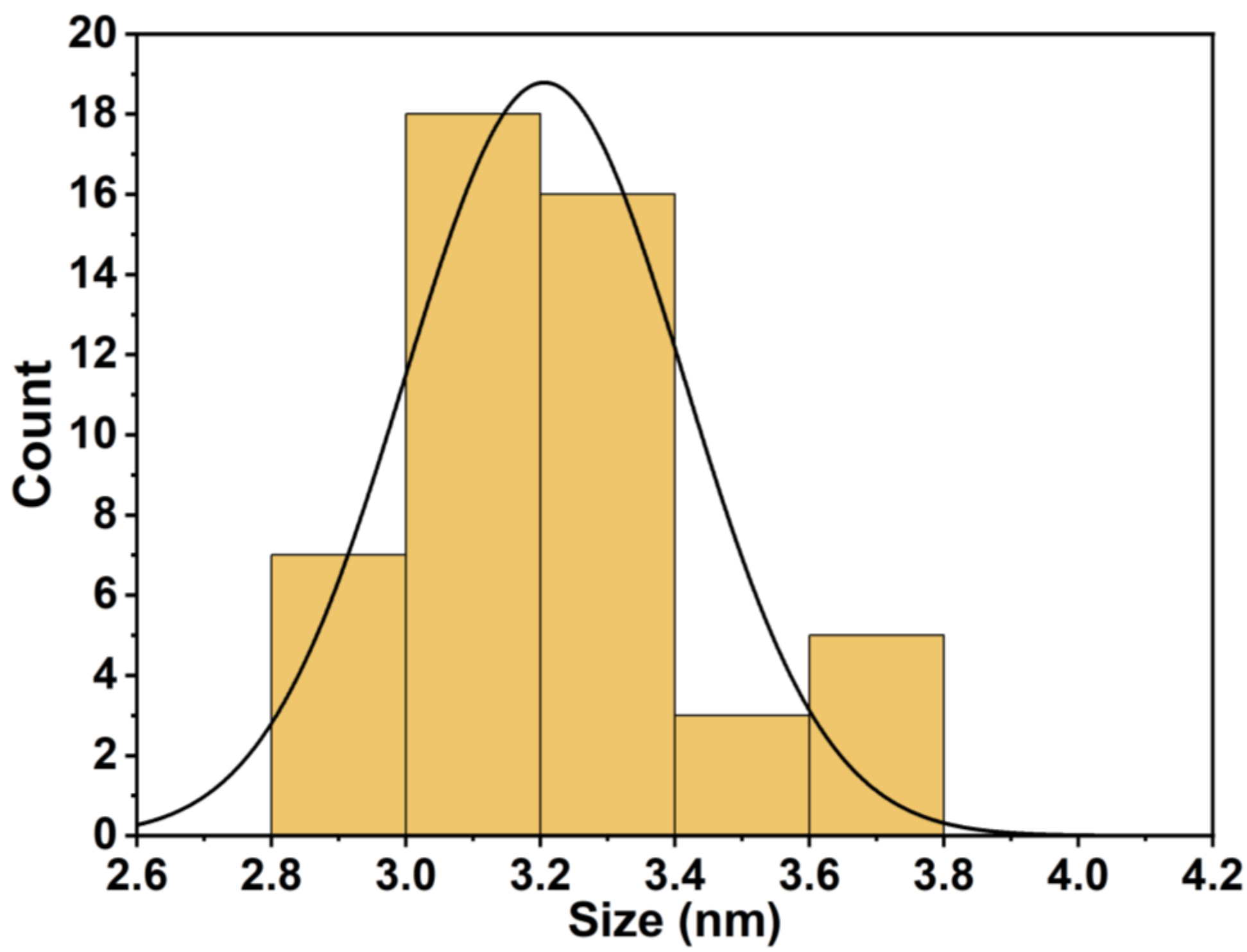

Fig. S4. Histogram obtained from cryo-TEM images of **1,** giving the average diameter of 3.2 ± 0.2 nm.

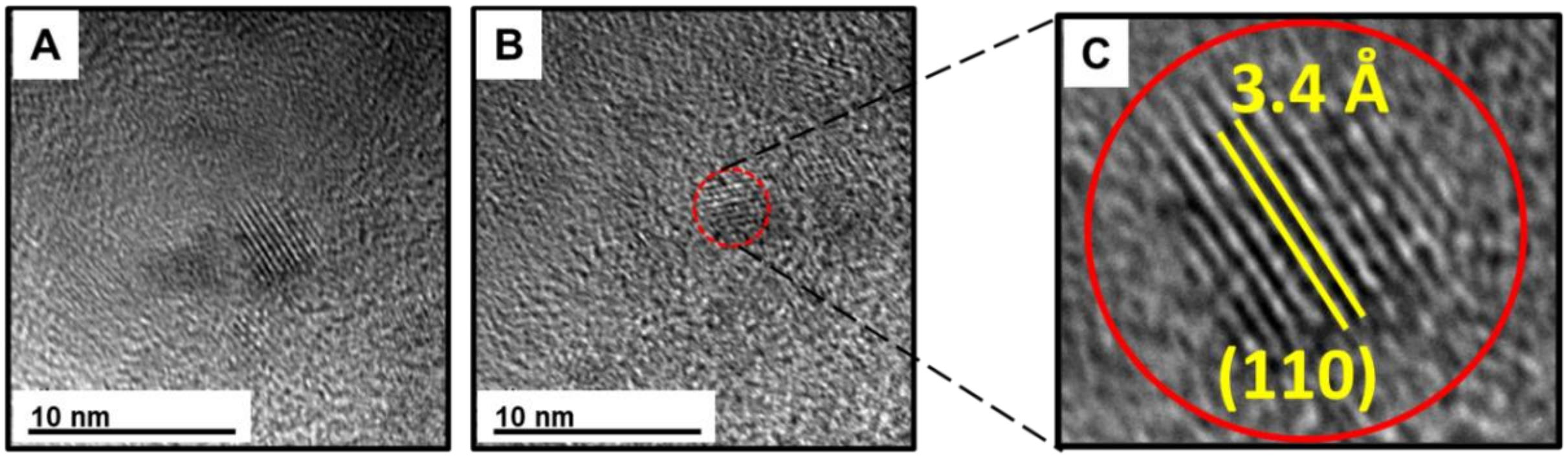

Fig. S5. (A and B) High-resolution TEM images of **1** at different areas. (C) Enlarged view showing lattice fringes with an interplanar spacing of **3.4 Å**, indexed to the **(110)** planes of the rutile-SnO_2 core.

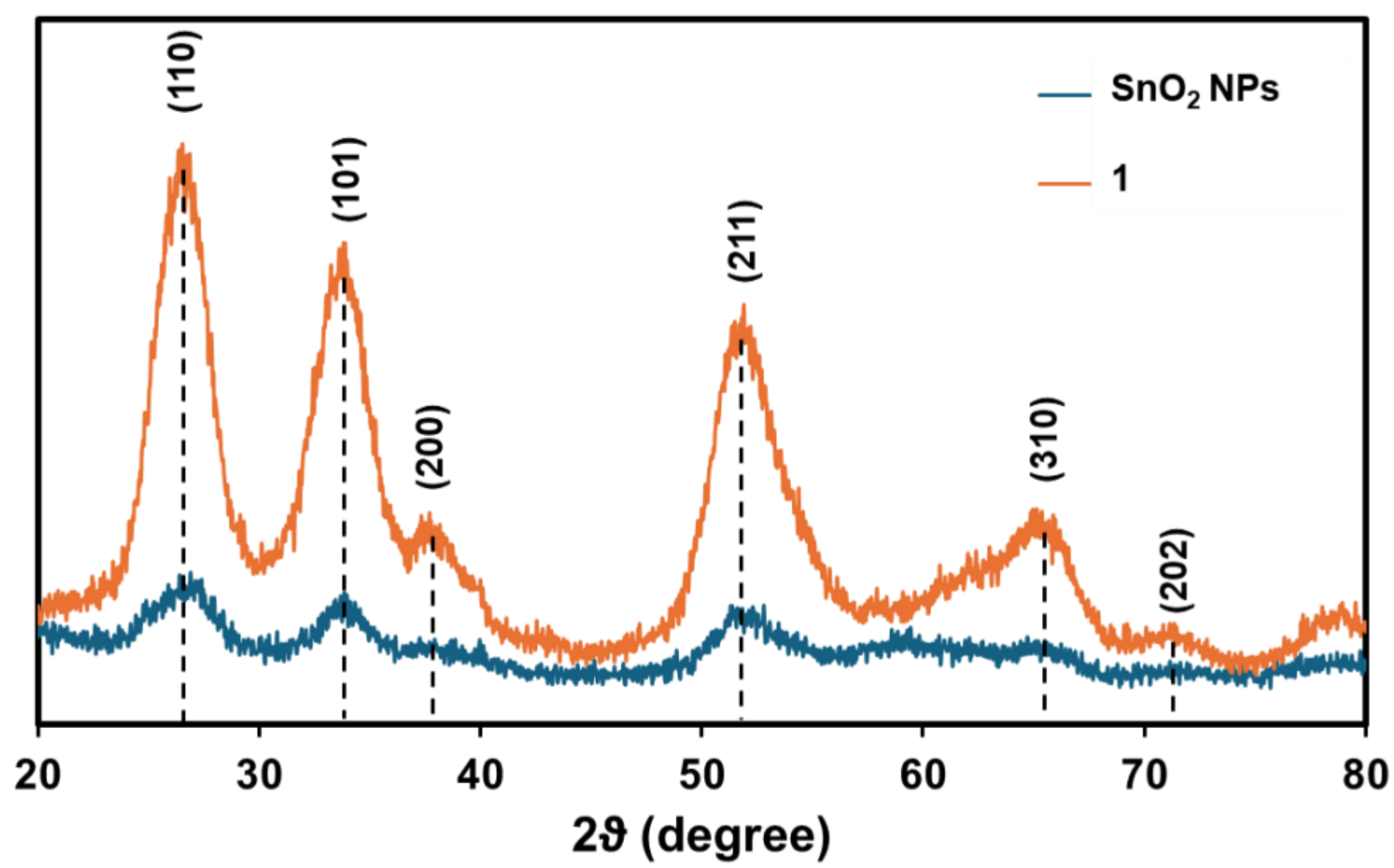

Fig. S6. Powder X-ray diffraction (PXRD) patterns of **1** (orange) and bare SnO_2 nanoparticles (blue), synthesized under similar conditions as **1** except adding hexaniobate. PXRD patterns of both the samples matched well with the characteristic reflections of tetragonal rutile SnO_2, indicating preservation of the crystalline SnO_2 core.

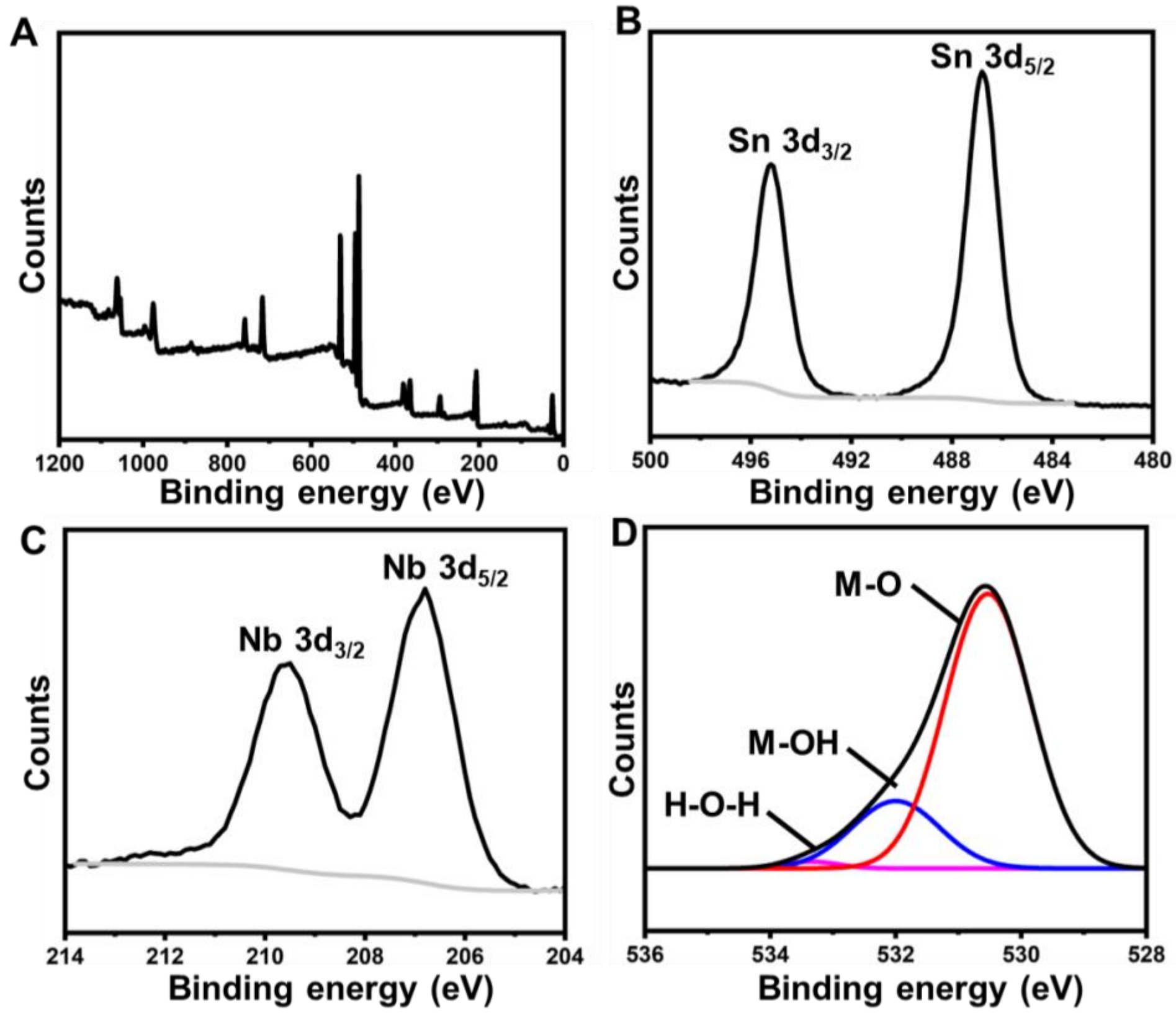

Fig. S7. High-resolution X-ray photoelectron spectra (XPS) of a purified and dried sample of **1** show the presence of Sn, Nb, and O atoms. (A) survey spectrum, (B) Sn 3d core-level spectrum confirming Sn(IV) in the sample, (C) a well-resolved Nb (V) peak in the spectrum further confirms the presence of hexaniobate POM on the SnO_2 surface, and (D) O 1s spectrum suggests the presence of O^{2-}, O-H, and H-O-H. The peak for O-H can originate from the protonated hexaniobate ligand as well as from the Sn(OH) present on the surface of SnO_2. In panel B, the Sn $3d_{5/2}$ peak of **1** (3.2 nm SnO_2 core), appears at a binding energy of 486.56 ± 0.085 eV (based on measurements of two different samples), which lies at the lower end of the values reported for SnO_2 nanoparticles, which range from 486.6 to 487.0 eV.[5-9] As such, the slightly smaller binding energy associated with the Sn $3d_{5/2}$ peak of **1** (panel B) is consistent with electron donation from hexaniobate to Sn(IV). However, the effect is too small to serve as definitive evidence for electron donation from hexaniobate to surface Sn(IV) atoms. One reason for the small value may be the large number of Sn(IV) atoms within the SnO_2 core, which in the XPS experiment are averaged with changes to the surface Sn atoms.

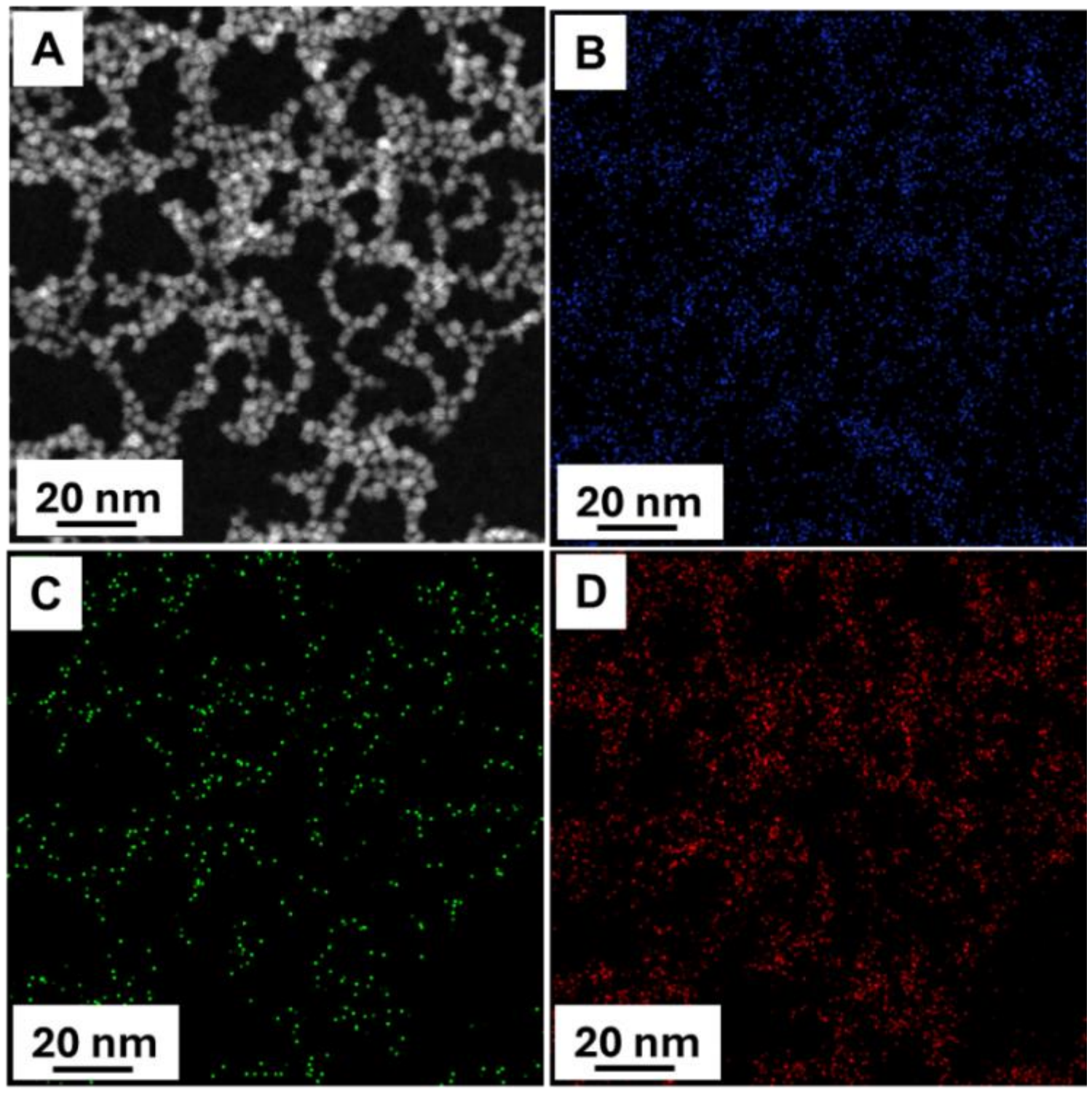

Fig. S8. (A) STEM-HAADF image of **1**, electron mapping from Energy Dispersive X-ray for (B) O atoms (blue), (C) Nb atoms (green), and (D) Sn atoms (red) mapped from image (A), showing the homogeneous distribution of O, Nb, and Sn atoms throughout the NCs.

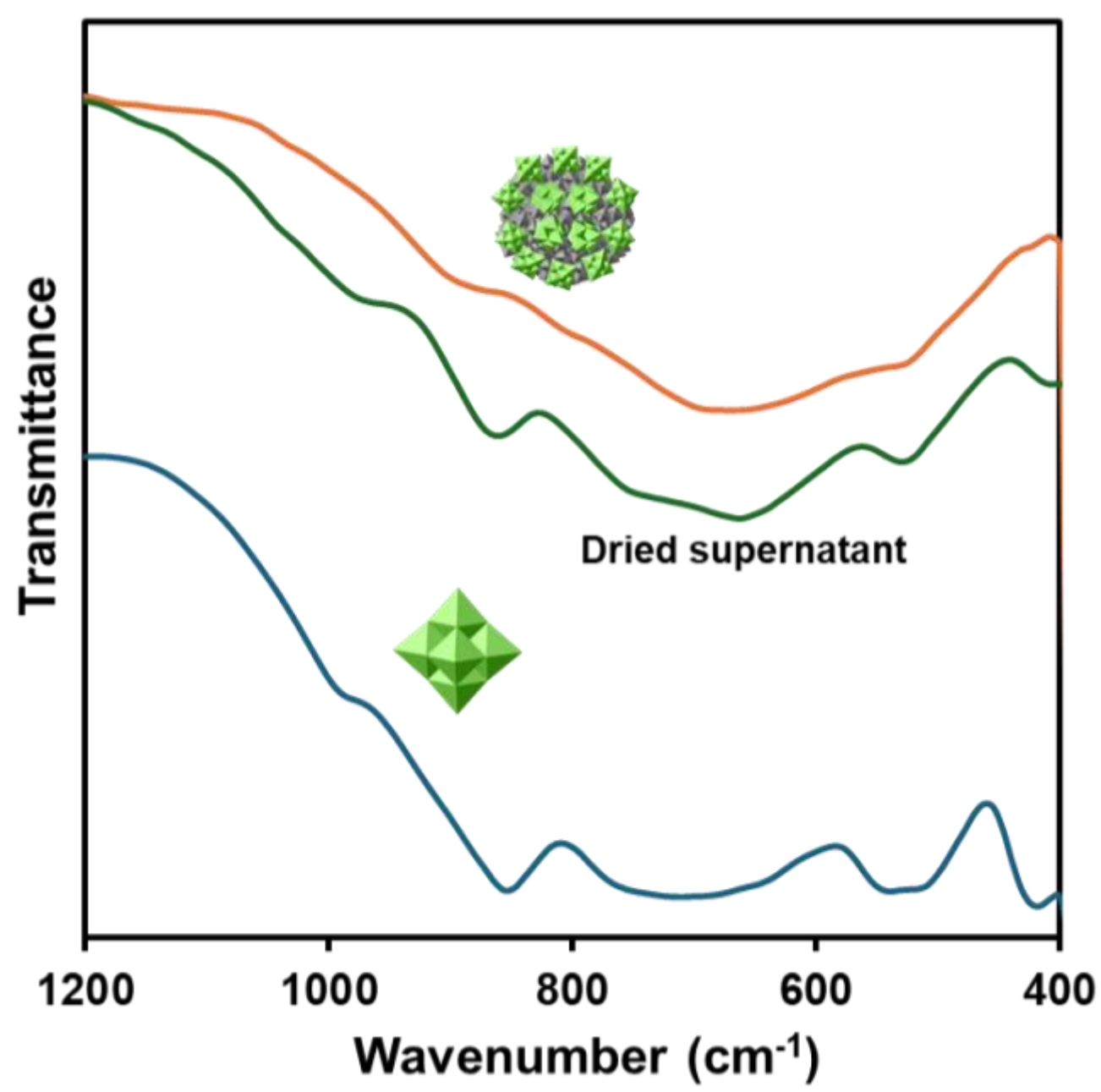

Fig. S9. FTIR spectra of **1** (orange curve), hexaniobate ($K_8Nb_6O_{19}$) as-synthesized ($K_8Nb_6O_{19}$, blue curve), and hexaniobate recovered from the supernatant of **1** (green curve).

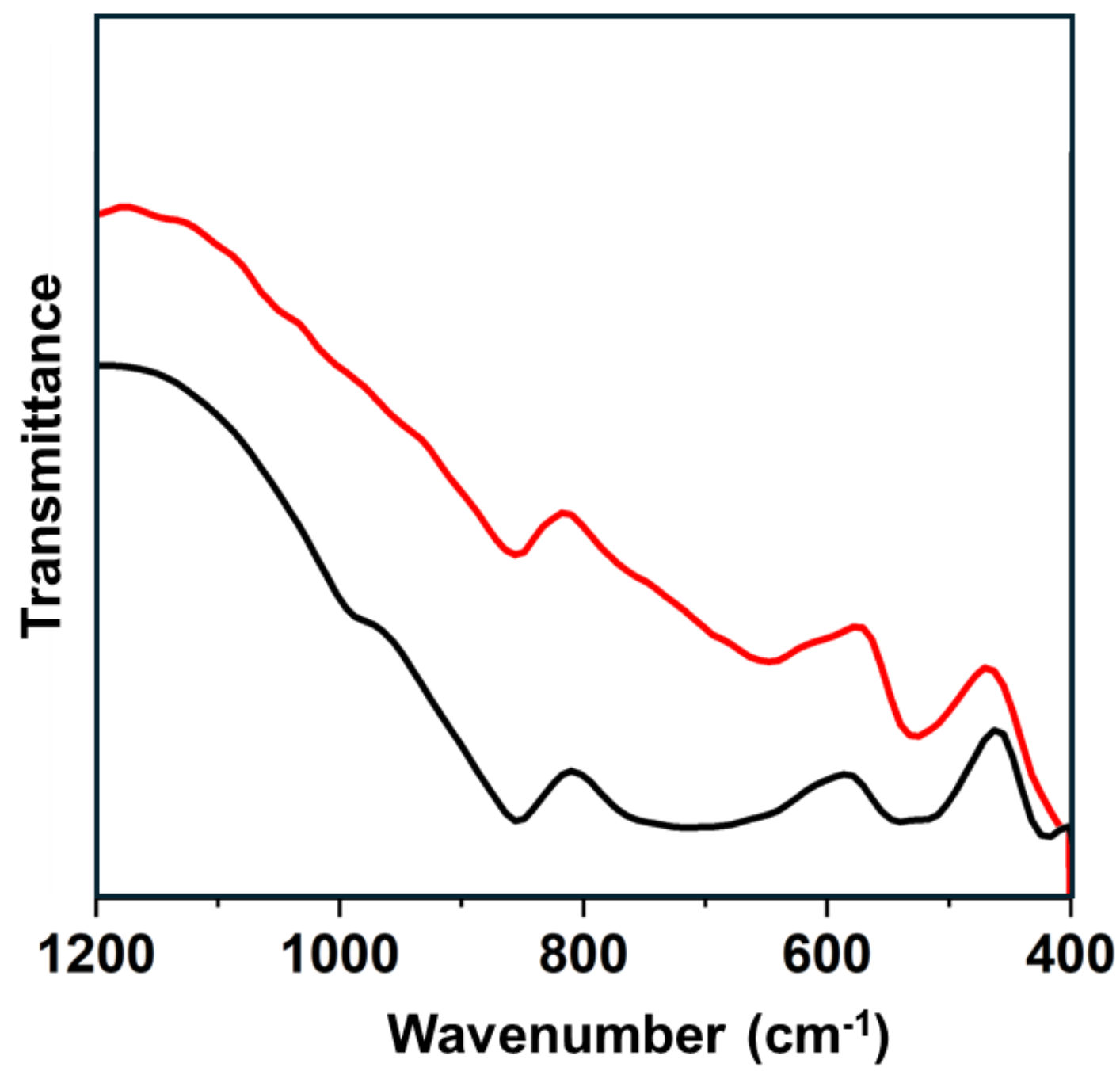

Fig. S10. FTIR spectrum of hexaniobate ($K_8Nb_6O_{19}$, black curve) and the hexaniobate ligand alone heated for 20 hours at 120 °C (red curve). Both spectra match perfectly and further confirm the stability of the hexaniobate ligand under reaction conditions.

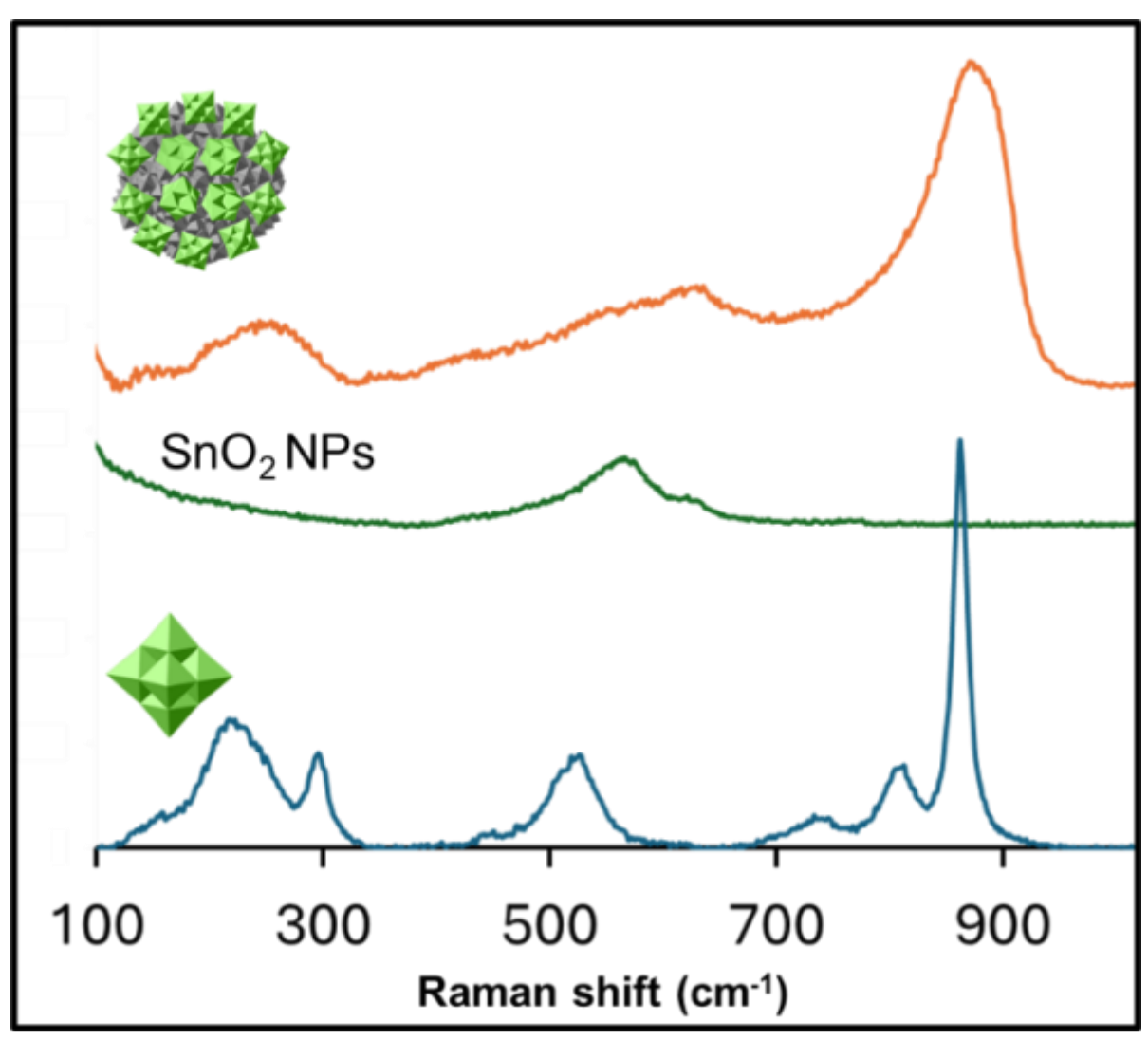

Fig. S11. Raman spectra of hexaniobate ligand ($K_8Nb_6O_{19}$, blue curve), bulk SnO_2 NPs (green curve), and hexaniobate-complexed SnO_2 (**1**, orange curve).

Table S1. Calculation of the number of Sn atoms, the number of hexaniobate ligands, and the atom fractions of Sn and Nb of **1**, considering a 3.2 ± 0.2 nm (diameter) ideal spherical SnO_2 NC core and 0.64 nm^2 POM footprint on the SnO_2 surface.

$[Sn^{IV}]$ (as SnO_2)	0.022 M
Density of SnO_2	6.95 x 10^{-21} g nm^{-3}
Molecular weight of SnO_2	150.71 g mol^{-1}
Volume of SnO_2 solution	0.010 L
Moles of Sn(IV)	2.20 x 10^{-4} mol
Mass of SnO_2 in 0.01 L solution	3.32 x 10^{-2} g
Volume of SnO_2	4.77 x 10^{17} nm^3
Volume of one SnO_2 NC	17.15 nm^3
**Number of Sn atoms per NC**	**480 ± 90**
Number of SnO_2 NCs	2.78 x 10^{17}
$[SnO_2]$ NCs	4.62 x 10^{-5} M
Surface area of one SnO_2 NC	32.15 nm^2
POM footprint on the NC surface	0.64 nm^2
[POM] (bound to NCs)	2.32 mM
**Number of POMs per NC**	**50 ± 0.7**
Sum of Nb + Sn atoms/particle	780
**Atom/atom fraction of Sn**	**61**
**Atom/atom fraction of Nb**	**39**

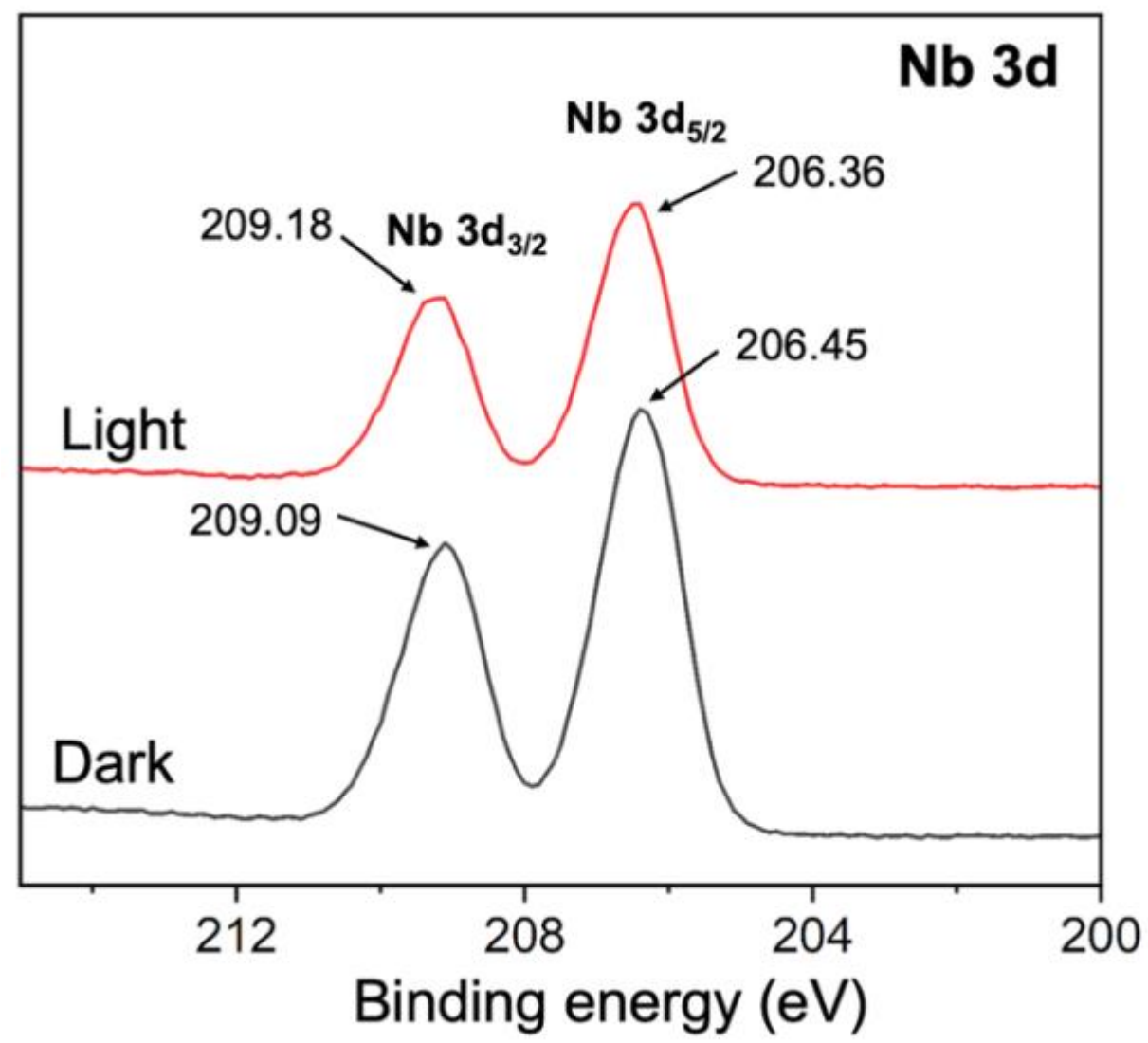

Fig. S12. In situ Nb 3d XPS spectra of hexaniobate recorded (black) in the dark and (red) while irradiated at 280 nm. The effectively unchanged Nb $3d_{5/2}$ and Nb $3d_{3/2}$ peak positions indicate the absence of LMCT excitation at 280 nm. Although it would have been preferable to use a 305 nm LED for the data in Figure 3 of the text, that light source was not available to us. However, the lack of an effect on binding energies upon irradiation of pure hexaniobate at 280 nm is definitively ruling out attribution of the large 0.8 eV decrease in binding energies shown for **1** in Figure 3 of the text to LMCT excitation of the hexaniobate ligands of that complex.

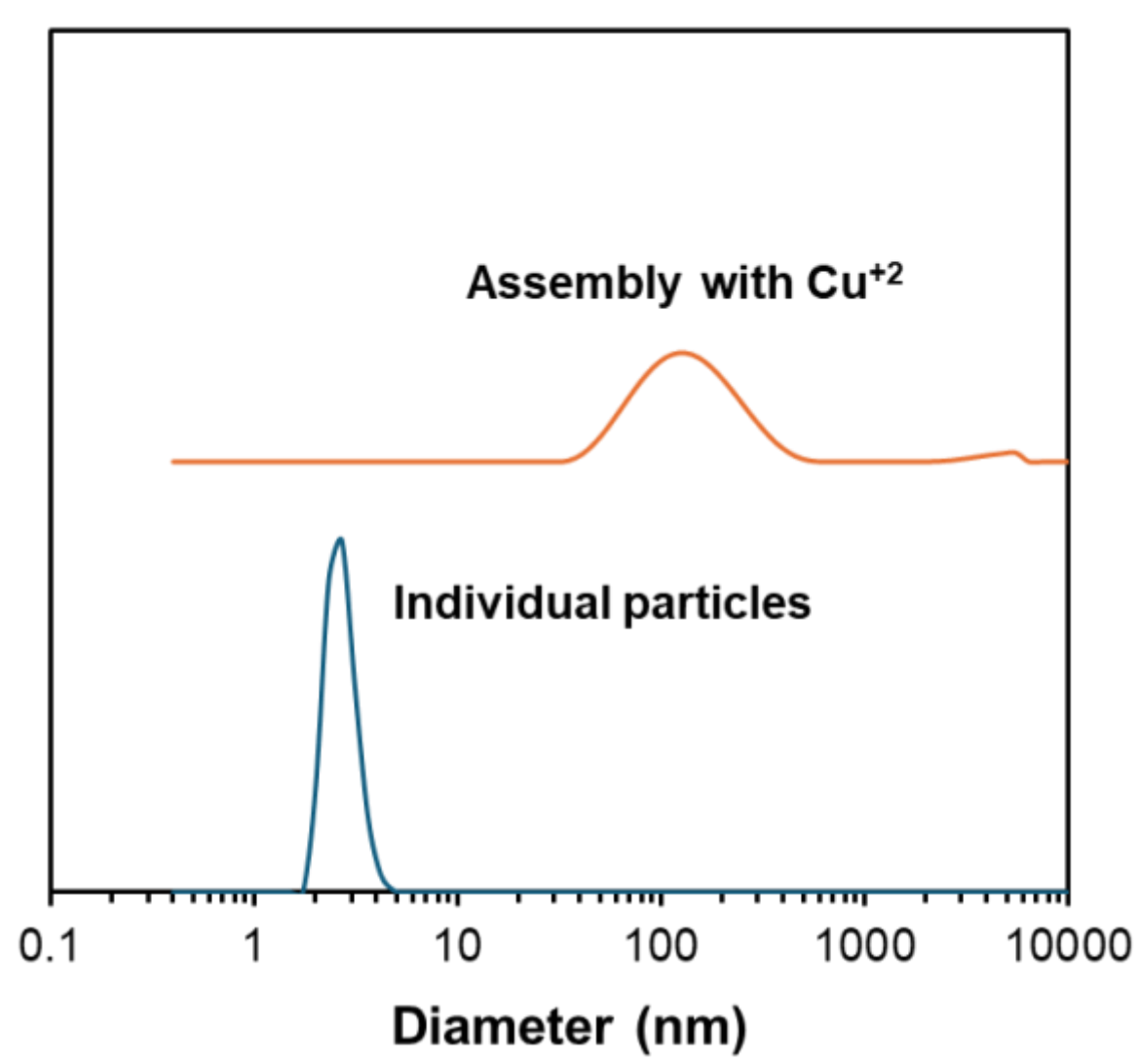

Fig. S13. Dynamic light scattering (DLS) measurements showing individual particles of **1** (blue curve) and assembly of **1** (orange curve) after adding 0.5 mM of Cu^{2+} to a 1.76 µM solution of NCs (**1**).

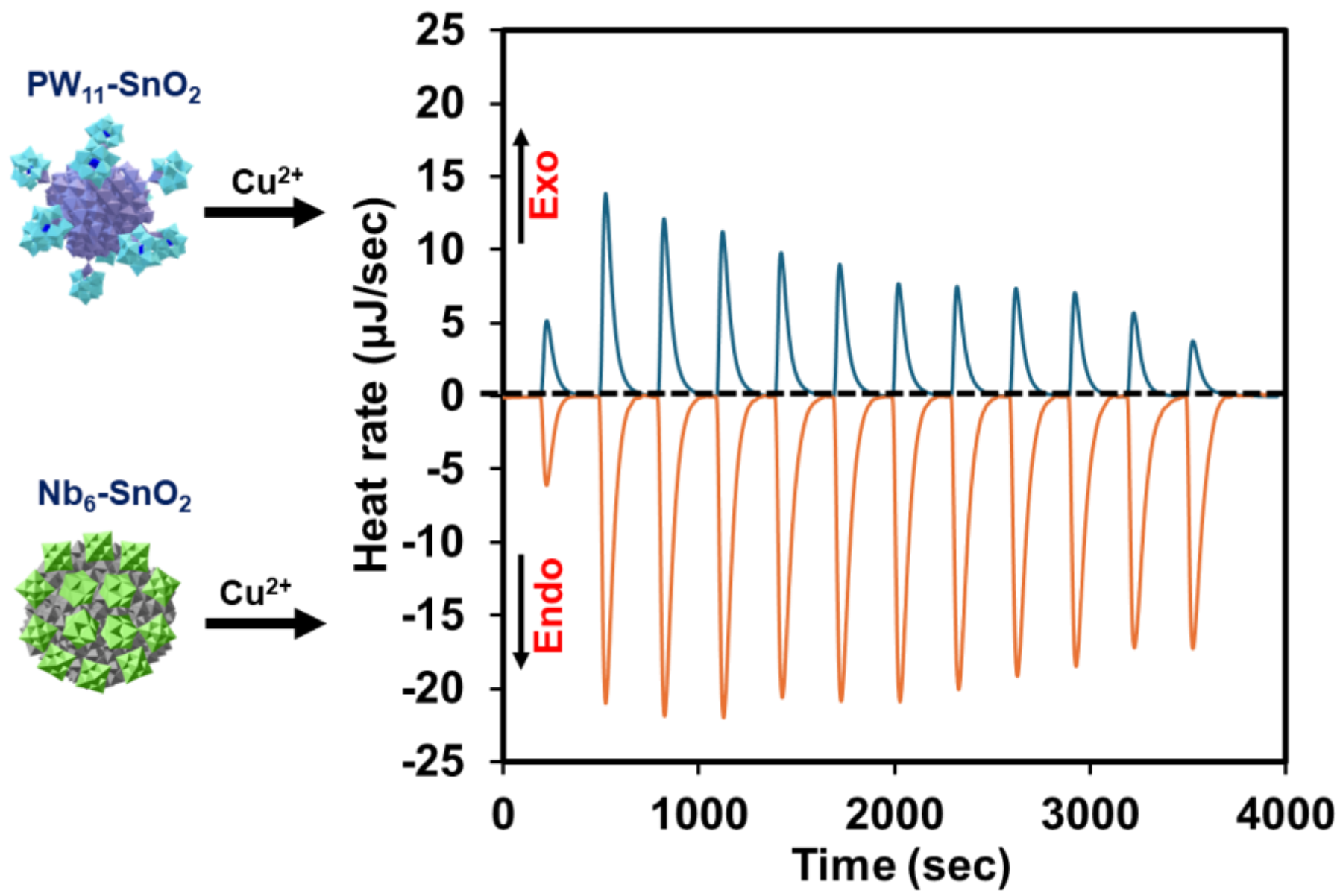

Fig. S14. Isothermal titration calorimetry (ITC) measurements. Titration of Cu^{2+} into an aqueous solution of **2** (top) revealed exothermic signals. Titration of Cu^{2+} into an aqueous solution of **1** (bottom) revealed endothermic signals.

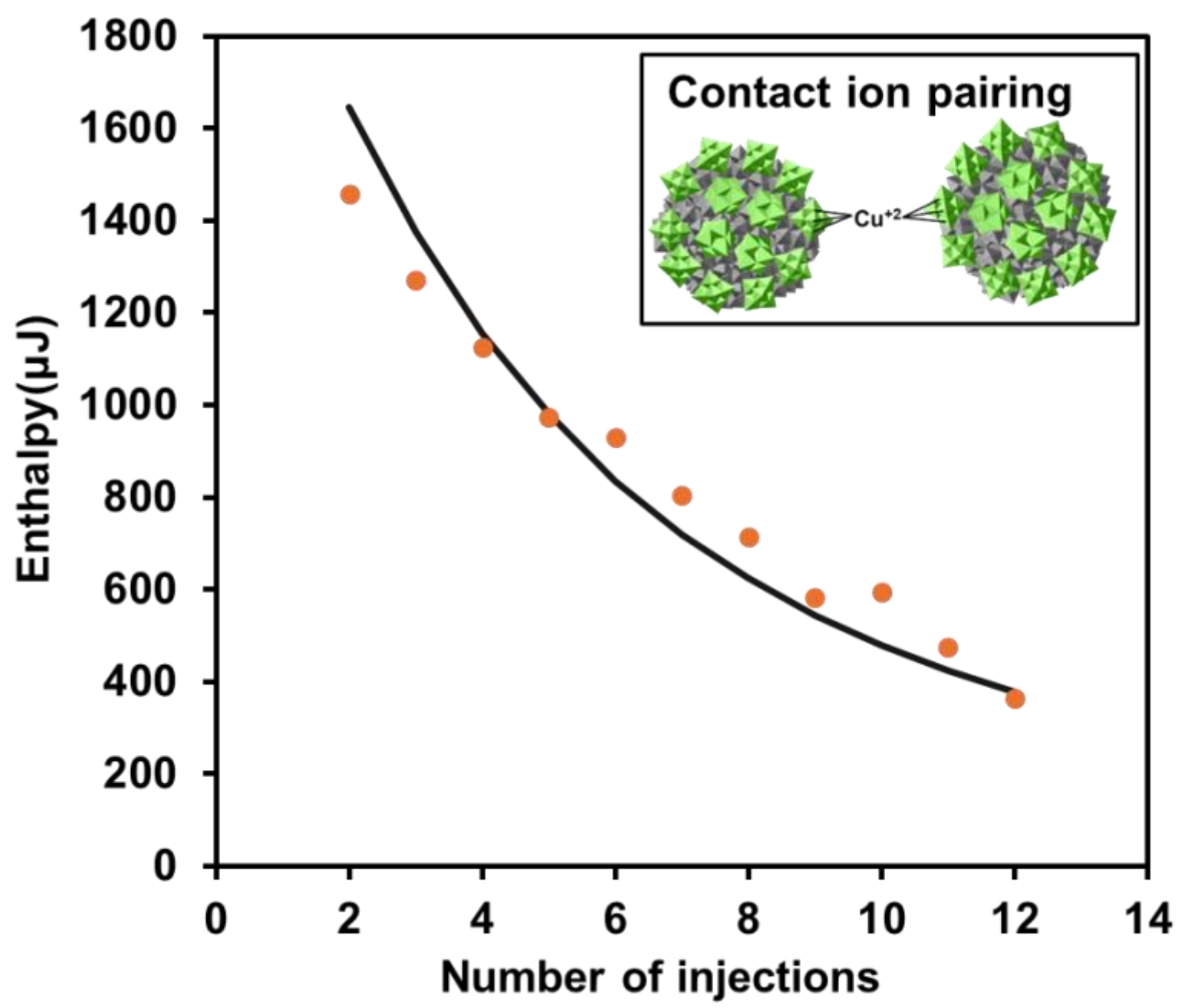

Fig. S15. Orange data points indicate integrated heat from Fig. S14 (bottom) for **1**, while the independent fitting is shown in a solid line. Inset: Direct contact ion pairing of Cu^{2+} with adjacent NCs (**1**).

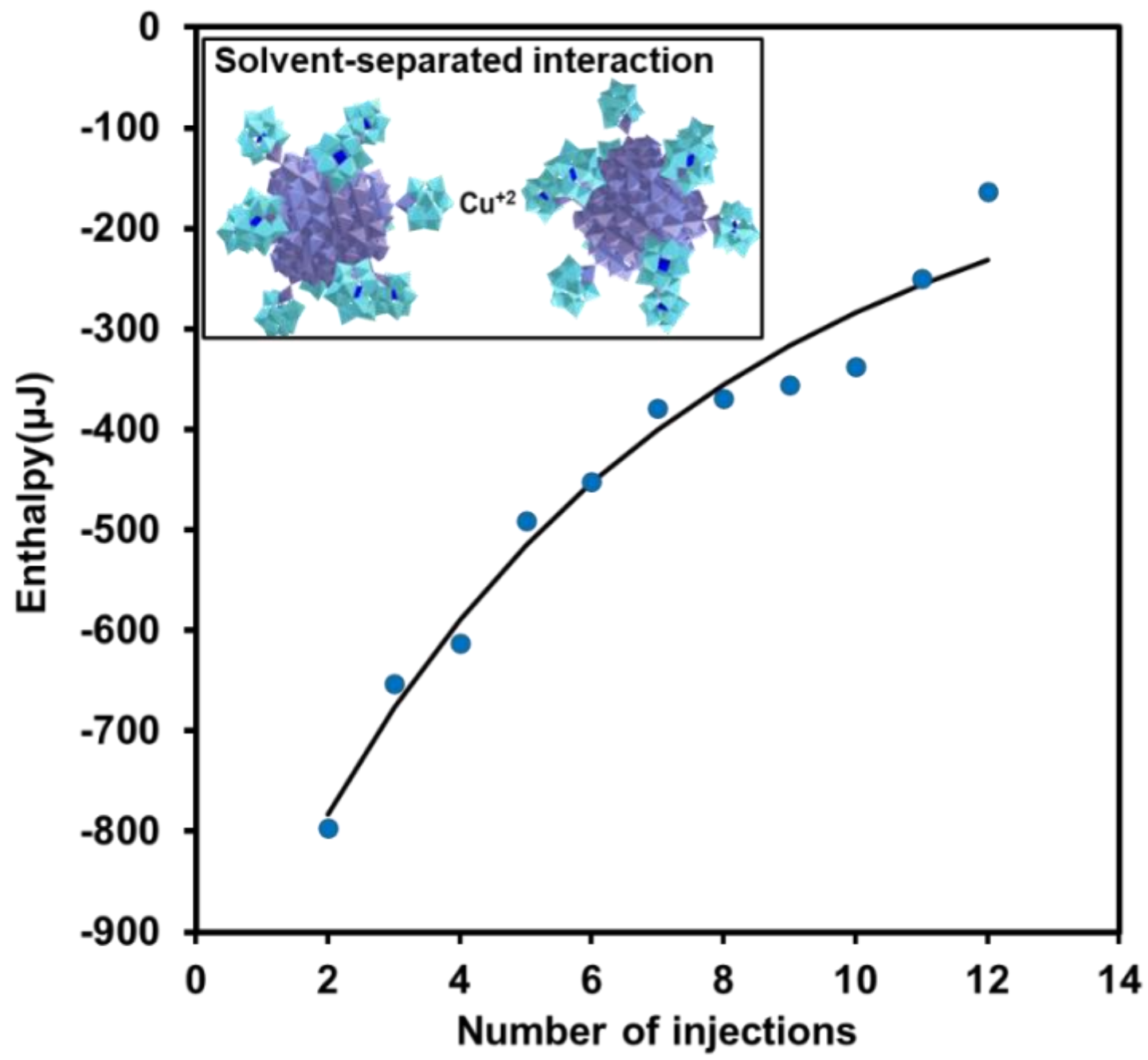

Fig. S16. Blue data points indicate integrated heat from Fig. S14 (top) for **2**, while the independent fitting is shown in a solid line. Inset: Sovent separated interaction of Cu^{2+} with adjacent NCs (**2**).

Table S2. Thermodynamic Parameters from Titrations of Cu^{2+} into Aqueous Solutions of 1 and 2

	ΔH<br>kcal mol^{-1}	ΔS<br>cal $mol^{-1}.K^{-1}$	−TΔS<br>kcal mol^{-1}	ΔG<br>kcal mol^{-1}	K<br>x10^3
**1**	23.56	93.42	-27.85	-4.29	1.44
**2**	-13.42	-31.25	9.31	-4.10	1.04

Parameters were obtained using an independent-site model.

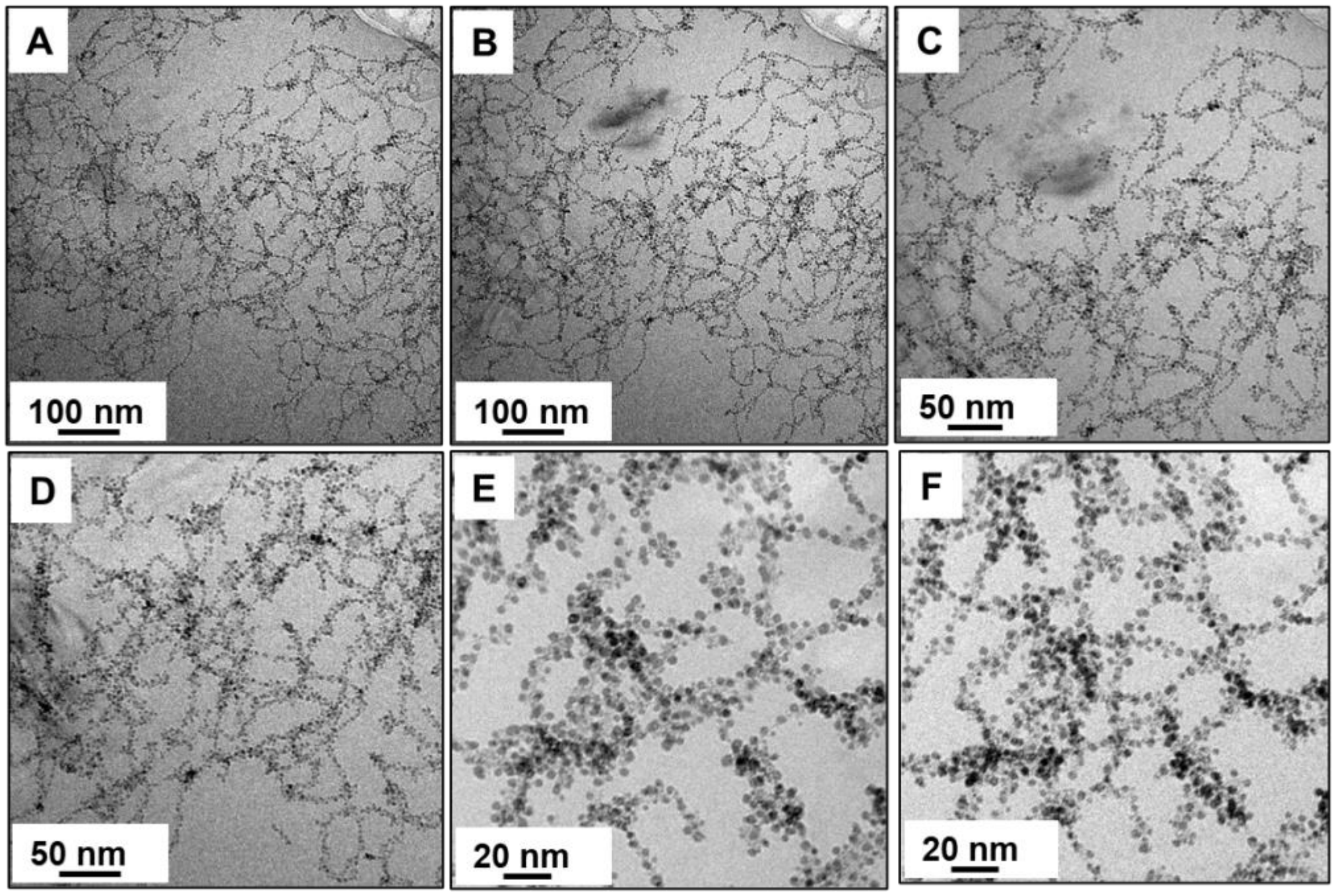

Fig. S17. (A-F) Cryo-TEM images of a solution of NCs (**1**) containing Cu^{2+}**.** Images show 1-D coordination polymer-chain-like nanostructures.

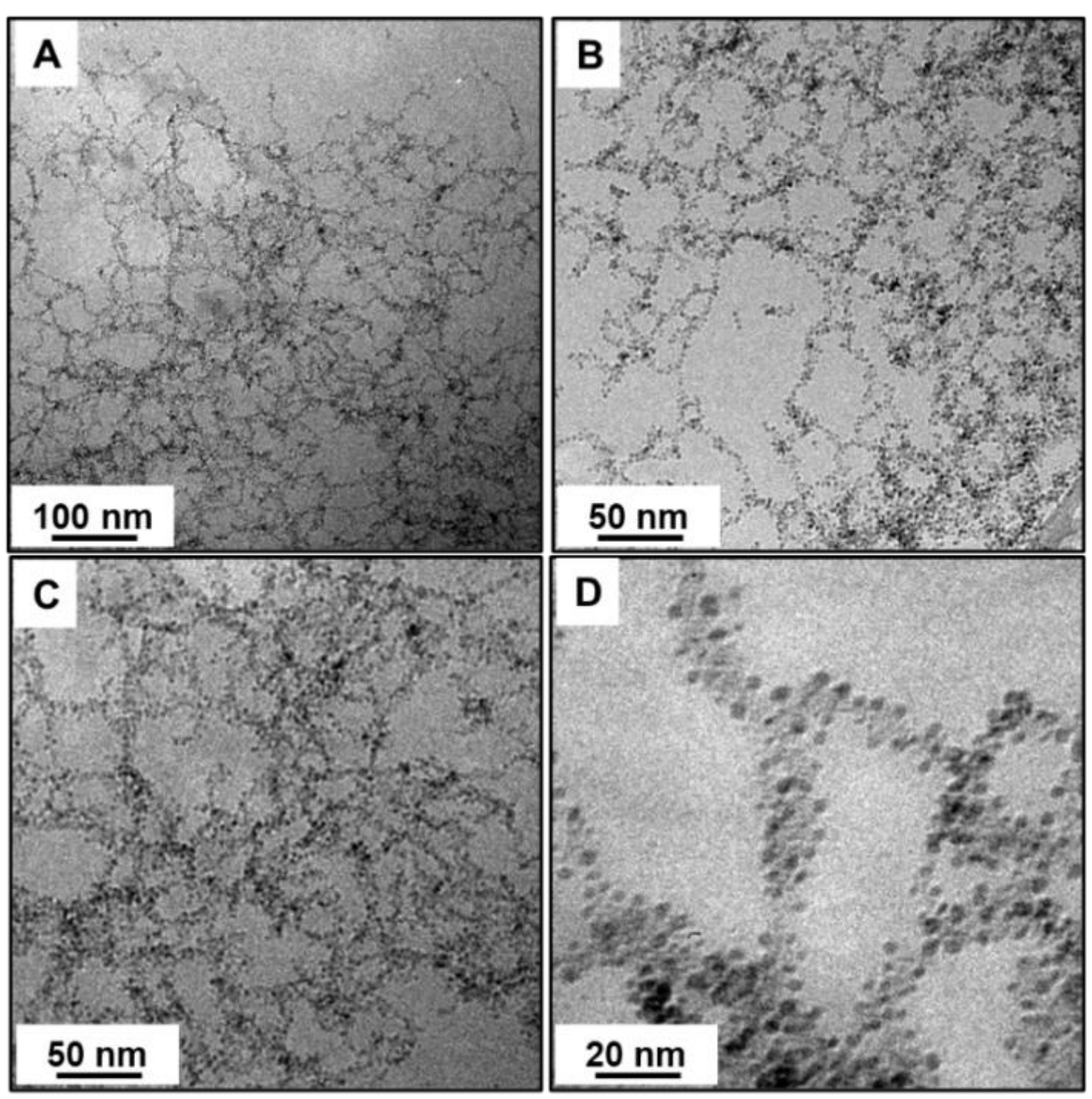

Fig. S18. (A-D) Cryo-TEM images of a solution of NCs (**1**) containing Cu^{2+} after heating at 140 °C for 12 hrs**.** Preservation of 1-D coordination polymer-chain-like nanostructures after heating argues that these assemblies are the thermodynamic product, rather than kinetically trapped structures.

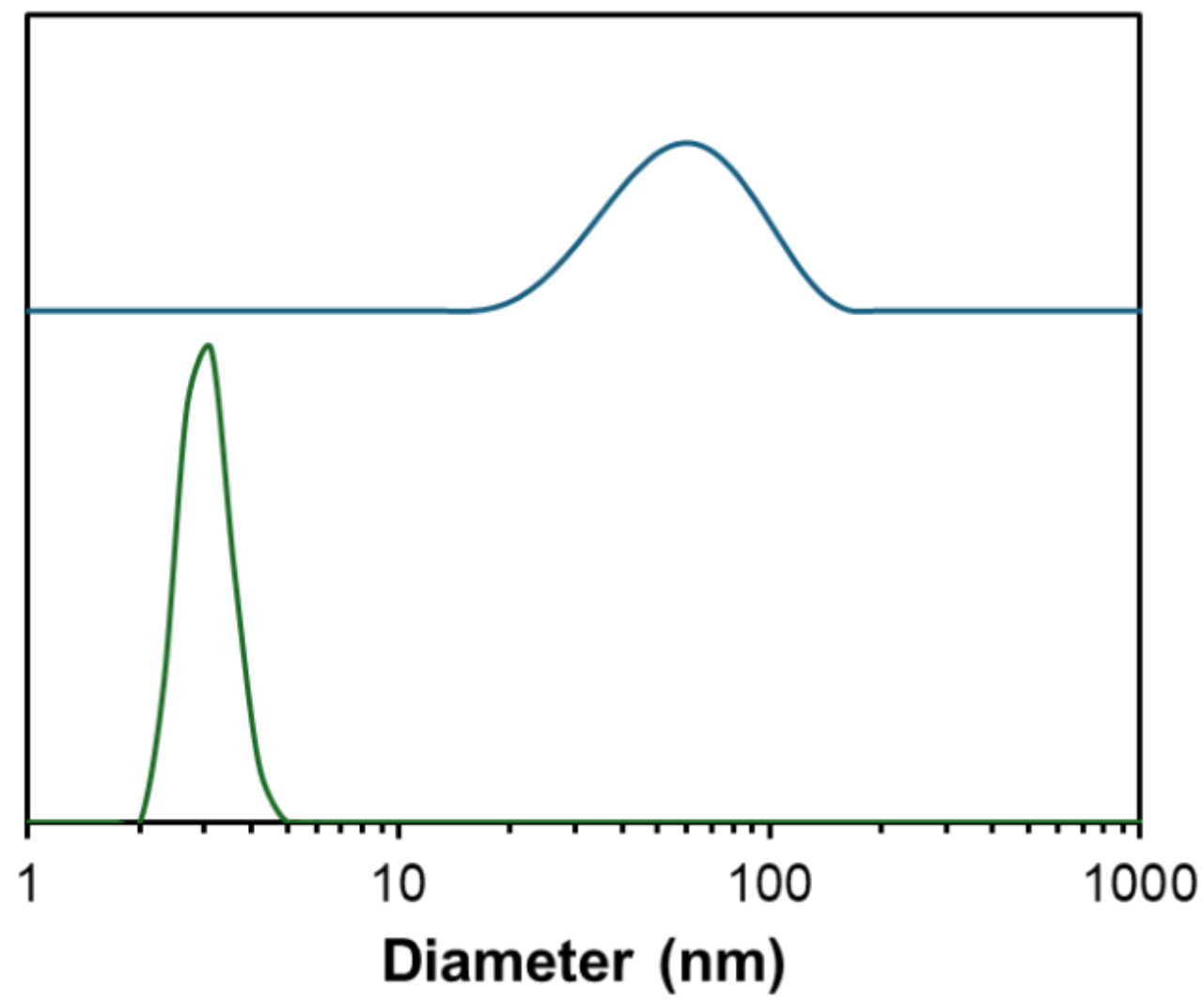

Fig. S19. Dynamic light scattering (DLS) measurements showing individual particles of **2** (green) and assembly of **2** (blue curve) after adding 0.5 mM of Cu^{2+} into the 4 μM solution of **2** NCs.

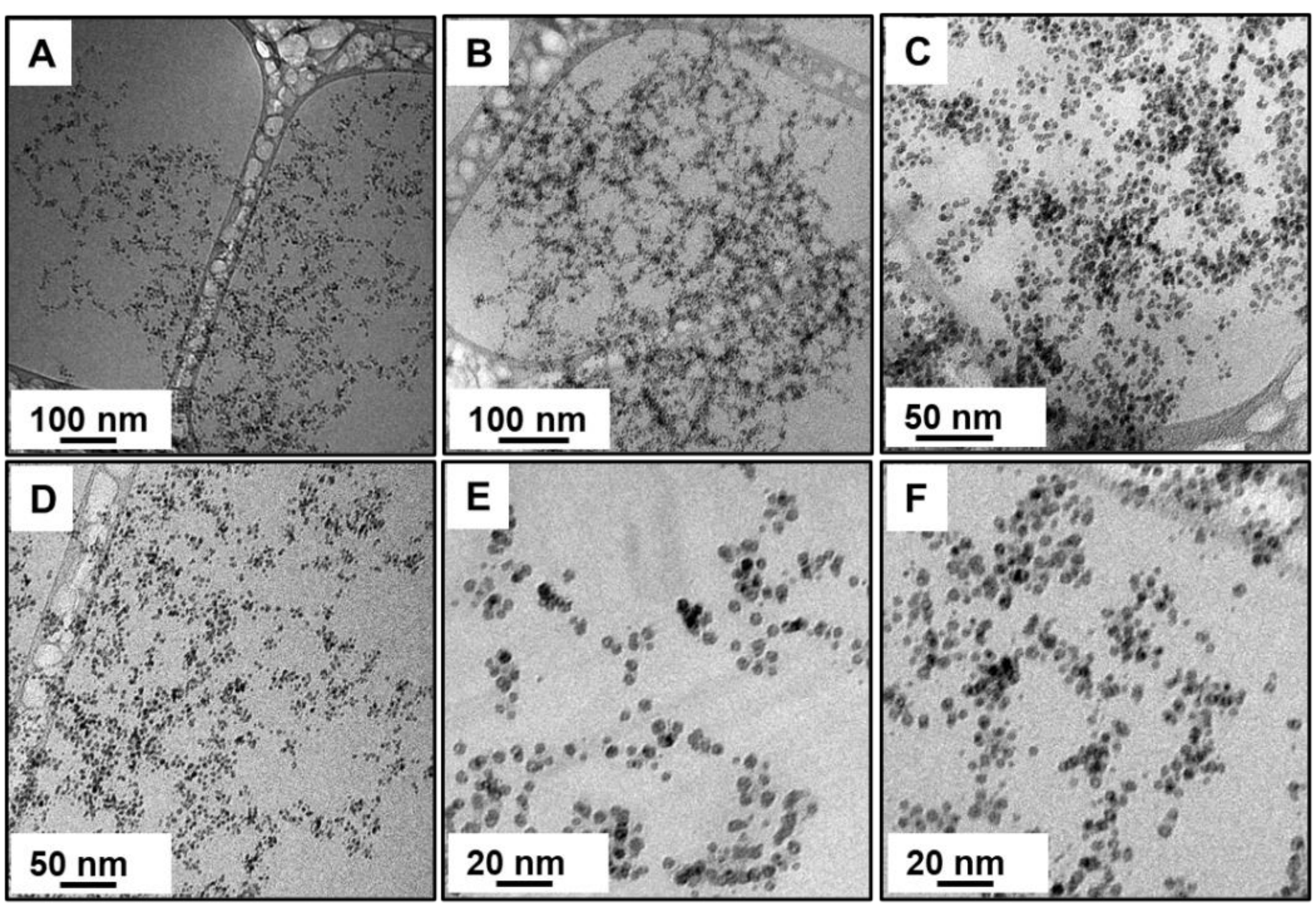

Fig. S20. (A-F) Cryo-TEM images of a solution of **2** containing Cu^{2+}. Images show randomly aggregated **2** particles due to solvent-separated interactions with Cu^{2+} ions.

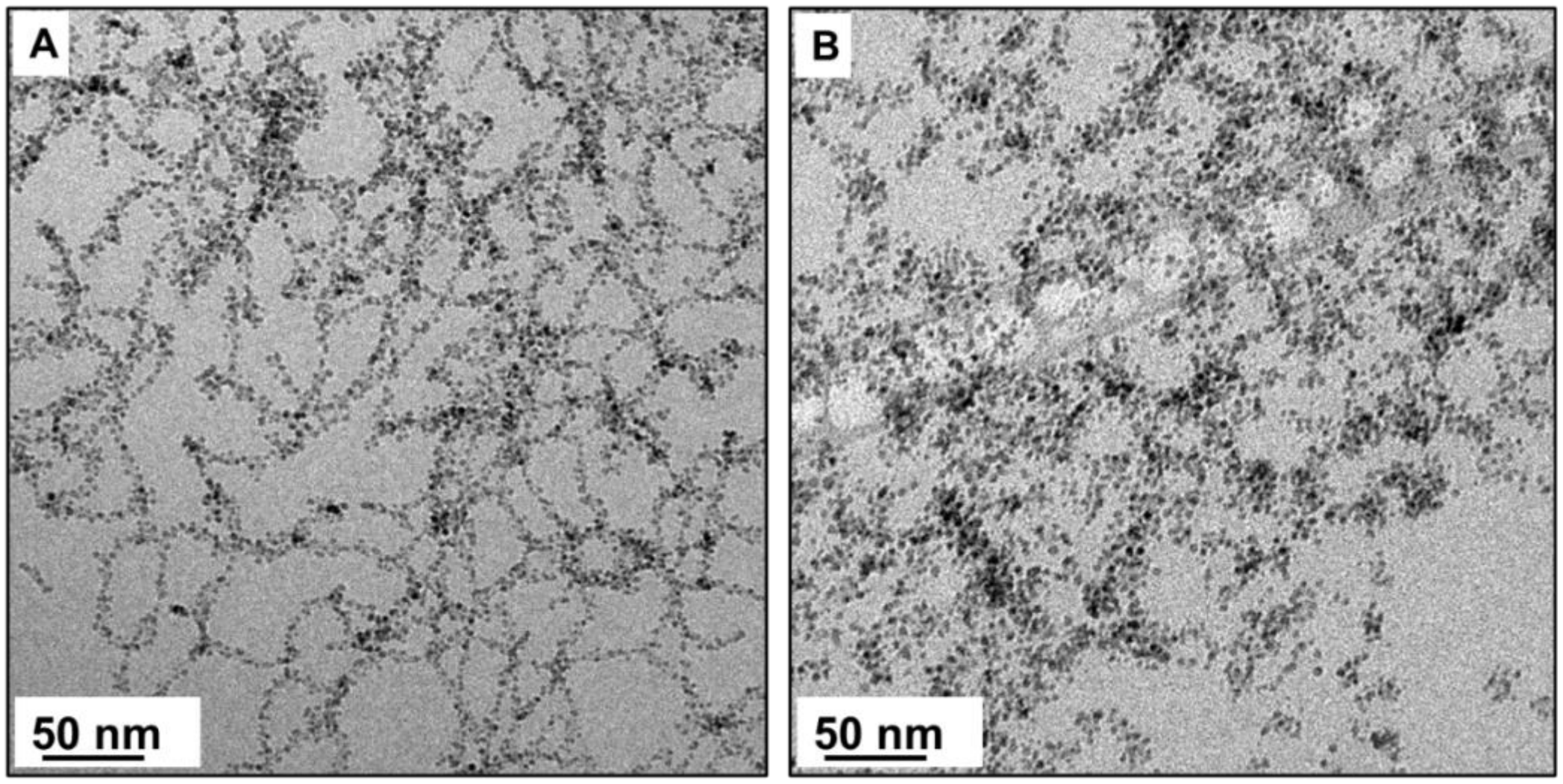

Fig. S21. Cryo-TEM images comparing Cu-induced assemblies formed with different polyoxometalate-SnO_2 nanocrystals. (A) **1** exhibits branched, linear chains attributed to complexation of Cu^{2+} by hexaniobate anions on neighboring particles. (B) **2** forms denser, random aggregates under identical conditions attributed to electrostatic interactions with hydrated Cu^{2+}.

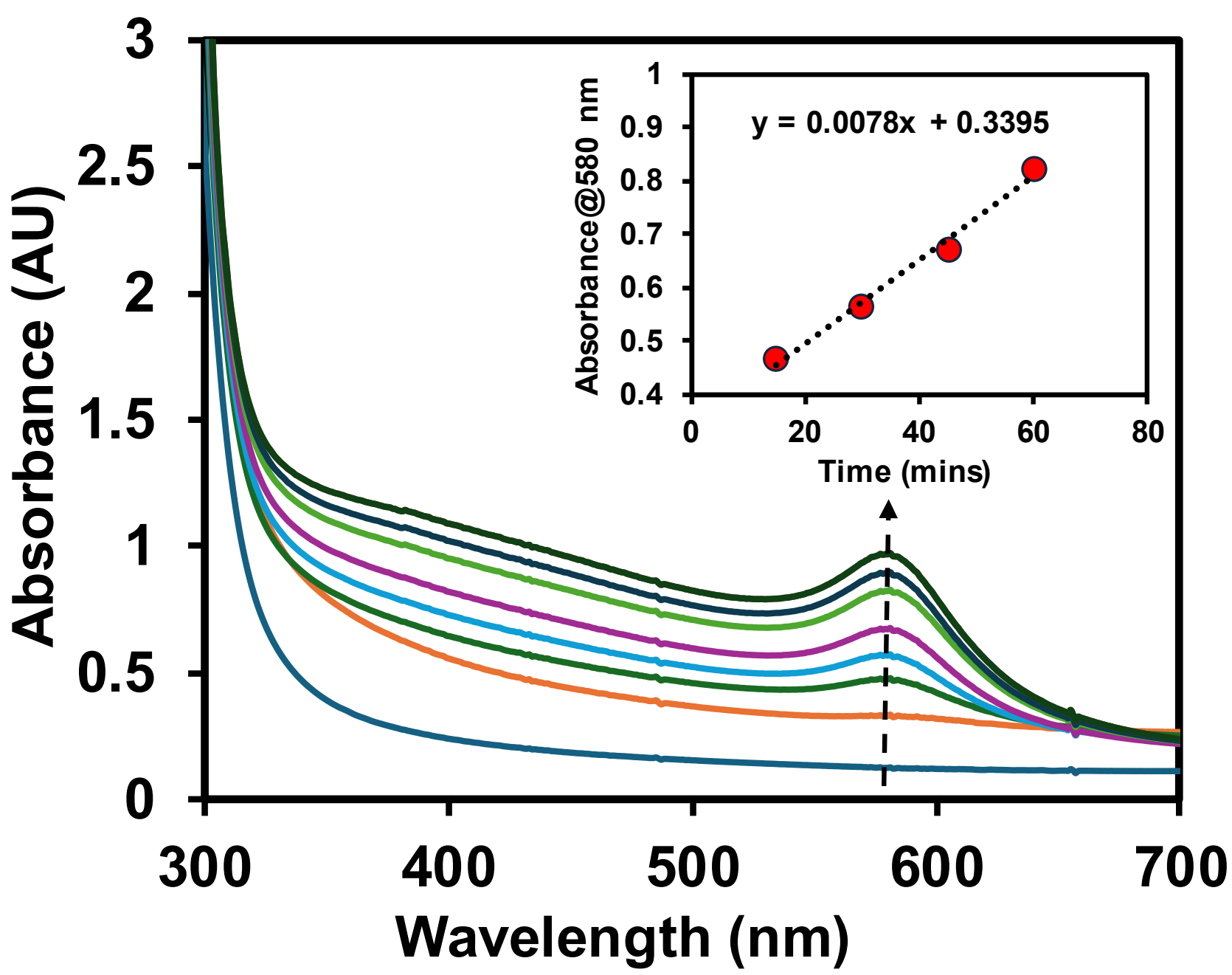

Fig. S22. Time-resolved UV-vis absorption spectra of **1** in the presence of Cu^{2+} under Xe-lamp irradiation (305 nm cut off). A gradual increase in a broad band centered at ~580 nm is observed with irradiation time, corresponding to the surface plasmon resonance (SPR) of Cu^0 nanoparticles. The growth of this feature confirms the photoreduction of Cu^{2+} to Cu^0 mediated by **1**. *Inset:* Time-dependent increase in absorbance at 580 nm, illustrating the kinetics of Cu^0 formation.

Calculation of the concentration of Cu(0) in the solution by using a reported extinction coefficient for the similar-sized Cu(0) NPs having a maximum at 580 nm[10]

Given:

$[Cu^{2+}]_0$ = 0.5 mM = 500 µM, $[1]_0$ = 4 µM, Path length *l = 1 cm*

1) Baseline-corrected plateau absorbance

- $A_0 = 0.12$
- $A_\mathrm{t} = 0.97$

$$\Delta A_\mathrm{t} = A_\mathrm{t} - A_0 = 0.85$$

(2) Particle concentration from the reported extinction coefficient

Using $\varepsilon_{580} = 7.7 \times 10^7\ \mathrm{M^{-1}\ cm^{-1}}$

and $l = 1$ cm.

$$[CuNP] = \frac{\Delta A_\mathrm{t}}{\varepsilon_{580} l} = \frac{0.845785}{(7.7 \times 10^7)(1)} = 1.10 \times 10^{-8}\ \mathrm{M}$$

So:

$$[CuNP] \approx 11\ \mathrm{nM}$$

(3) Converting particle concentration into total Cu(0) concentration (Cu atoms)

Assuming Cu(0) aggregates as a **10 nm** Cu sphere:

radius $r = 5$ nm

Particle volume:

$$V = \frac{4}{3}\pi r^3 = \frac{4}{3}\pi(5 \times 10^{-7})^3 = 5.24 \times 10^{-19}\ \mathrm{cm^3}$$

Cu atomic number density:

$$n_{Cu} \approx 8.47\ \times 10^{22}\ atoms\ cm^{-3}$$

Atoms per particle:

$$N_{\frac{\mathrm{Cu}}{\mathrm{NP}}} = V\, n_{\mathrm{Cu}} = (5.24 \times 10^{-19})(8.47 \times 10^{22})$$

$$\approx 4.43 \times 10^4\ \mathrm{atomsperpatricle}$$

Total Cu(0) concentration (in **mol of Cu atoms per L**):

$$[Cu(0)] = [CuNP] \times N_{\mathrm{Cu/NP}} = (1.10 \times 10^{-8})(4.43 \times 10^4) = 4.87 \times 10^{-4}\ \mathrm{M} = \mathbf{0.487\ mM}$$

Note: Tyagi and co-workers reported a particle-normalized molar extinction coefficient for oleylamine-capped Cu(0) nanoparticles at the plasmon maximum ($\lambda \approx 580$ nm), $\varepsilon_{580} \approx 7.7\times10^{7}$ $M^{-1}cm^{-1}$, determined by combining UV-vis spectroscopy with independent particle/Cu quantification (TEM/ICP-MS). Using this particle-normalized extinction coefficient as an approximation for the in situ-formed Cu(0) plasmonic species, we estimated the concentration of Cu(0) nanoparticles produced under our photocatalytic conditions from the plateau value of the 580 nm band. Baseline correction gives $\Delta A_{\mathrm{t}} = A_{\mathrm{t}} - A_0 = 0.97 - 0.12 = 0.85$ (path length $l = 1$cm), yielding a particle concentration $[CuNP] = \Delta A_{\mathrm{t}}/(\varepsilon_{580} l) \approx 1.10 \times 10^{-8}$M. Assuming a representative 10 nm Cu(0) particle, the number of Cu atoms per particle is $N_{\mathrm{Cu/NP}} \approx 4.4 \times 10^{4}$(density-based estimate), giving a total Cu(0) concentration $[Cu(0)] \approx [CuNP] \times N_{\mathrm{Cu/NP}} \approx 4.9 \times 10^{-4}$M (0.49 mM), which is in close agreement with the initial Cu(II) loading (0.50 mM). This provides an independent optical estimate consistent with near-complete Cu(II) reduction at the plateau. For the calculation purpose, the estimated concentrations of Cu(0) at different time intervals are used in µmols in the main text.

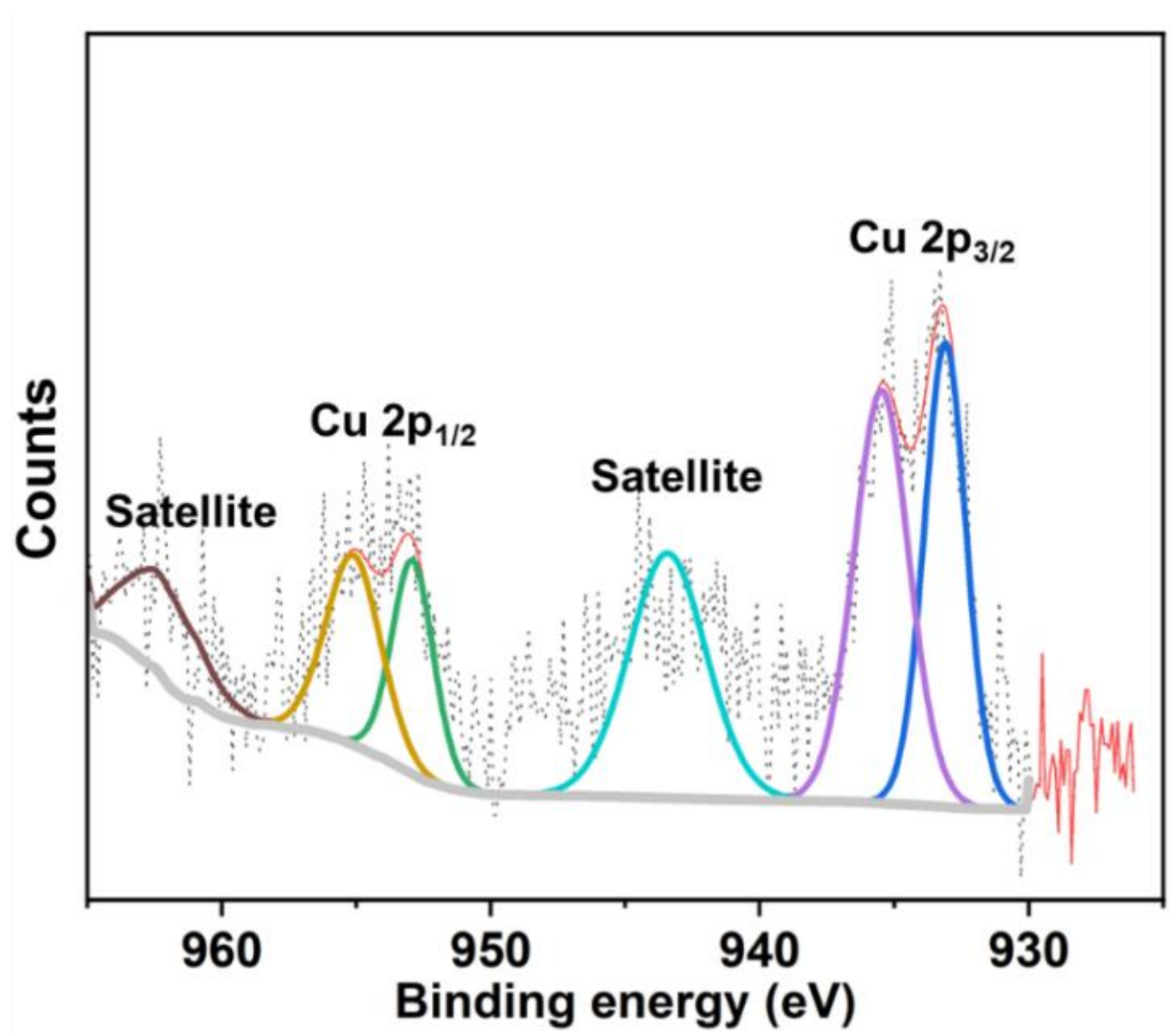

Fig. S23. High-resolution XPS spectra of the Cu 2p region for Cu^{2+}-containing assemblies of **1**.

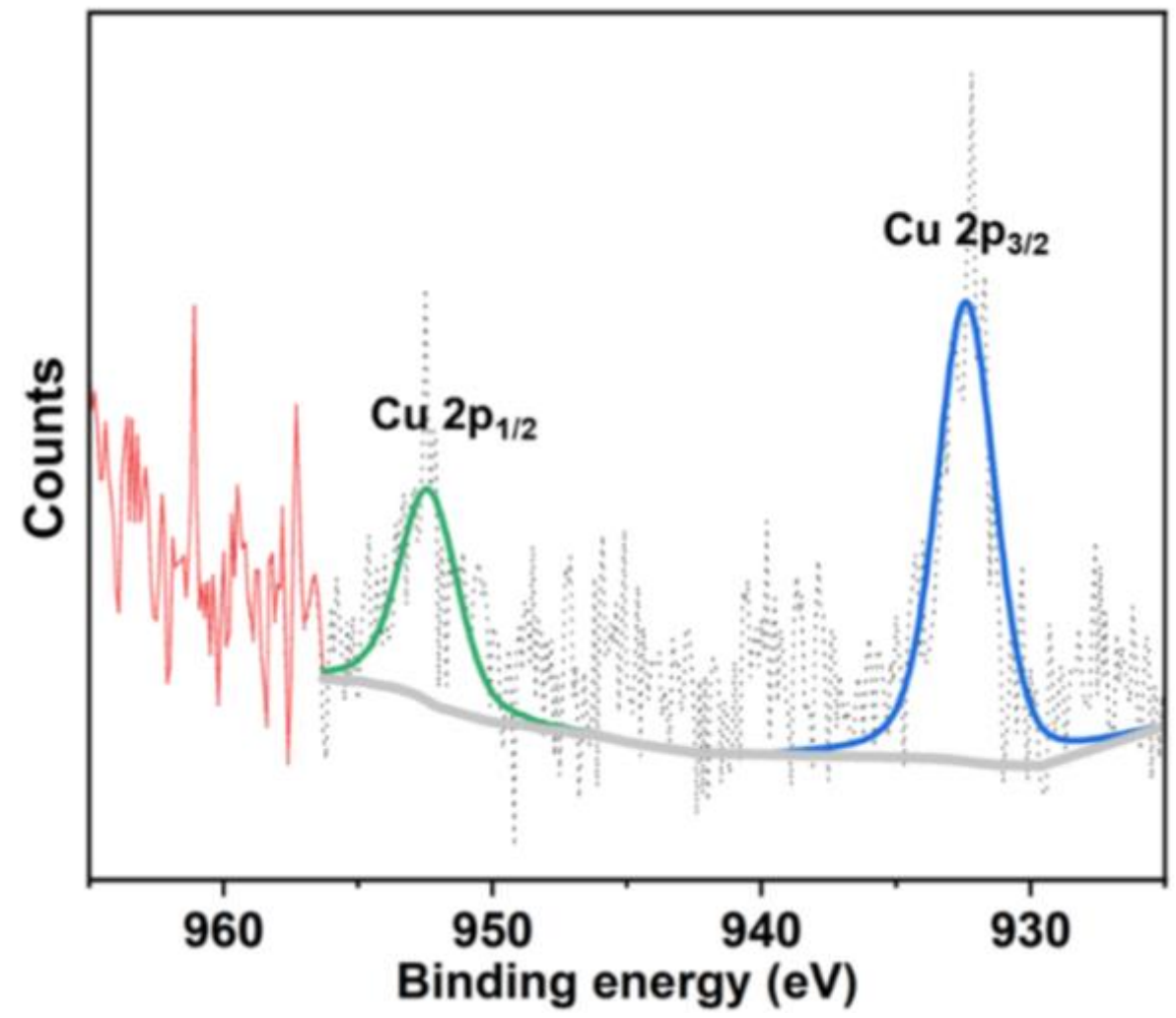

Fig. S24. High-resolution Cu 2p XPS spectrum of a solution containing **1** and Cu^{2+} after photocatalytic Cu^{2+} reduction. The Cu 2p XPS spectrum exhibits characteristic Cu $2p_{3/2}$ and Cu $2p_{1/2}$ peaks at approximately 932 and 952 eV, respectively. Importantly, no prominent shake-up satellite features are observed in the 940-945 eV region, suggesting the absence of Cu(II) species within the signal-to-noise limits of the measurement.

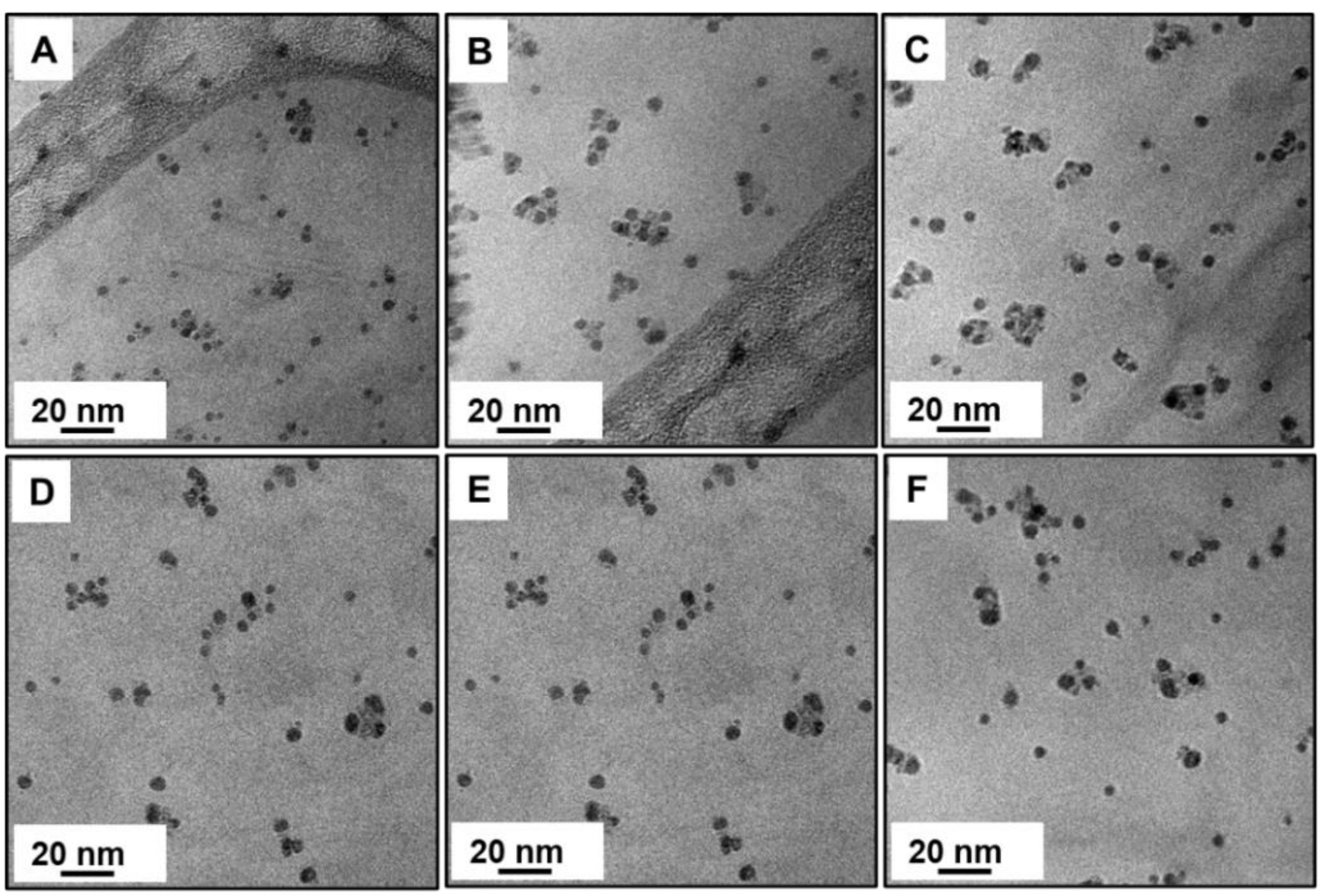

Fig. S25. (A-F) Cryo-TEM images of a solution of **1** containing Cu^{2+} after photocatalytic Cu^{2+} reduction. In addition to individual **1**, larger nanoparticles were observed, indicating the formation of Cu^0 particles likely stabilized in solution by surrounding particles of **1**. Evidence for stabilization of the Cu^0 particles

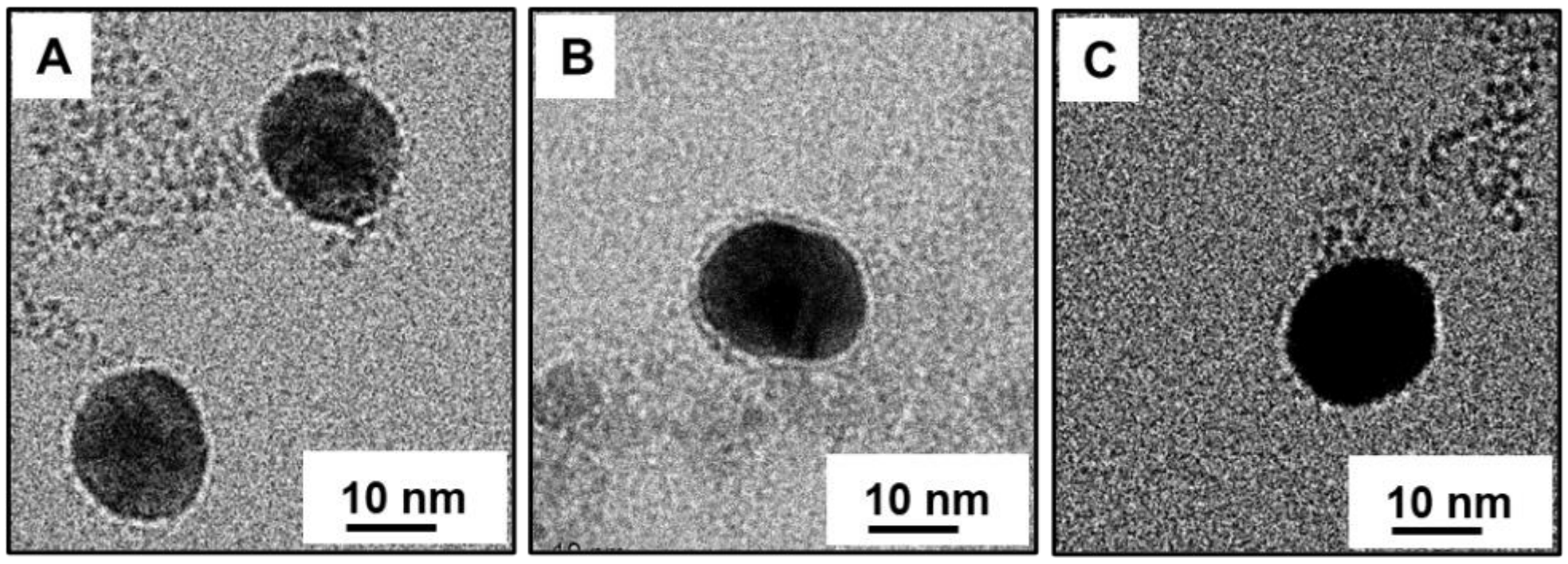

Fig. S26. (A-C) Cryo-TEM images of hexaniobate-complexed CuO NCs[2] on 14 nm Au NPs. Assembly of Nb_6-CuO on 14 nm Au NP was confirmed via cryo-TEM imaging, which visually validated the monolayer structure.

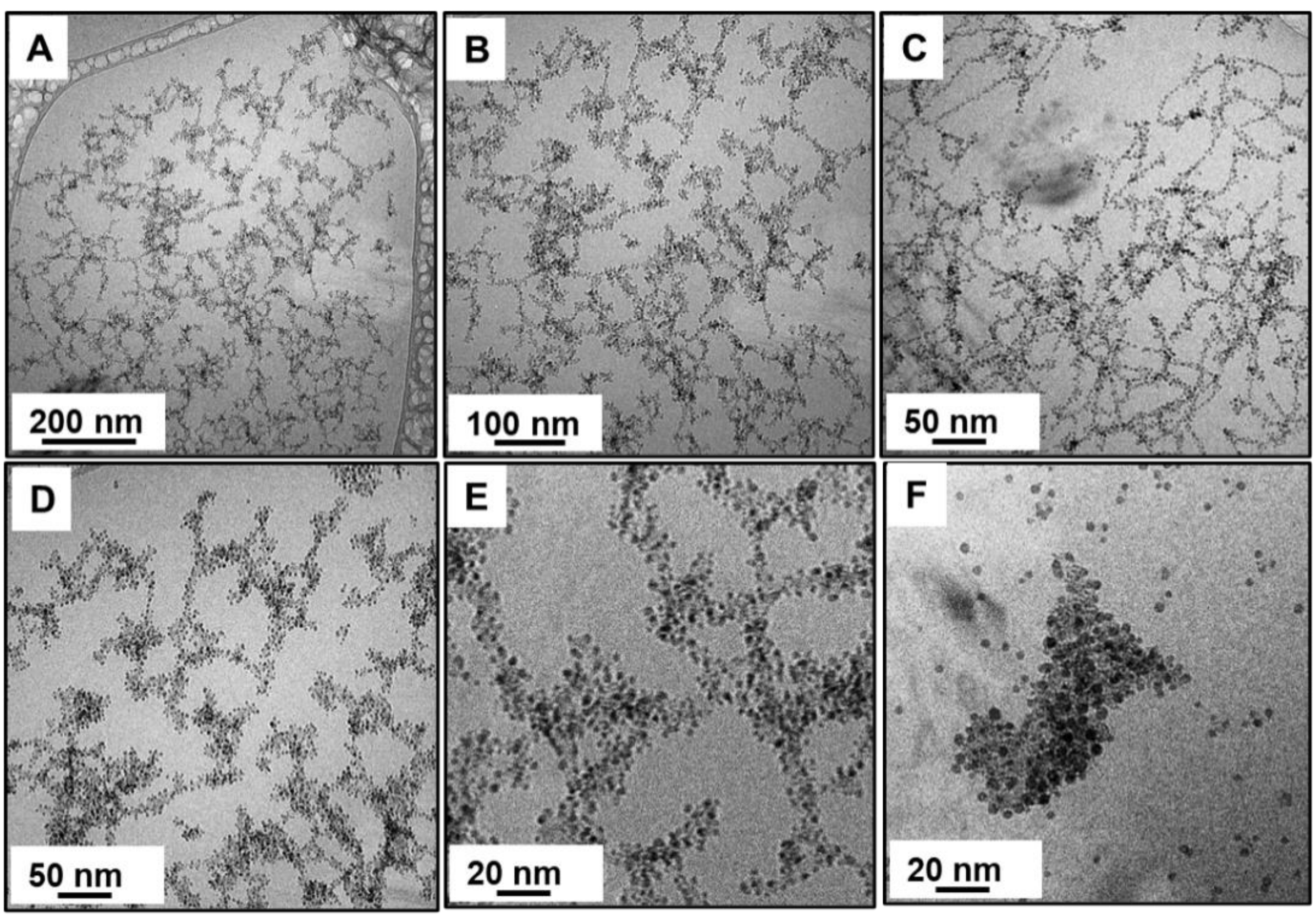

Fig. S27. (A-F) Cryo-TEM images of the reduced solution of **1** containing Cu^{2+} sample after exposure to air, showing reversal of the Cu-induced assemblies.

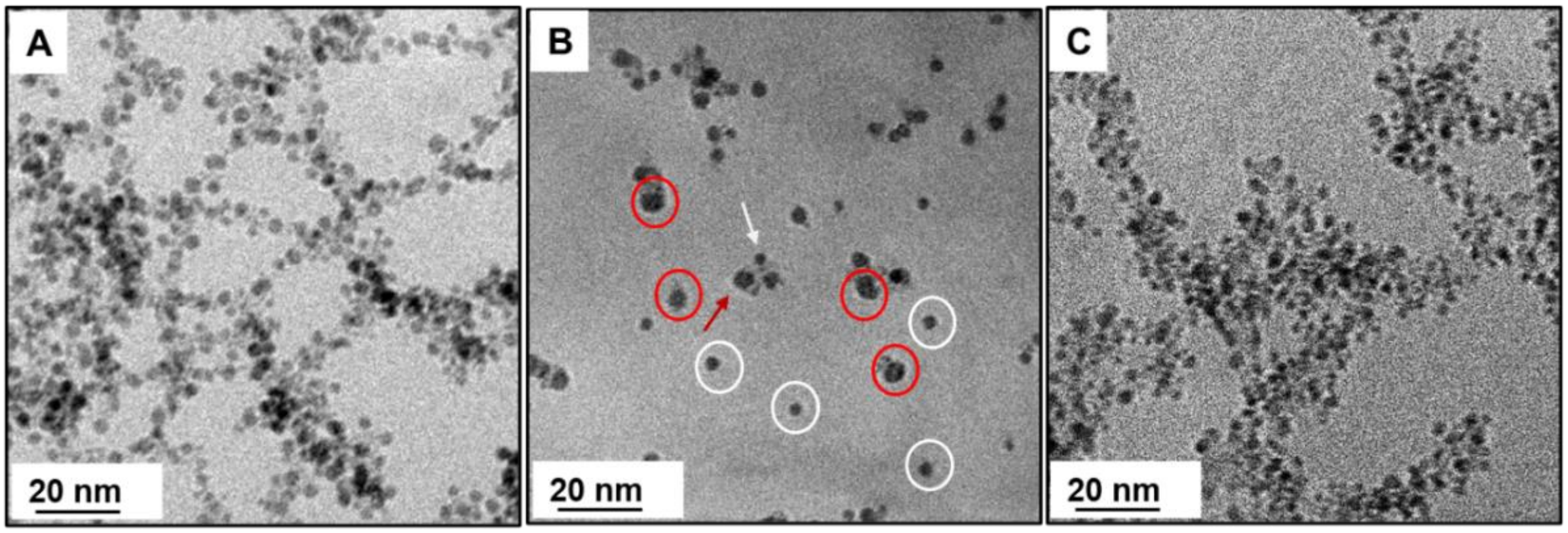

Fig. S28. Cryo-TEM images illustrating reversible Cu-mediated assembly of **1**. (A) Chain-like nanocrystal assemblies before irradiation. (B) After irradiation, reduced Cu^0 domains (red circles) are observed alongside individual **1** (white circles). (C) Upon exposure to air, the assemblies revert to a dispersed, chain-like morphology, demonstrating structural reversibility.

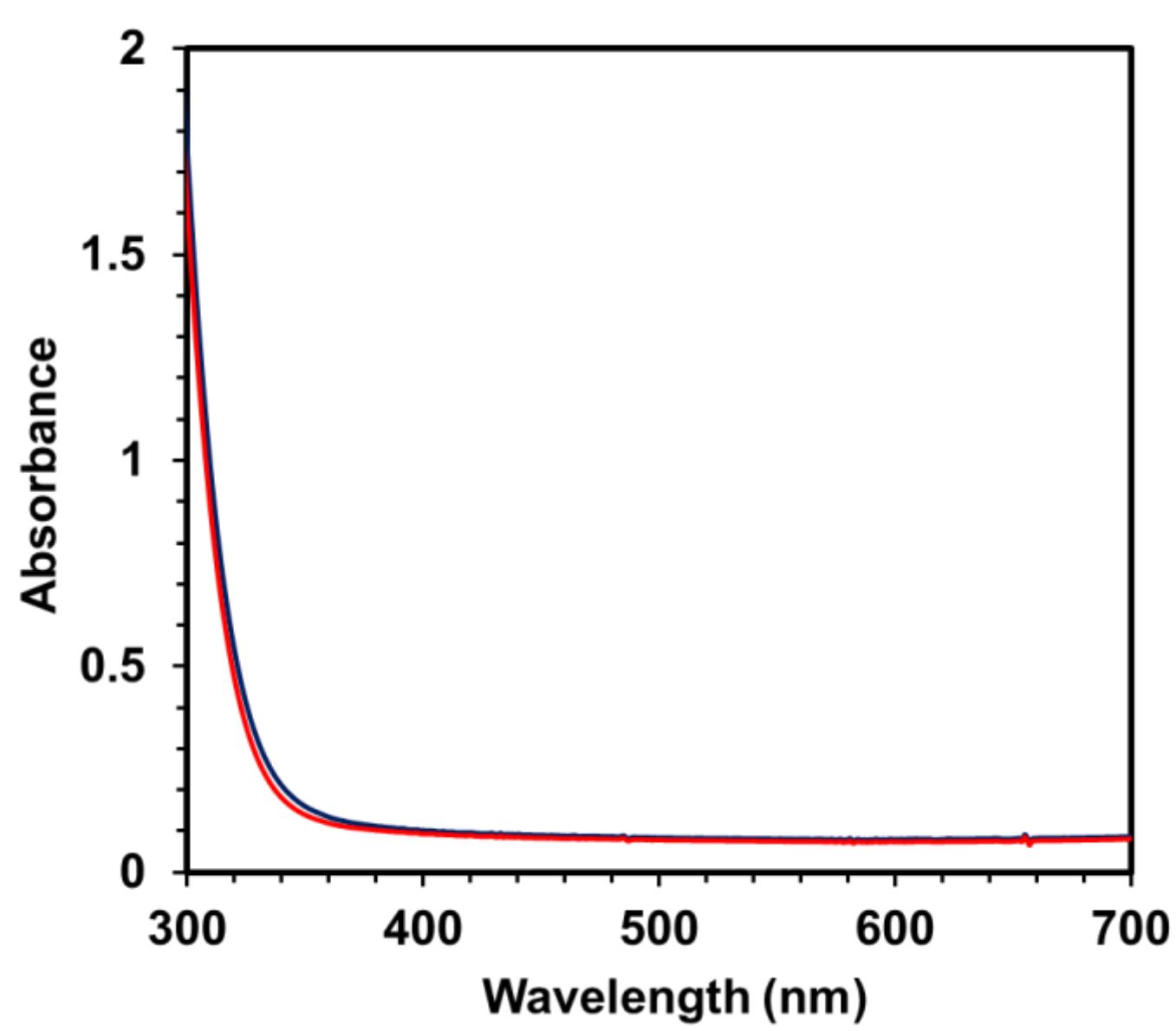

Fig. S29. Control, UV-vis absorption spectra for the Cu^{2+}-containing solution of **2**. The spectra recorded before irradiation (red) and after 60 minutes of irradiation (black) show no emergence of a Cu^0 surface plasmon resonance band, demonstrating that **2** does not promote Cu^{2+} reduction under identical conditions.

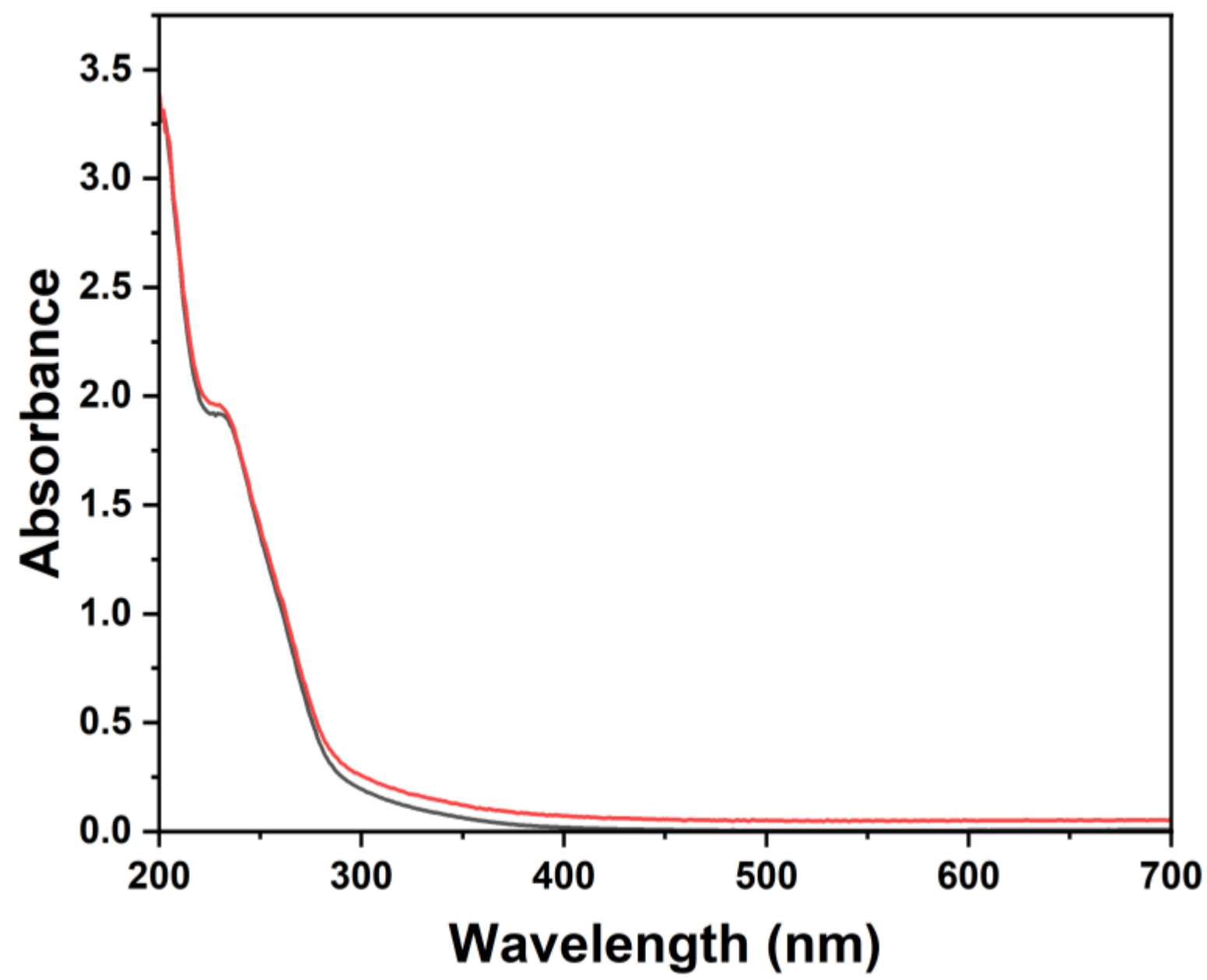

Fig. S30. Control, UV-vis absorption spectra of hexaniobate in the presence of Cu^{2+} recorded before irradiation (black) and after irradiation (red). The absence of a surface plasmon resonance band in the visible region after irradiation indicates that hexaniobate alone does not promote Cu^{2+} reduction under these conditions.

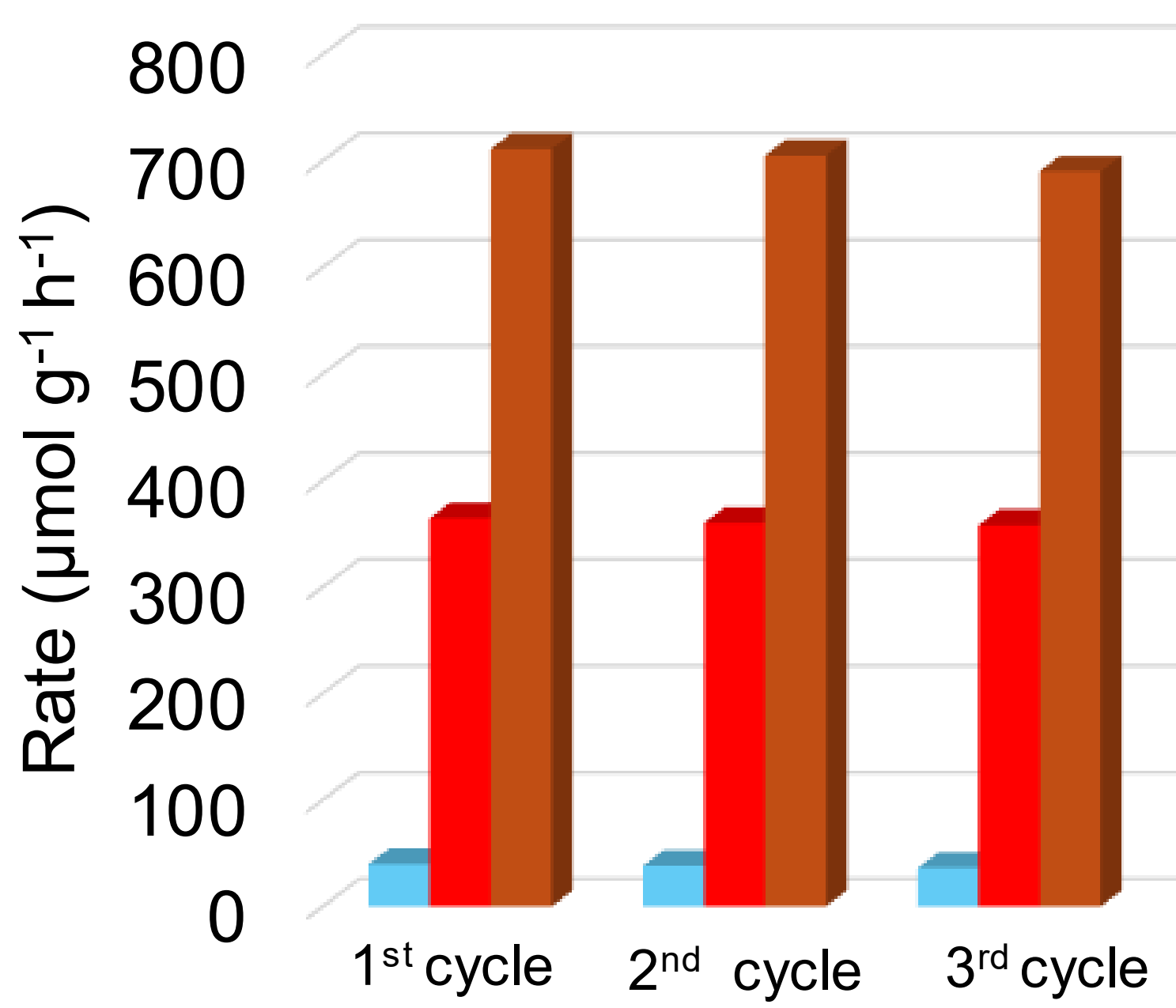

Fig. S31. Recycling performance of **1** over three consecutive cycles. Brown bars correspond to CO, red to O_2, and blue to H_2. The results demonstrate selective CO formation by **1** with sustained activity over multiple cycles.

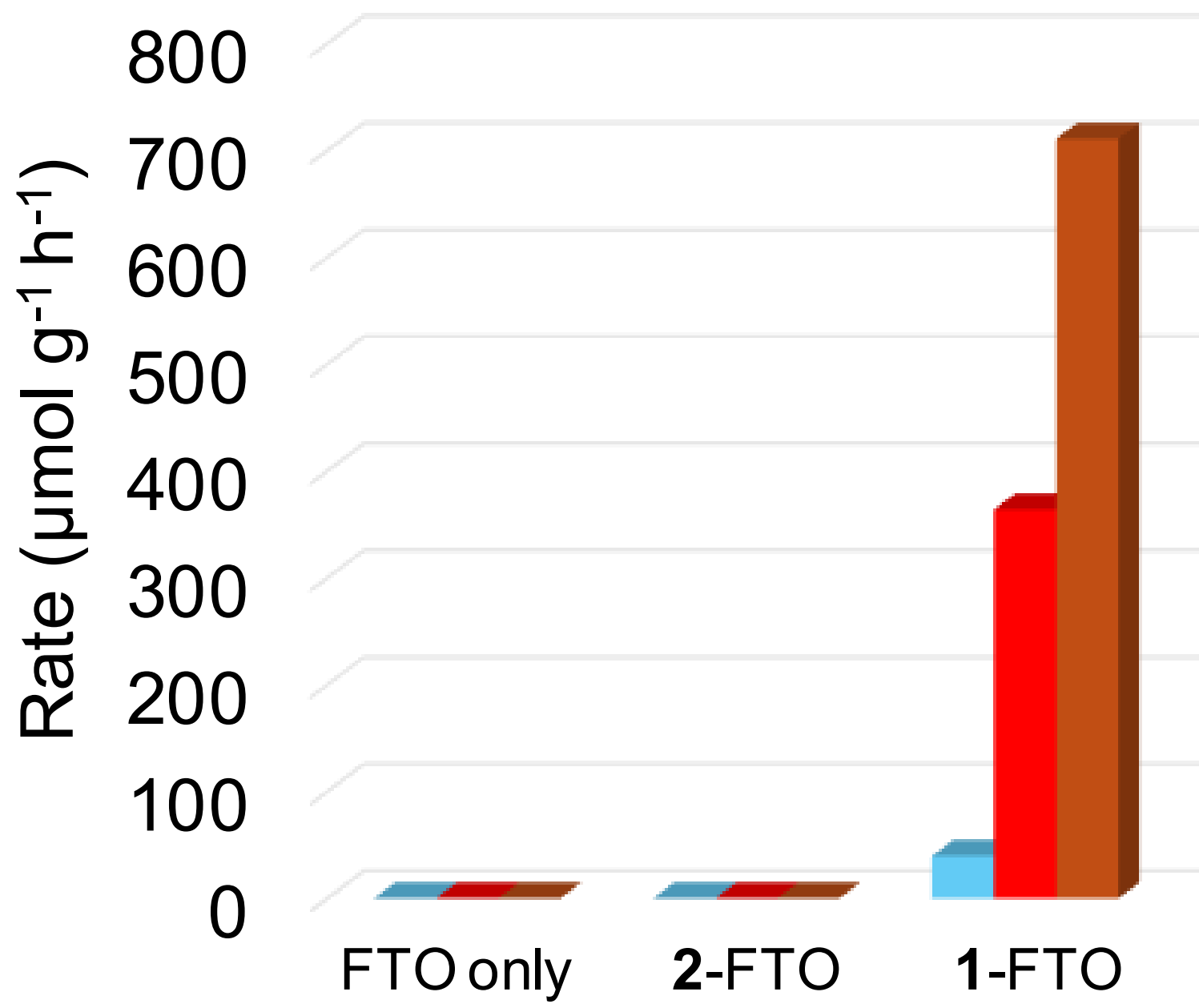

Fig. S32. Photocatalytic CO_2 reduction performance after 4 h by FTO alone, by **2** drop-casted on FTO (**2**-FTO), and by **1** drop-casted on FTO (**1**-FTO).

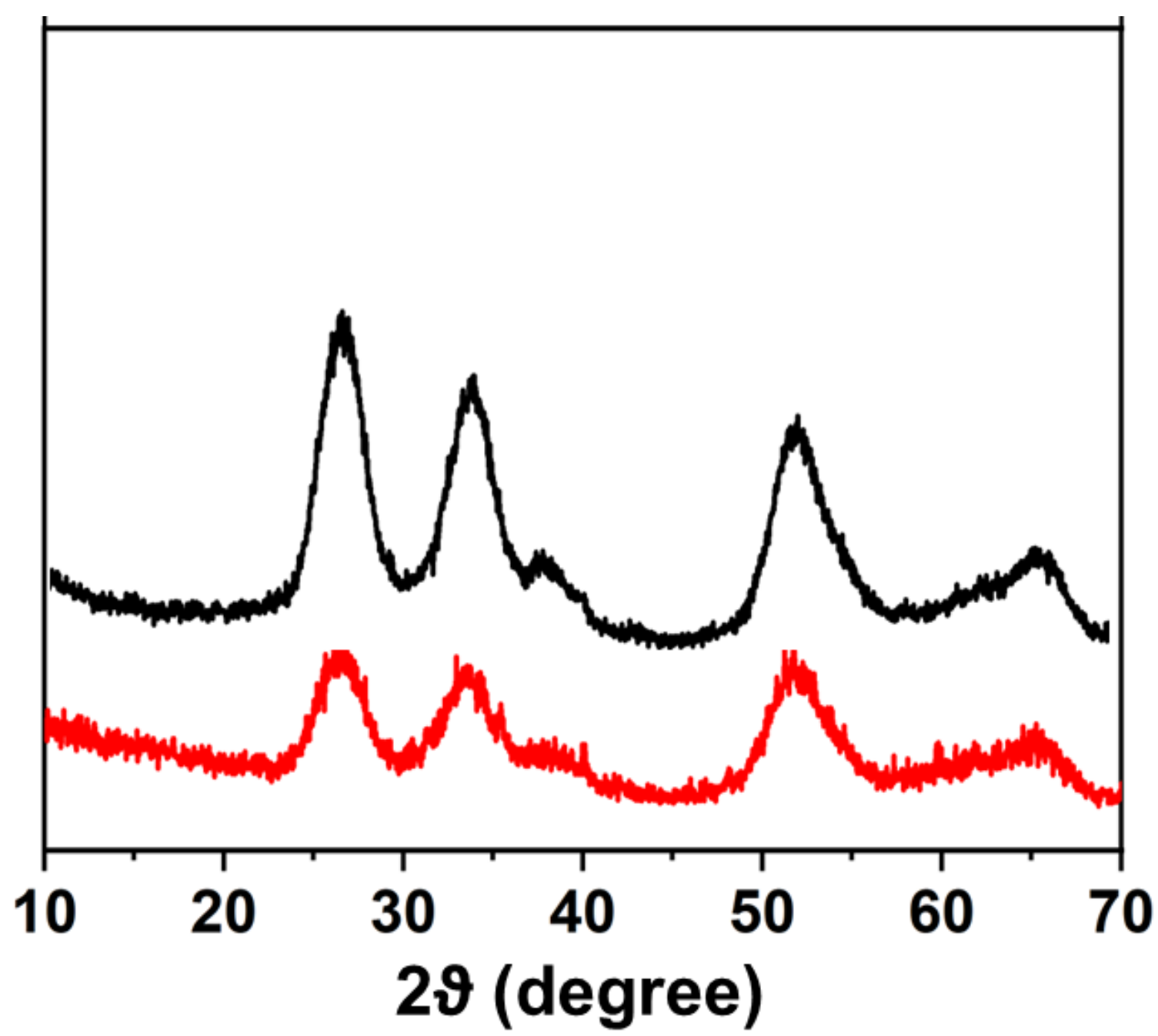

Fig. S33. PXRD of **1** before (black pattern) and after the water splitting reaction (red pattern). No change in PXRD patterns highlights the stability of the catalyst under reaction conditions.

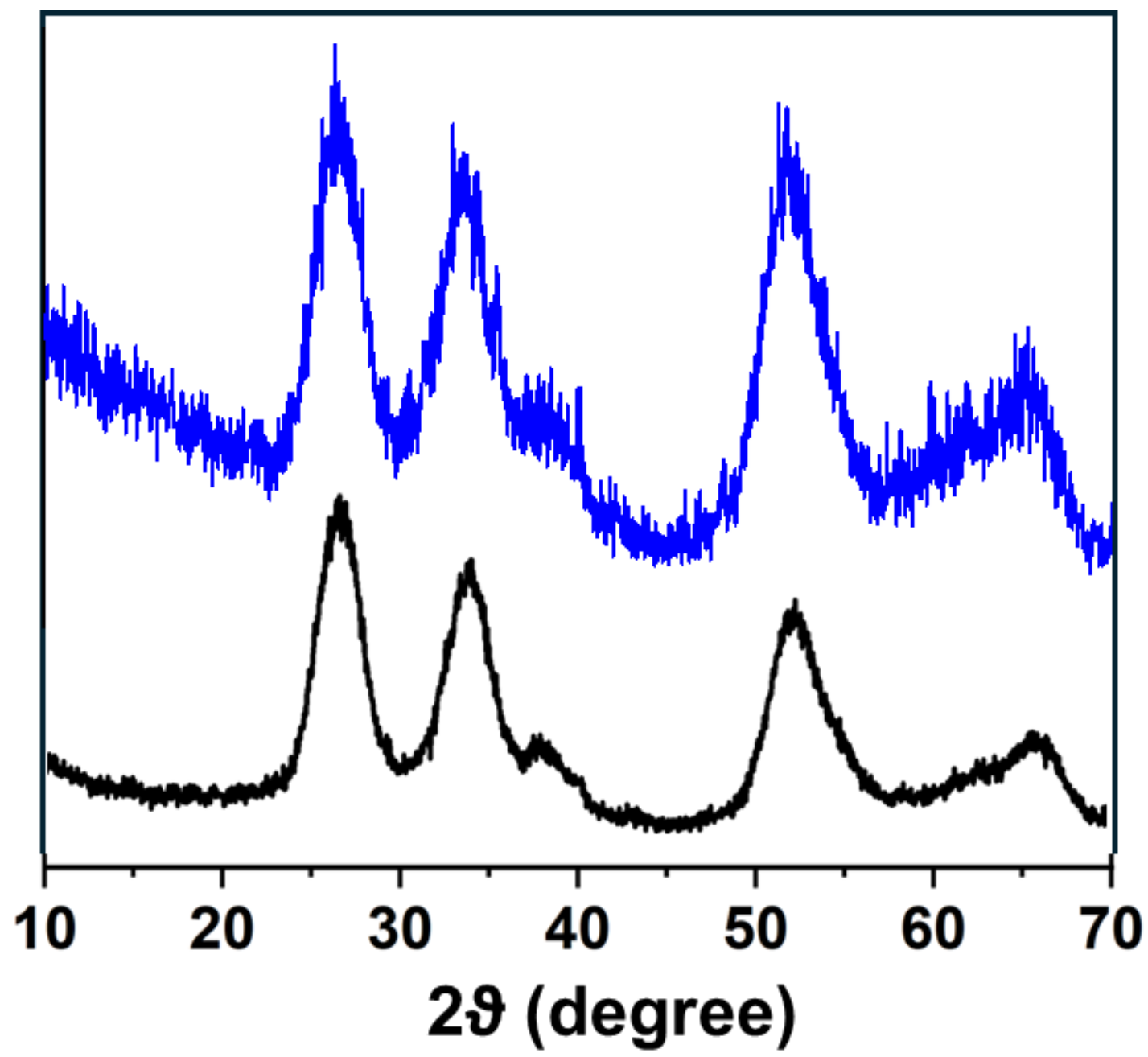

Fig. S34. PXRD of **1** before (black pattern) and after the CO_2 reduction reaction (blue pattern). No change in PXRD patterns highlights the stability of the catalyst under reaction conditions.

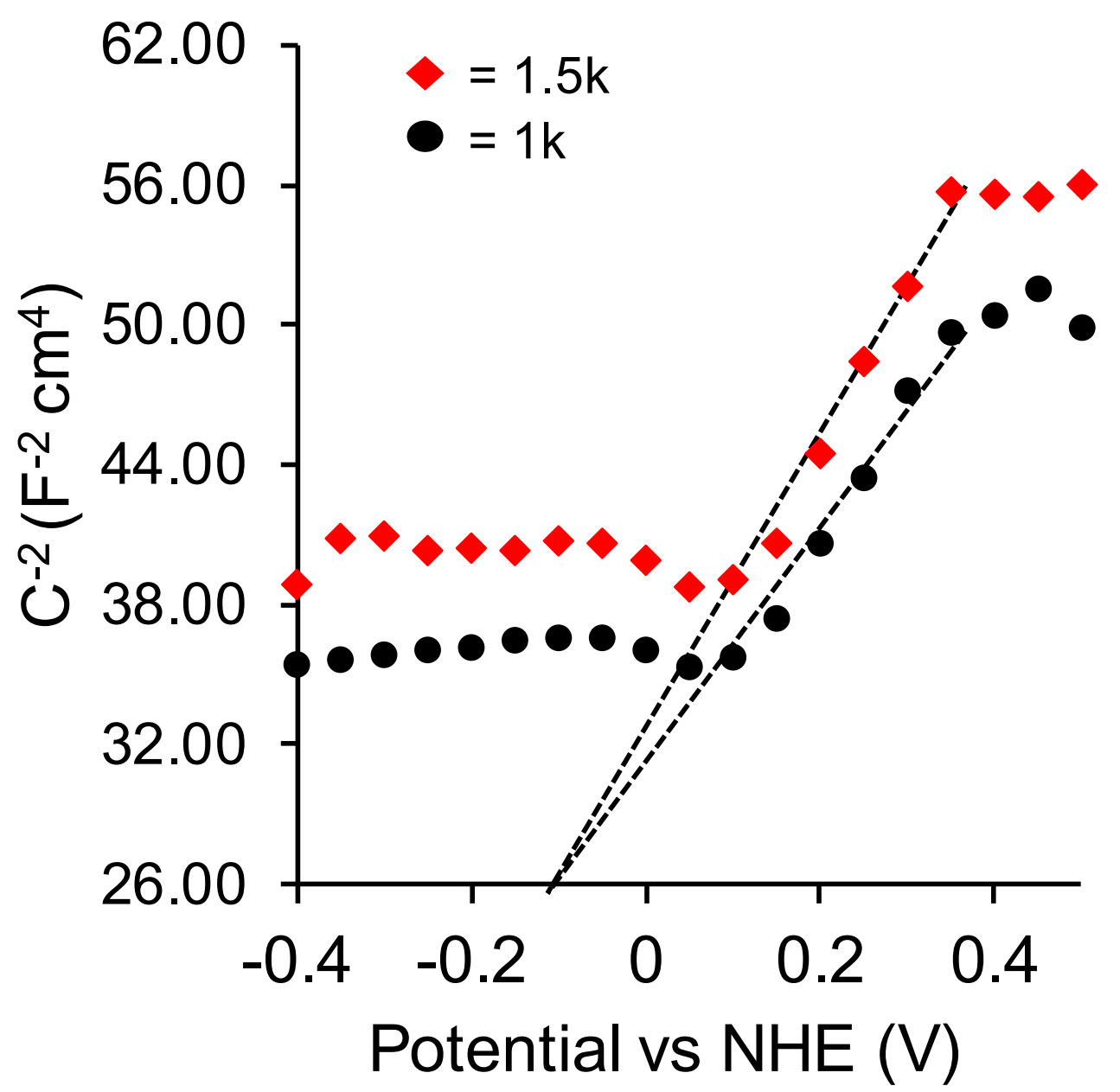

Figure S35. Mott-Schottky plot of **1** at different frequencies. The positive slope of the linear region confirms the n-type semiconducting nature of the **1**. Extrapolation of the linear fit to the potential axis yielded a flat-band potential of approximately -0.10 V vs NHE. For n-type

semiconductors, the CB minimum is generally located close to the flat-band potential; therefore, the conduction band edge of **1** was estimated to be ca. -0.10 V vs NHE.

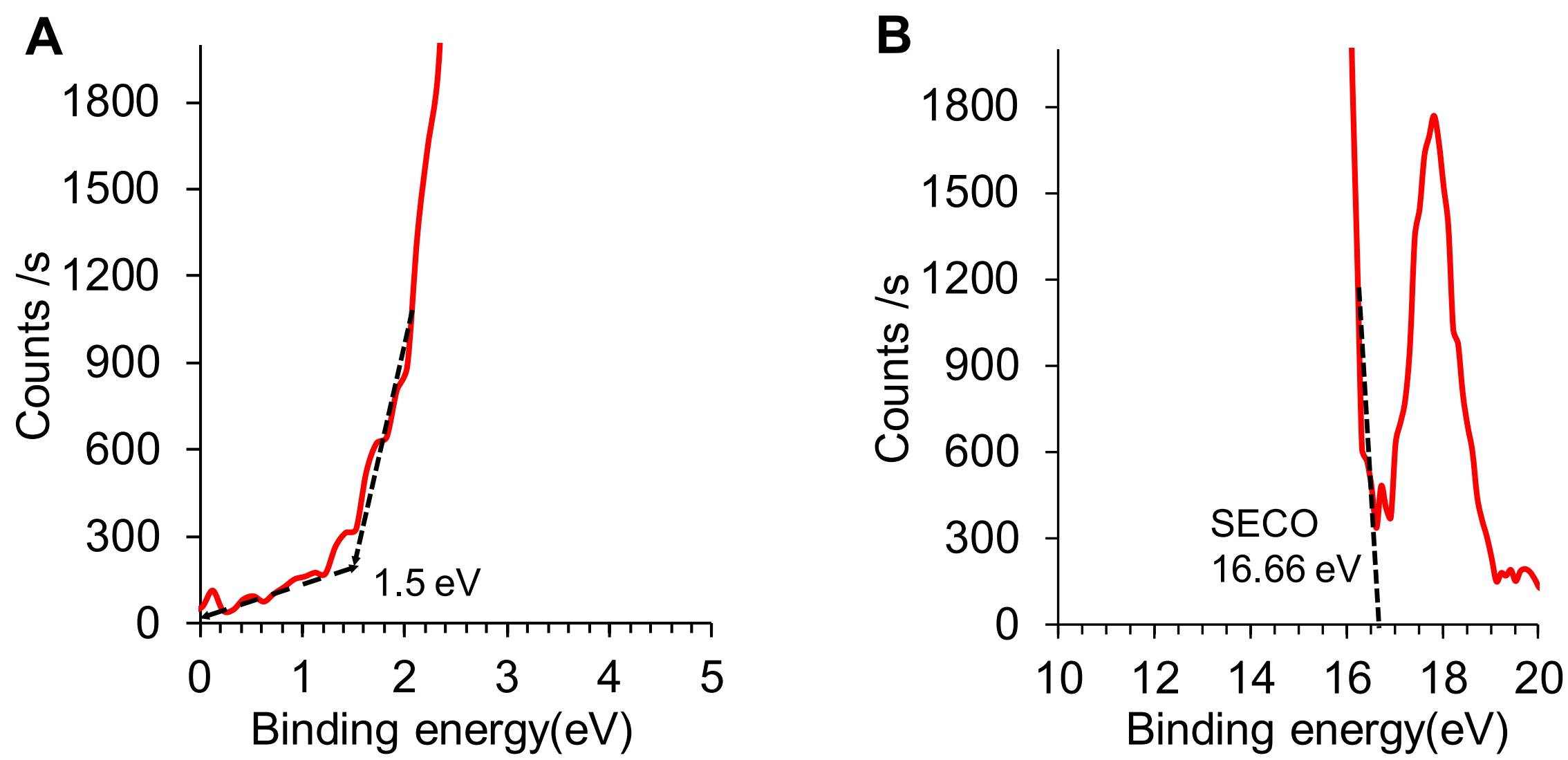

Figure S36. Ultraviolet photoelectron spectroscopy (UPS) analysis of **1**. **(A)** Valence band region showing the valence band onset determined from the extrapolated leading edge of the spectrum. The valence band maximum was estimated to be ca. 1.5 eV below the Fermi level. **(B)** Secondary electron cutoff region used to determine the work function. The cutoff energy obtained from the extrapolated linear edge (16.66 eV) gives a work function of ca. 4.56 eV. Together with the optical band gap of 3.54 eV, these values were used to construct the electronic band diagram of **1**.

Supplementary calculation S1 Calculation of VB and CB positions of 1 from UPS[11]:

The work function was calculated from the secondary electron cutoff (SECO) using:

$$\Phi = h\nu - E_{\text{cutoff}}$$

where $h\nu = 21.22$ eV and $E_{\text{cutoff}} = 16.66$ eV

$$\Phi = 21.22 - 16.66 = 4.56 \text{ eV}$$

The valence band onset from the UPS valence region was estimated to be ca. 1.5 eV below the Fermi level. Therefore, the valence band maximum relative to vacuum was calculated as:

$$E_{\text{VB}} = -(\Phi + E_{\text{onset}})$$
$$E_{\text{VB}} = -(4.56 + 1.50) = -6.06 \text{ eV vs vacuum}$$

Using the optical band gap of $E_g = 3.54$eV, the conduction band minimum was estimated as:

$$E_{\text{CB}} = E_{\text{VB}} + E_g$$
$$E_{\text{CB}} = -6.06 + 3.54 = -2.52 \text{ eV vs vacuum}$$

The energy levels were converted to the NHE scale using:

$$E_{\text{NHE}} = -(E_{\text{vacuum}} + 4.44)$$

Thus:

$$E_{\text{VB}} = -(-6.06 + 4.44) = +1.62 \text{ V vs NHE}$$
$$E_{\text{CB}} = -(-2.52 + 4.44) = -1.92 \text{ V vs NHE}$$

Therefore, the UPS-derived band positions of **1** are:

$$E_{\text{VB}} \approx +1.62 \text{ V vs NHE}$$
$$E_{\text{CB}} \approx -1.92 \text{ V vs NHE}$$

Supplementary Calculation S2. Estimation of the number of ligand-accessible Sn atoms at the NC surface

Considering a spherical SnO_2 NC with an average diameter of 3.2 nm, the radius is:

$$r = 1.6 \text{ nm}$$

The average number of Sn atoms per nanocrystal is:

$$N_{\text{Sn,total}} = 480 \pm 90$$

The surface was approximated as an outer shell of thickness:

$$t = 0.25\text{-}0.30 \text{ nm}$$

The fraction of Sn atoms in the surface shell was estimated as:

$$f_{\text{surface}} = 1 - \left(\frac{r - t}{r}\right)^3$$

For $t = 0.25$nm:

$$f_{\text{surface}} = 1 - \left(\frac{1.6 - 0.25}{1.6}\right)^3 = 0.399$$

$$N_{\text{Sn,surface}} = 0.399 \times 480 \approx 191$$

For $t = 0.30$nm:

$$f_{\text{surface}} = 1 - \left(\frac{1.6 - 0.30}{1.6}\right)^3 = 0.463$$

$$N_{\text{Sn,surface}} = 0.463 \times 480 \approx 222$$

This uncertainty gives an estimated value of 200 ± 20 ligand-accessible Sn atoms at the NC surface.

Supplementary calculation S3. Influence of hexaniobate on surface-SnO_2 O atoms

The relative abundance of surface facets in rutile SnO_2 nanoparticles is governed by surface energetics. Surface science investigations have consistently shown that the {110} surface is the lowest-energy facet of rutile SnO_2, followed by higher-energy surfaces such as {101}, {100}, and {001}, with the following order of relative surface energies:[12-14]

$$\gamma_{\{110\}} < \gamma_{\{100\}} < \gamma_{\{101\}} < \gamma_{\{001\}}$$

With representative values ($J\cdot m^{-2}$):[15] $\gamma_{\{110\}} = 1.04$, $\gamma_{\{100\}} = 1.14$, $\gamma_{\{101\}} = 1.33$ and $\gamma_{\{001\}} = 1.72$.

These values indicate that {110} facets should represent the dominant surface environment of rutile SnO_2, while the higher-energy facets contribute as secondary or truncating surfaces. Therefore, for an order-of-magnitude estimate, the surface of the 3.2 nm SnO_2 nanocrystal can be treated as being composed primarily of low-energy Sn-terminated rutile facets. Based on the particle size, the number of ligand-accessible surface Sn atoms is estimated to be approximately 200. This value is consistent with the experimentally determined loading of ca. 50 hexaniobate ligands per SnO_2 nanocrystal and the topotactic model, in which each hexaniobate ligand can interact with approximately four surface Sn atoms through oxo-donor coordination. Thus:

$$50 \text{ ligands} \times 4 \text{ Sn sites per ligand} \approx 200 \text{ surface Sn sites}$$

Each surface Sn center is associated with approximately four SnO_2-derived *surface planar* O atoms in its local coordination environment (with the O pointing outwards from the crystal being replaced by terminal or bridging O from hexaniobate, and one O pointing inward towards the crystal). However, these O atoms are not unique to a single Sn center; they are shared between neighboring Sn atoms in the rutile lattice. Therefore, the number of unique surface-Sn associated O atoms is:

$$N_O \approx \frac{200 \times 4}{2} = 400$$

This gives an interfacial SnO_2-derived oxygen population on the order of ~ 400 atoms. This value should not be interpreted as an exact crystallographic count, but rather as a chemically reasonable estimate showing that coordination of ca. 50 hexaniobate ligands, each capable of engaging approximately four surface Sn sites, is compatible with near-saturation of the ligand-accessible SnO_2 surface and formation of an extended surface-localized Sn-O/Nb-O-Sn electronic network.

References

(1) Kong, X.; Hu, D.; Wen, P.; Ishii, T.; Tanaka, Y.; Feng, Q. Transformation of potassium Lindquist hexaniobate to various potassium niobates: solvothermal synthesis and structural evolution mechanism. *Dalton Trans.* **2013**, *42*, 7699-7709.

(2) Kumar Tiwari, C.; Roy, S.; Tubul-Sterin, T.; Baranov, M.; Leffler, N.; Li, M.; Yin, P.; Neyman, A.; Weinstock, I. A. Emergence of Visible-Light Water Oxidation Upon Hexaniobate-Ligand Entrapment of Quantum-Confined Copper-Oxide Cores. *Angew. Chem. Int. Ed.* **2023**, *62*, e202213762.

(3) Baranov, M.; Mathew, J.; Kar, A.; Leffler, N.; Joshi, A.; Roy, S.; Gan-Or, G.; Ezersky, V.; Král, P.; Weinstock, I. A. Ion-Size Controlled Non-Classical Crystallization of Metal-Oxide Nanoparticles Covered with a Few Highly Charged Ligands. *J. Am. Chem. Soc.* **2026**. *148*, 4373–4384

(4) Zhang, G.; Wang, F.; Tubul, T.; Baranov, M.; Leffler, N.; Neyman, A.; Poblet, J. M.; Weinstock, I. A. Complexed Semiconductor Cores Activate Hexaniobate Ligands as Nucleophilic Sites for Solar-Light Reduction of CO2 by Water. *Angew. Chem. Int. Ed.* **2022**, *61*, e202213162.

(5) Ben Haj Othmen, W.; Sieber, B.; Elhouichet, H.; Addad, A.; Gelloz, B.; Moreau, M.; Szunerits, S.; Boukherroub, R. Effect of high Fe doping on Raman modes and optical properties of hydrothermally prepared SnO2 nanoparticles. *Mater. Sci. Semicond. Process.* **2018**, *77*, 31-39.

(6) Giubileo, F.; Faella, E.; Pelella, A.; Kumar, A.; Capista, D.; Passacantando, M.; Kim, S. S.; Di Bartolomeo, A. SnO2 Nanofibers Network for Cold Cathode Applications in Vacuum Nanoelectronics. *Adv. Electron. Mater.* **2022**, *8*, 2200237.

(7) Li, C.; Shu, X.; Zhang, J.; Delgado, J.; Bharathan, P.; Wang, Y.; Wang, C.; Fang, J. Controlled Synthesis of SnO_2 Nanocrystals with Tunable Band Gaps. *Precis. Chem.* **2025**, *3*, 463-469.

(8) Volosin, A. M.; Sharma, S.; Traverse, C.; Newman, N.; Seo, D.-K. One-pot synthesis of highly mesoporous antimony-doped tin oxide from interpenetrating inorganic/organic networks. *J. Mater. Chem.* **2011**, *21*, 13232-13240.

(9) Izydorczyk, W.; Izydorczyk, J. Structure, Surface Morphology, Chemical Composition, and Sensing Properties of SnO2 Thin Films in an Oxidizing Atmosphere. *Sensors* 2021, 21, 574.

(10) Tyagi, S.; Kashyap, R. K.; Dhankhar, A.; Pillai, P. P. Plasmon-powered chemistry with visible-light active copper nanoparticles. *Chem. Sci.* **2024**, *15*, 16997-17006.

(11) Whitten, J. E. Ultraviolet photoelectron spectroscopy: Practical aspects and best practices. *Appl. Surf. Sci. Adv.* **2023**, *13*, 100384.

(12) Batzill, M.; Diebold, U. The surface and materials science of tin oxide. *Prog. Surf. Sci.* **2005**, *79* (2), 47-154.

(13) Ghica, C.; Stefan, M.; Stanoiu, A.; Simion, C. E.; Vlaicu, I. D.; Apostol, N. G.; Mihalcea, C. G.; Iacoban, A. C.; Florea, O. G.; Bulat, S.; Ghica, D. Tailoring surface defects and faceting in SnO_2 nanocrystals to improve their NO2 sensing potential. *Surf. Interfaces.* **2025**, *72*, 107212.

(14) Li, G.; Li, S.; Han, Z.-K.; Zou, C.; Wu, H.; Yuan, W.; Zhu, B.; Gao, Y.; Yang, H.; Zhang, Z.; Wang, Y. In Situ Resolving the Atomic Reconstruction of SnO_2 (110) Surface. *Nano Lett.* **2021**, *21*, 7309-7316.

(15) Oviedo, J.; Gillan, M. J. Energetics and structure of stoichiometric SnO_2 surfaces studied by first-principles calculations. *Surf. Sci.* **2000**, *463*, 93-101.
